# A protein–foldamer supramolecular synthon for self-assembled hybrid architectures

**DOI:** 10.1038/s41557-026-02222-6

**Published:** 2026-08-17

**Authors:** Johannes Sigl, Vasily Morozov, Lingfei Wang, Janine Sachs, Eric Merlet, Eric Largy, Niklas Geue, Nicklas Österlund, Sunbum Kwon, Florian Sanchez, Léo Candela, Simon Huet, Kevin Pagel, Yann Ferrand, Céline Douat, Cameron D. Mackereth, Ivan Huc

**Affiliations:** 1 Department of Pharmacy, Ludwig-Maximilians-Universität München, Munich, Germany; 2 Univ. Bordeaux, CNRS, Bordeaux INP, CBMN, UMR 5248, Pessac, France; 3 Univ. Bordeaux, CNRS, INSERM, ARNA, UMR 5320, U1212, Bordeaux, France; 4 Department of Biology, Chemistry, and Pharmacy, Freie Universität Berlin, Berlin, Germany; 5 Department of Molecular Physics, Fritz Haber Institute of the Max Planck Society, Berlin, Germany; 6 Affilogic SAS, Nantes, France; 7 Present Address: Department of Chemistry, Chung-Ang University, Seoul, Republic of Korea

**Keywords:** Proteins, Self-assembly

## Abstract

Constructing artificial assemblies that combine proteins and synthetic ligands has been hampered by the lack of protein–ligand interfaces that are sufficiently large and organized to enable precise structural control. Here ribosome display selection is used to identify a protein that binds a helical aromatic foldamer both tightly and selectively through a sizeable surface area. We used this complex as a supramolecular synthon to create well-defined hybrid foldamer–protein architectures. Examples include foldamers that bind two proteins and hold them at a precise distance, proteins that bind two foldamers and crystals in which proteins and foldamers are connected in cyclic or infinite arrays. The modularity of aromatic foldamers brings a further dimension to protein-based assemblies.

**Figure:**
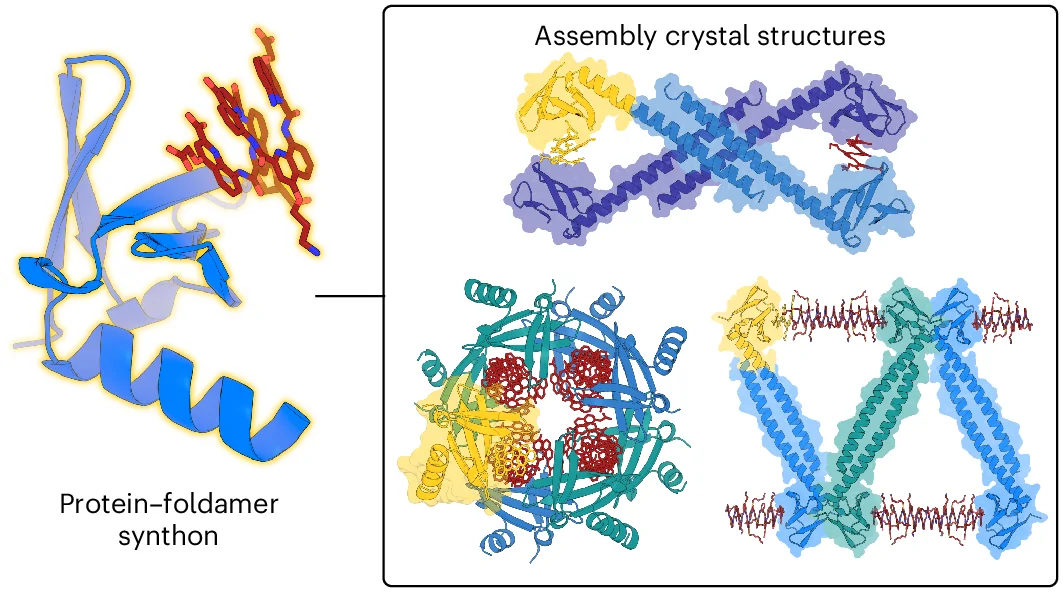

## Abstract (web summary)

A stable and modular artificial protein–foldamer helical supramolecular synthon enables the assembly of ordered architectures, in which the foldamer dictates protein orientation and spacing on the nanoscale.

## Main

Artificial protein assemblies may be constructed by programming protein–protein interaction interfaces using either natural or de novo-designed proteins^1–6^. Rationally organizing the spatial assembly of proteins using synthetic molecules^6^, instead of other proteins, is also desirable as illustrated by current efforts in the development of so-called molecular glues^7,8^, proteolysis targeting chimeras (PROTACs)^9,10^ and hybrid protein frameworks^11–13^. However, the co-assembly of proteins and synthetic molecules remains challenging to achieve with high structural precision. It has been limited by the lack of supramolecular synthons with a sufficiently large and organized protein–ligand interface for architectural control. Thus, synthetic macrocycles and molecular tweezers that bind to protein surfaces have been used to organize the packing of proteins in the solid state but their binding modes are variable^11,13^. Conversely, small molecule ligands that selectively bind protein pockets may not prevent hinge motions, hampering the precise positioning of a large appendage such as another protein. Instead, flexible linkers are generally used, which reduces structural control^8^. In this Article, we introduce a complex between a synthetic helical aromatic foldamer and a 7-kDa artificial protein identified by ribosome display selection. The complex can serve as a supramolecular synthon for discrete aggregate and lattice construction. As solid-state and solution structures reveal, the binding interface is sizeable (>700 Å^2^), stable yet dynamic, and well-defined, allowing for the further elaboration of both the foldamer and protein components and for the construction of hybrid assemblies in which proteins are held at precise positions in space by synthetic foldamers.

**Fig. 1:**
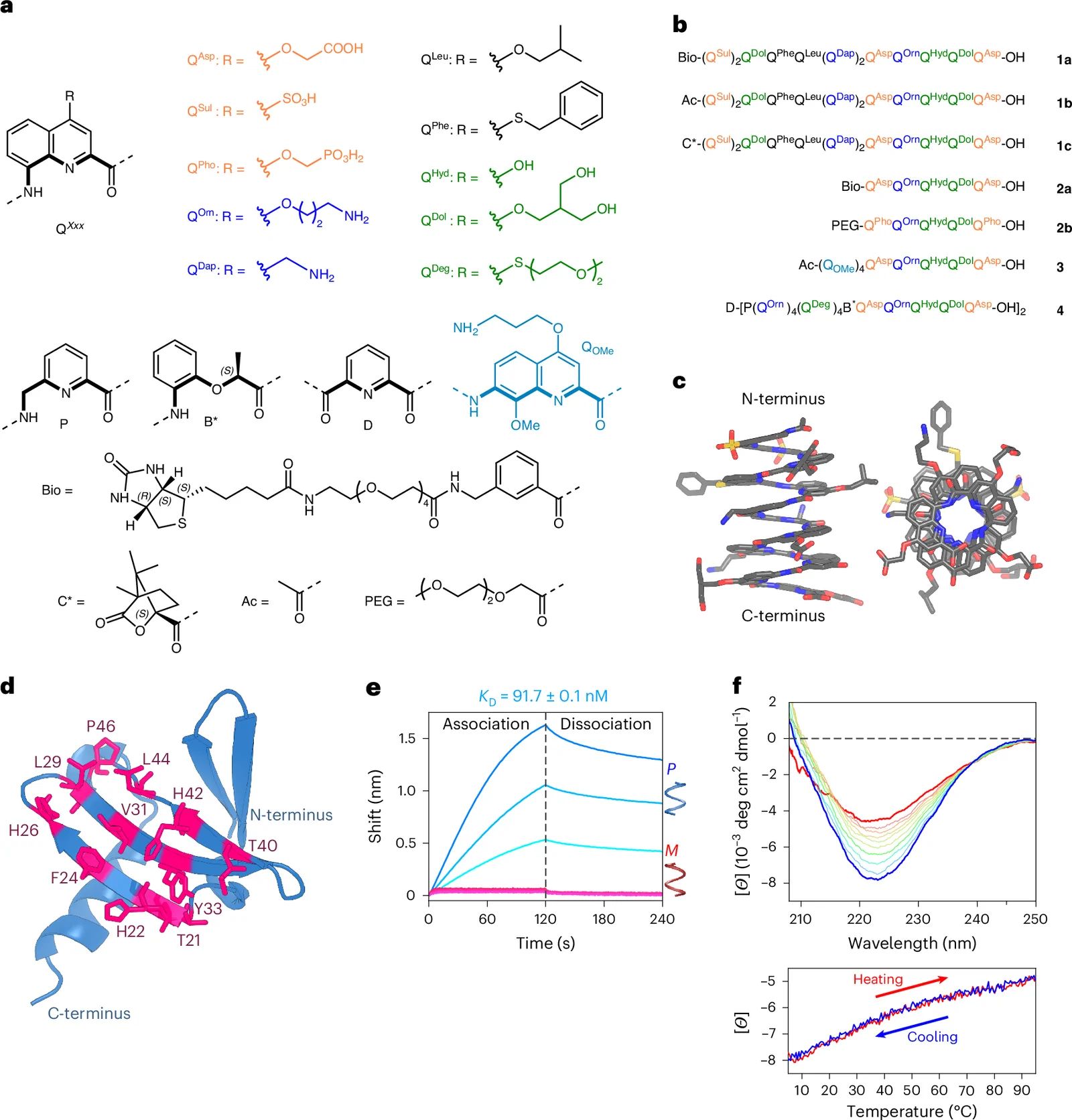
Foldamer building blocks, sequences and display selection results. **a**, Monomer building blocks for AOFs with their respective letter code used in this study. Asp, aspartate-like side chain; Sul, sulfonate side chain; Pho, phosphonate-containing side chain; Orn, ornithine-like side chain; Dap, diaminopropionic acid-like side chain; Leu, leucine-like side chain; Phe, phenylalanine-like side chain; Hyd, hydroxy side chain; Dol, diol-containing side chain; Deg, diethylene glycol-containing side chain; Bio, biotin-containing terminal unit; PEG, diethylene glycol-containing terminal unit. Colours indicate the chemical nature of the side chains: orange, acidic; blue, basic; black, hydrophobic; green, polar neutral. Bold bonds define the inner rim of helical oligomers. **b**, AOF sequences synthesized, written left to right in the N- to C-terminal direction. In **4**, the unit D is a diacid, resulting in a symmetrical sequence with two C termini. **c**, Crystal structure of the dodecaamide **1b**, shown from side and top view. The right-handed conformation is shown. The crystal also contains the left-handed helix. The bond order and hydrogen atoms are omitted for clarity. **d**, Crystal structure of the selected Nanofitin C10 at 1.4 Å (Supplementary Fig. 2) shown at the same scale as the structure of **1b** in **c**. Residues highlighted in pink, including five aromatic residues, are at positions randomized in the selection process. The coiled-coil extension was omitted for clarity. **e**, BLI sensorgrams of *P*-**1a** (blue curves) and *M*-**1a** (red curves) used as ligands on SA biosensors, against Nanofitin C10 (analyte) in a serial dilution from 250 nM to 62.5 nM in phosphate-buffered saline with 0.05% Tween 20 (PBST) revealing a dissociation constant (*K*_D_) of 92 nM for *P*-**1a**•C10. **f**, Circular dichroism (CD) spectra of C10 recorded in ten steps heating the sample from 5 °C to 95 °C (top). Molar ellipticity (*θ*) recorded at 222 nm heating from 5 °C to 95 °C, then cooling back to 5 °C, showing complete CD signal recovery (bottom). Source data

Developing a supramolecular synthon that combines a protein and a synthetic molecule entailed a judicious choice of both components. Synthetic oligomers that fold—foldamers—provide a variety of medium-sized scaffolds many of which have shown potential to interact with proteins^14,15^. In particular, aromatic oligoamide foldamers (AOFs) such as the oligoamides of 8-amino-2-quinoline carboxylic acid Q^*Xxx*^ (Fig. 1a) adopt particularly stable and predictable conformations and can be tailored to reach sizes that match those of small proteins^16^. For example, a simple octamer of Q^Leu^ remains helically folded at 120 °C in dimethyl sulfoxide^17^. The surface of AOFs can be decorated with biogenic-like side chains,^18^ and their main chain can be programmed to form helices of variable diameter as well as sheets^19,20^. Automated solid-phase synthesis (SPS) enables direct access to sequences in the 5–10-kDa range^21^. In principle, these combined features make AOFs suitable candidates for recognizing large surface areas of proteins. Efforts have until now focused on AOF design and screening, that is, on the identification of foldamers that bind to a biological protein target by varying helical^22^ or rod-like^23,24^ foldamer main chain and side chain components. However, structurally characterized complexes between proteins and helical AOFs are poorly stable (dissociation constant *K*_D_ in the micromolar range)^25^ or involve flexible linkers proscribed for the current purpose^26^. They thus do not constitute reliable supramolecular synthons. By contrast, the approach followed here consisted in identifying a protein that binds a given foldamer from a large library of protein sequence variants, leading to a protein–foldamer complex in which both components are artificial.

To identify a protein that would tightly bind a foldamer, we turned to display selection methods, which brought their own challenges. Antibodies and nanobodies are typical proteins amenable to display selection. They have flexible loops that can create cavities, and successful examples of display selection of nanobodies against small molecules have been reported^7,27^. However, their complex structure, relatively large size and moderate stability are not ideal to serve as supramolecular synthons. Smaller proteins based on non-immunoglobulin scaffolds would fare better in this respect. Many such proteins have been used in display selection to bind to other protein targets^28,29^. Yet examples of display selection against small synthetic molecules are rare; they typically involve loop binding regions rather than flat interfaces, and the structures of the complexes remain unknown^30,31^. We opted for the selection of Nanofitins^32,33^, which are based on the scaffold of Sac7d, a 66-residue cysteine-free protein stable over a broad range of temperature and pH^34^. Sac7d naturally binds to dsDNA, whose size is similar to that of (Q^*Xxx*^)_*n*_ helices. Binding is mediated by the slightly concave surface of an incomplete five-stranded β-barrel within which up to 14 residues have been randomly mutated for display selection. At their C-terminus, an α-helix caps the β-barrel and points away from the binding interface, thus providing a handle for fusion with other proteins. Furthermore, Nanofitin–protein complexes are amenable to X-ray crystallographic analysis^35–37^.

## Selection of a foldamer-binding protein

Biotinylated AOF **1a** (Fig. 1b), a Q^*Xxx*^ dodecaamide five-turn helix, was chosen as the selection target because its chemically diverse side chain decoration potentially offers a variety of protein-binding epitopes, as highlighted by the X-ray crystallographic structure of its analogue **1b** (Fig. 1c)^38^. Furthermore, the protein-binding capabilities of **1a** have been demonstrated in yeast lysate pull-down assays^38^. Nanofitin selection was performed in four rounds of ribosome display, as previously described^32^, using biotinylated **1a** as a bait. The enrichment of naive libraries towards binders was monitored by enzyme-linked immunosorbent assay (ELISA) using the DNA pools collected after each round (Supplementary Fig. 1). Subsequent cloning and sequencing led to the identification of clone C10. Foldamer **1a** exists as a mixture of right-handed (*P*) and left-handed (*M*) diastereomeric helical conformers that do not interconvert and in which the chiral biotin has no effect on the proportion of *P* and *M* helices, which are thus present in identical amounts. The diastereoisomers can be separated by reversed-phase high-performance liquid chromatography (RP-HPLC) on a chiral stationary phase^38^. C10 was recombinantly expressed and its affinity for *P*-**1a** and *M*-**1a** was assessed in parallel biolayer interferometry (BLI) assays. C10 was found to bind to *P*-**1a** with a *K*_D_ of 91.7 nM while no binding to *M*-**1a** was detected, hinting at a structurally well-defined interaction (Fig. 1e). We thus synthesized analogue **1c** whose N-terminal chiral C* camphanyl group quantitatively biases helix handedness to the *P* conformation^39^. Initial attempts to crystallize C10 alone or in complex with **1b** or **1c** were unsuccessful. Yet by C-terminal fusion of the coiled-coil talin dimerization domain^40^ (PDB 2QDQ) to C10, a high-resolution 1.4-Å X-ray crystallographic structure was obtained showing that the C10 apo-protein adopts the same tertiary fold as wild-type parent Sac7d (Fig. 1d, Supplementary Fig. 2 and Supplementary Table 1). The structure also showed that aromatic residues selected at randomly mutated positions (H22, F24, H26, Y33, H42) promote C10 dimerization in the solid state. Weak dimerization was also suspected in solution at concentrations required for NMR characterization (see below) and observed during size exclusion chromatography (SEC). Variable-temperature circular dichroism (CD) measurements highlighted the high thermostability of C10, showing a decrease of CD band intensity but no melting transition between 5 °C and 95 °C as well as complete CD signal recovery upon cooling (Fig. 1f), which boded well for its use in a supramolecular synthon.

## NMR complex structure elucidation

A partial assignment of the NMR spectra of [^13^C,^15^N]-labelled C10 was performed (Supplementary Fig. 3). The backbone amide signals of some residues were broadened and a few were missing. These were primarily residues from the region randomized during display selection. The broadening of these signals suggests that some aggregation involving this region of the protein surface takes place at micromolar concentrations (Supplementary Fig. 3). By contrast, the spectra of solutions of the **1c•**C10 complex were sharp and its structure could be determined, allowing for an assessment of the nature of the binding mode in solution. Compared with usual ligands in protein–ligand complexes, the large size of the foldamer ligand necessitated the use of a modified structure determination process (see Methods for details). To assist further in identifying protein, foldamer and intermolecular cross-peaks, we used [^13^C,^15^N]-labelled C10 combined with unlabelled foldamer **1c** at natural isotope abundance. This isotope labelling scheme allowed for the collection of edited or filtered NMR spectra so that signals of the protein only, of the foldamer only or of both the foldamer and protein are observed (Supplementary Figs. 4–6 and Supplementary Table 2). The resulting ensemble of 20 structures was calculated using restrained molecular dynamics in AMBER with 114 dihedral angle restraints and 737 distance restraints distributed throughout the complex (Supplementary Fig. 7 and Supplementary Tables 3 and 4).

**Fig. 2:**
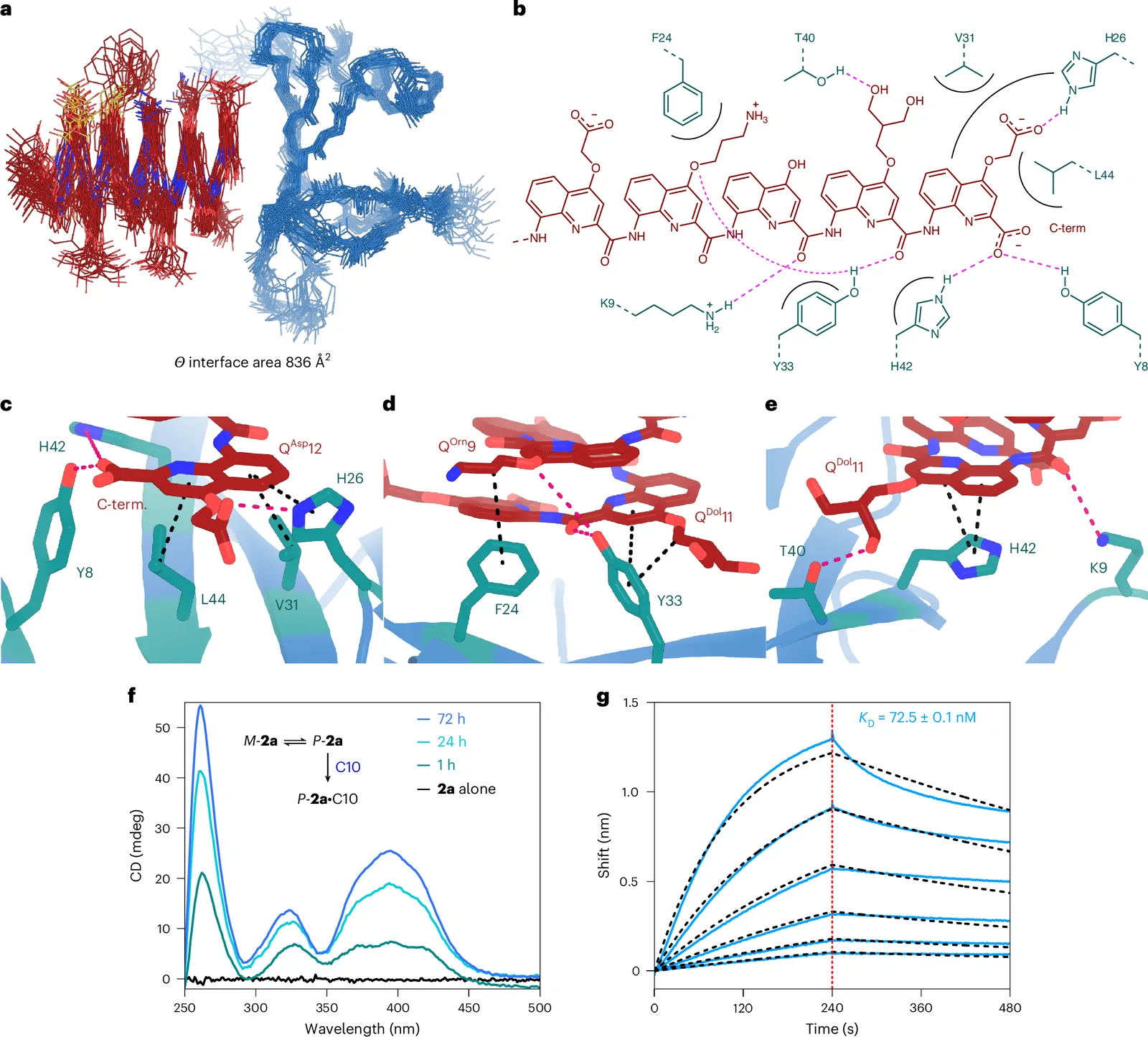
Nuclear magnetic resonance (NMR) structure elucidation reveals the protein–foldamer complex binding epitope. **a**, NMR structure of the **1c•**C10 complex showing the aligned ensemble of 20 models. Structure statistics can be found in Supplementary Table 3. **b**, Schematic diagram of intermolecular contacts between C10 and the last four quinoline monomers of **1c** in the binding epitope identified with protein–ligand interaction profiler^50^. Hydrogen bonds are shown as pink dashed lines and hydrophobic contacts as black solid lines. **c**–**e**, Three close-up structure views from different directions of important foldamer–protein contacts with annotated foldamer units and protein side chains. Hydrogen bonds are shown as pink dashed lines, and hydrophobic contacts as black dashed lines. **f**, Time course of CD spectrum of pentameric sequence **2a** after addition of C10 at 30 µM, tris-buffered saline (TBS) pH 7.4, 25 °C. Note that the biotin moiety of **2a** does not bias foldamer helix handedness as seen in the flat curve for **2a** alone. **g**, BLI sensorgrams (blue solid lines) of C10 in solution and **2a** loaded on SA-biosensors and global fitting using a 1:1 binding model (black dashed lines). Kinetic experiments were performed in PBST at pH 7.4 with a serial dilution of C10 from 500 nM to 15.6 nM. Source data

The NMR structure of the **1c**•C10 complex revealed the architecture of the 1:1 assembly (Fig. 2a). The C-terminal aromatic cross-section of **1c** covers a large area of the protein β-sheet region (~830 Å^2^, Fig. 2a), where most of the residues that were randomized during the selection process are located, while the N-terminus of the helix protrudes away from the protein. The interaction between the extended aromatic C-terminal region of **1c** with the flat C10 surface is somewhat reminiscent of G-quadruplex DNA–protein structures^41^. The upright binding mode of the foldamer helix on C10 differs from side-on helix orientations previously observed in more labile aromatic helical foldamer–protein complexes^25^. In atomic detail, the **1c•**C10 binding interface primarily consists of (1) face-to-face *π*-stacking between the Q^Dol^11 quinoline ring and H42 and T-shaped *π–π* interaction between Q^Asp^12 and H26; (2) hydrophobic contacts between the two solvent-exposed terminal quinoline rings and residues V31 and L44, between the side chain of Q^Dol^11 and Y33 and between Q^Orn^9 and F24; (3) hydrogen bonding of Y33 to the main chain Q^Dol^11–Q^Asp^12 amide carbonyl and between T40 and the side chain of Q^Dol^11; and (4) charge-reinforced hydrogen bonding of both Y8 and H42 to the C-terminal carboxylate of the foldamer and of H26 to the side chain carboxylate of Q^Asp^12 (Fig. 2b–d). Interactions thus mainly involve residues located at the positions randomized during selection with the notable exception of Y8 and K9. The ensemble of 20 NMR structures determined for the **1c**•C10 complex shows limited variation in the relative position of the foldamer and protein, with an overall all-heavy atom RMSD of 1.7 ± 0.2 Å (Fig. 2a and Supplementary Table 3). Furthermore, molecular dynamics simulations of the complex confirm that the foldamer position and orientation on the C10 surface are essentially stable (Supplementary Video 1). The simulations also highlight the structural integrity of the complex in terms of residue fluctuations and hydrogen bond frequencies (Supplementary Figs. 8 and 9). Taken together, the solution structure data of **1c•**C10 validate the potential of this complex as a supramolecular synthon. Furthermore, the structure confirmed the location of the α-helix of C10 away from the foldamer-binding region and thus its potential use as a handle to fuse C10 with other proteins without altering foldamer binding.

## Foldamer helix truncation

Large parts of the foldamer are not in contact with C10 in the complex, suggesting that a truncated version of **1** might bind equally well. We thus prepared pentaamide **2a** which comprises the five C-terminal units of **1**. For such short sequences, the equilibrium between *P* and *M* helices is expected to take place over the course of hours^42^. Indeed, upon mixing **2a** and C10, CD bands above 300 nm where only the foldamer absorbs were found to slowly emerge (Fig. 2f), indicating foldamer helix handedness bias upon binding to the protein. The emergence of the CD band occurred faster at 60 °C owing to enhanced helix dynamics, confirming that binding still takes place at such high temperatures. The positive band at 380 nm confirmed that the *P* helix is the favoured binding conformation^39^. BLI measurements with biotinylated **2a** confirmed that it binds as well as *P*-**1a**. Note that helix handedness bias of **2a** is expected to remain marginal during the few minutes required for BLI measurements. We also prepared shorter foldamers having the last four, three or only two C-terminal units of **1a**. BLI and CD assays showed that strong binding was retained with four residues but worsened below that number (Supplementary Fig. 10). Altogether, these results support that foldamer–C10 interactions are limited to those revealed by the NMR structure and that C10 and a pentaamide foldamer helix provide a robust supramolecular synthon for the elaboration of more complex architectures with controlled spatial organization.

## Binding multiple proteins on the same foldamer

**Fig. 3:**
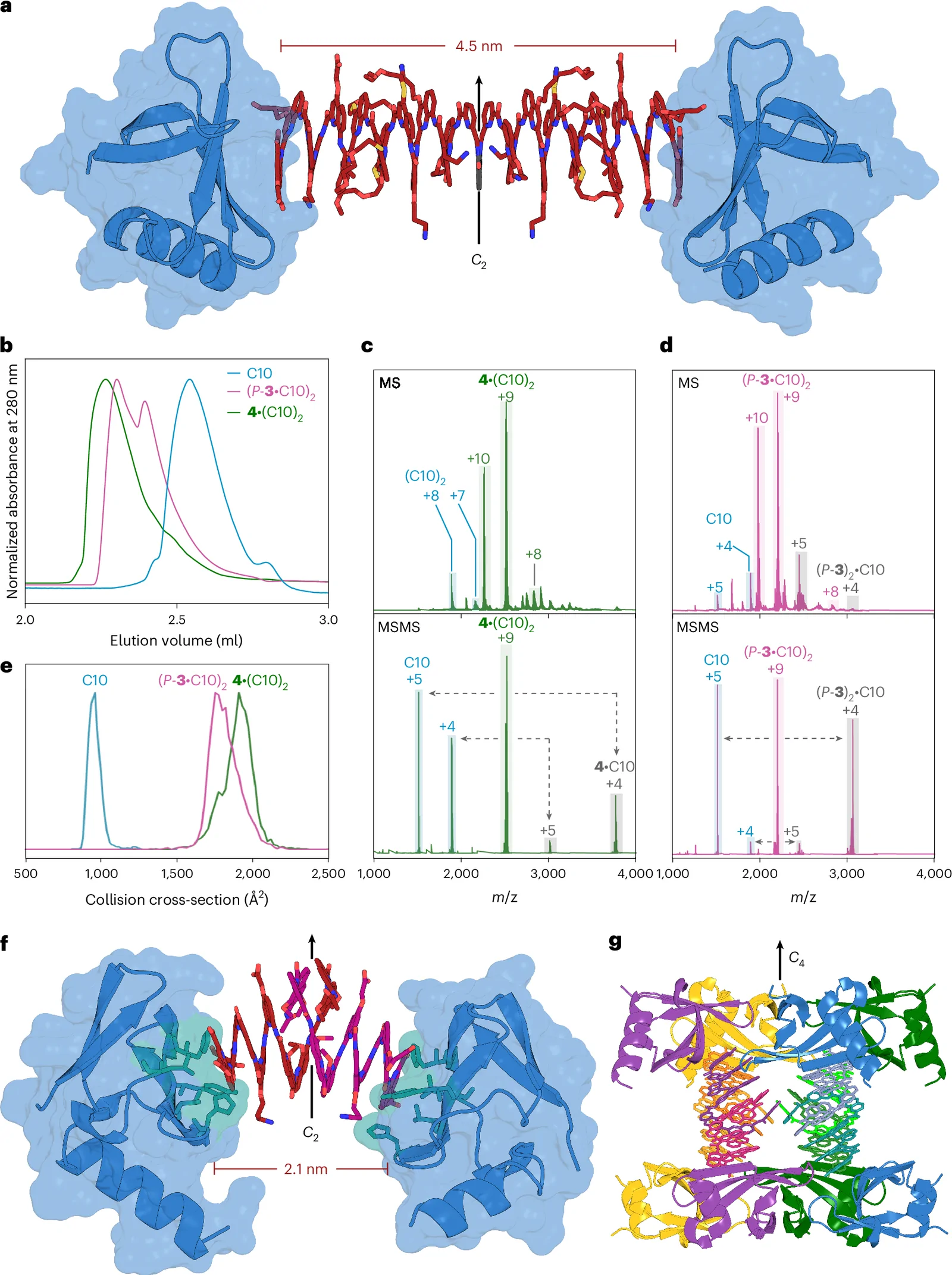
Binding multiple proteins on the same foldamer. **a**, Energy-minimized model of the *C*_2_-symmetrical **4•**(C10)_2_ complex. **b**, Size-exclusion chromatograms showing the formation of foldamer protein complexes as indicated by the lower elution volume for **4•**(C10)_2_ and (*P*-**3•**C10)_2_ compared with the protein alone. The split peak for (*P*-**3•**C10)_2_ was assigned to the presence of some (*P*-**3**)_2_•C10. **c,d**, Native mass spectrometry (MS) spectra of 1:2 and 1:1 mixtures of **4**/C10 and *P*-**3**/C10, respectively, at 100 µM in 200 mM ammonium acetate pH 6.8 (top), detecting the formation of **4•**(C10)_2_ and (*P*-**3•**C10)_2_. The complexes were subjected to MSMS and collisional activation (bottom). Dashed arrows in the MSMS spectra indicate the dissociation produced from the m/z-selected precursor ions. **e**, Collision cross-section in the gas phase (^TW^CCS_N2→He_) of the C10^+5^, **4•**(C10)_2_^+9^ and (*P*-**3•**C10)_2_^+9^ ions measured by ion mobility mass spectrometry. **f**, Asymmetric unit of the crystal structure of (*P*-**3•**C10)_2_ at 1.84 Å resolution containing two protein chains bridged by the foldamer duplex (*P*-**3**)_2_. A pseudo (non-crystallographic) *C*_2_ axis is indicated. **g**, Two different views of the *C*_4_-symmetrical higher order assembly observed in the (*P*-**3•**C10)_2_ X-ray crystallographic structure. The cyclic 8+8 aggregate comprised of four (*P*-**3•**C10)_2_ complexes is stabilized by inter-duplex *π-π* contacts between Q_OMe_ units and side chain-mediated charge-reinforced hydrogen bonds. Source data

To test the generality and robustness of the supramolecular synthon upon modifications of the foldamer remote from the binding site, variants **3** and **4** were designed and synthesized (Fig. 1b), each presenting two binding epitopes through different modes of symmetric multimerization. Variant **4** consists of a long *C*_2_-symmetrical 31-mer single helix with two identical halves linked at their N termini by a central diacid unit D. Each half of **4** thus possesses a C-terminal C10-binding pentaamide elongated at its N terminus by a B* chiral unit, a number of hydrophilic Q^Orn^ and Q^Deg^ residues and a P residue, all assembled during SPS (Supplementary Fig. 11). The B* chiral control unit quantitatively biases helix handedness to the *P* conformation^43^, and the benzylic amine of the P unit facilitates the post-SPS assembly of **4** by fragment condensation on the diacid of unit D activated as a bis-*N*-hydroxysuccinimide ester^25^. The complexation of **4** with C10 was analysed by SEC. It resulted in a decrease of the retention time, indicating the formation of a presumably dumbbell-shaped ternary complex (Fig. 3a,b). The complex was also analysed in the gas phase using native nano-electrospray ionization mass spectrometry (nESI-MS). The **4•**(C10)_2_ complex was found to be the dominant species, and its fragmentation into C10+**4•**C10 could be monitored by tandem mass spectrometry (MSMS, Fig. 3c). Analogues of **4** having longer or shorter helices are thus expected to rigidly hold in space two C10 molecules (and any protein fused to C10) at a distance and twist angle precisely defined by the number of AOF residues.

Variant **3** uses an N-terminal extension of the pentaamide binding epitope with four 7-amino-8-methoxy quinoline carboxylic acid Q_OMe_ residues that code for a wider aromatic helix diameter, which in turn promotes dimerization into stable antiparallel double helices in water^44^. The resulting duplex of sequence **3** binds two copies of C10, and the 2+2 quaternary complex can also be traced by SEC, showing an elution volume smaller than that of the protein or foldamer alone, yet larger than that of **4•**(C10)_2_, reflecting the effect of foldamer length on elution time (Fig. 3b). In the gas phase, the (*P-***3**•C10)_2_ complex was also observed by native nESI-MS (Fig. 3d) to be the dominant species. Its main fragmentation in MSMS was through a loss of a C10 unit and the formation of (*P-***3**)_2_•C10, and not through the disruption of the (*P-***3**)_2_ double helix highlighting its robustness. Furthermore, ion mobility measurements gave access to the collision cross-sections of C10, (*P-***3**•C10)_2_ and **4•**(C10)_2_ showing a trend consistent with the SEC data (Fig. 3e). Note that helix handedness control was not implemented in **3** and that, given its length, its helical conformation is kinetically locked (no CD band emerges upon adding C10 to **3**). In solutions of racemic (**3**)_2_ duplexes, only the *P* helices thus bind to C10. We successfully crystallized and solved the structure of the (*P-***3**•C10)_2_ complex, thus resolving the racemic (**3**)_2_ duplexes by crystallization. The structure at 1.84 Å resolution confirmed the expected dumbbell geometry, with two C10 molecules spatially separated upon binding to the opposite sides of double helical (**3**)_2_ (Fig. 3f, Supplementary Video 2 and Supplementary Table 1). The asymmetric unit (ASU) contains two independent foldamer–C10 interfaces which show highly conserved interactions essentially identical to those of the NMR structure (Supplementary Figs. 12 and 13, Supplementary Table 5), highlighting again the robustness of the supramolecular synthon.

The X-ray crystallographic structure of C10 bound to the foldamer was also compared with that of the apo-C10, showing that the main chains are essentially superimposable, an illustration of the robustness of the Nanofitin scaffold, while surface side chains, including those involved in foldamer recognition, may have different orientations (Supplementary Fig. 12). Future developments may exploit the use of longer helical segments to adjust protein–protein distance–foldamer length spontaneously doubles upon duplex formation, and the fact that heteromeric foldamer double helices form selectively when complementary strands are used^44^.

In the solid state, the (*P-***3**•C10)_2_ complexes form a higher-order tetrameric [(*P-***3**•C10)_2_]_4_ assembly organized in a fourfold cyclic arrangement (Fig. 3g and Supplementary Video 3). The assembly is held together by face-to-face contacts between Q_OMe_ residues belonging to different duplexes and an extensive array of inter-duplex charge-reinforced hydrogen bonds (Supplementary Fig. 14). In solution, the 8+8 aggregate was not detected by electron microscopy, and SEC retention volume shifts seem to be too small for an ~80-kDa object. It was also not visible in native nESI-MS spectra measured from micromolar solutions (Fig. 3d). We concluded that the assembly forms only at the mM concentration of the crystallization drops and possibly represents a minor component in solution, even at these higher concentrations, that would only be stabilized in the solid state. It nevertheless represents a starting point for designing stable foldamer assemblies able to bind multiple—here eight—proteins.

## Binding multiple foldamers on a protein dimer

We next investigated modifications on C10 to explore how to spatially organize the supramolecular synthon via multimerization of the protein. Coil coiling of α-helices is arguably the best understood aggregation pattern and the easiest to design^1,2,45^. Implementation of a coiled coil on C10 is straightforward, thanks to its C-terminal α-helix that points away from the foldamer-binding region. We opted again for a simple fusion with the antiparallel coiled-coil domain of talin mentioned above, albeit with some design improvements. In the first design, two consecutive glycine residues (G66&67) present at the junction between C10 and the coiled coil were responsible for a local disruption of α-helix that kinked the structure (Supplementary Fig. 1). In this second design, these two glycine residues were replaced by alanine residues to better promote α-helicity. Crystallization efforts with this coil-extended C10 construct (C10CE) in complex with the initial target sequence, for example, **1b** or **1c**, repeatedly yielded crystals of the foldamer alone, presumably owing to its lower solubility. Therefore, pentamer **2b** (Fig. 1b) was synthesized, carrying a solubilizing PEG termination as well as two phosphonate-containing Q^Pho^ residues that were expected to improve solubility with respect to the original carboxylate Q^Asp^ units. Native nESI-MS showed the formation of the **2b*•***C10CE complex along with the (**2b*•***C10CE)_2_ dimer. The proportions also seen in the spectrum of C10CE alone suggested some lability of the coiled-coil dimerization in the gas phase. The (**2b*•***C10CE)_2_ dimer was found to fragment via coiled-coil dissociation (Supplementary Figs. 15, 16).

**Fig. 4:**
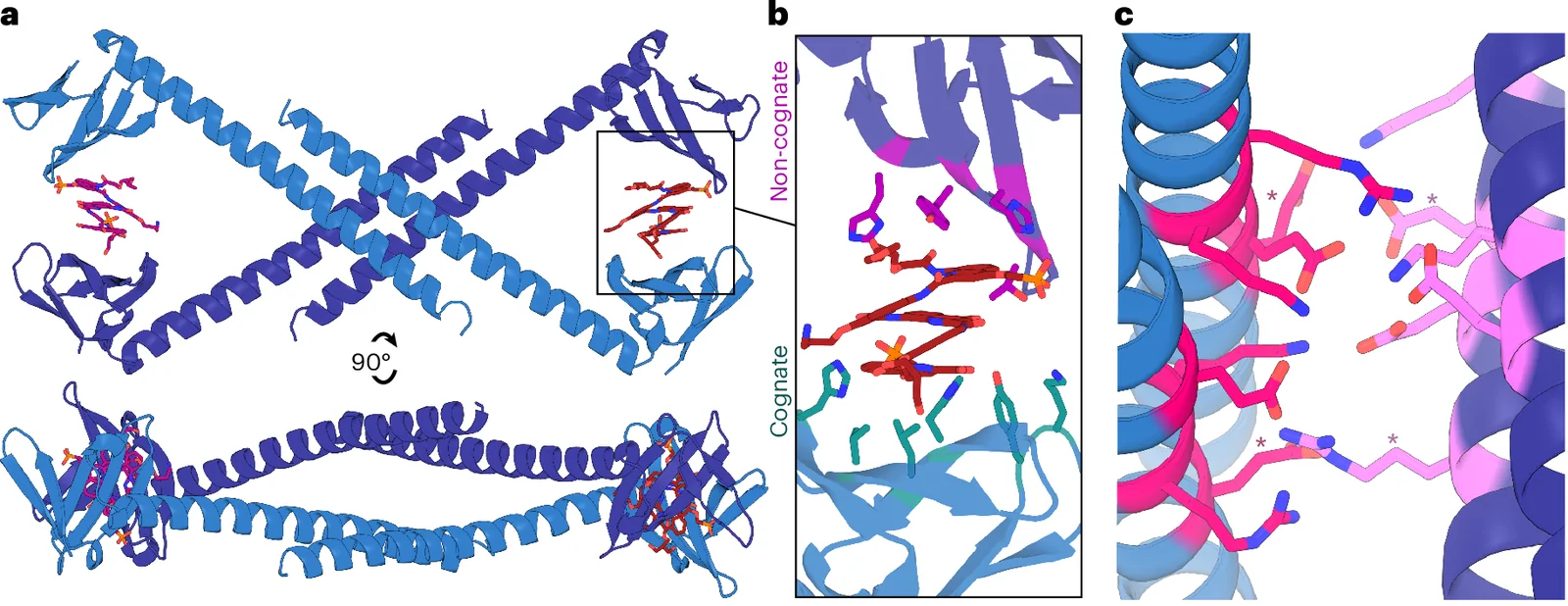
Binding multiple foldamers by protein dimerization. **a**, Crystal structure of the coil-extended C10 (C10CE) in complex with pentaamide **2b**. The ASU is shown from two perspectives, illustrating the cyclic arrangement of two foldamers and four C10CE proteins in the solid state. **b**, Detailed view of the foldamer packed between two C10 molecules, with the cognate-binding epitope highlighted in teal on one protein and non-cognate interactions in purple on the opposite chain. **c**, Highlights of Glu, Lys and Arg inter-coiled-coil interactions, connecting two protein dimers. Residues marked with a star were reconstituted from the crystal structure and energy minimized.

Diffraction quality crystals of the C10CE in complex with **2b** were eventually obtained, and the X-ray crystallographic structure was elucidated (Fig. 4, Supplementary Fig. 17 and Supplementary Table 1). In the ASU, two coiled-coil protein dimers are in complex with two molecules of **2b** through their C-terminal cross-section, providing further evidence that this robust, cognate interaction is highly conserved (Fig. 4b, Supplementary Fig. 13 and Supplementary Table 5). In addition, the two C10 domains establish contacts with the N-terminal cross-section of the **2b** helices (Fig. 4b). This interaction only concerns the N-terminal quinoline ring and its short PEG chain, which involve hydrophobic contacts with Y33, H22 and T40 and a charge-reinforced hydrogen bond between H42 and a phosphonate side chain. We presume that they are weaker than the cognate foldamer–C10 recognition and only occur at the mM concentration of crystallization drops at substoichiometric amounts of foldamer. There is thus little risk that such non-cognate interactions would compete with the 91.7-nM cognate C10–foldamer recognition. The 4+2 cyclic assembly—four proteins and two foldamers—mediated by the non-cognate interactions was not detected by native nESI-MS from micromolar solutions (Supplementary Fig. 15). It is possibly a crystallization artefact—it crystallizes although it represents a minor component in solution. It may also be the result of a substoichiometric amount of foldamer. Nevertheless, the outcome is an X-shaped, figure-of-8, discrete macrocyclic assembly between two foldamers and two C10CE coiled-coil dimers (Fig. 4a, Supplementary Video 4). At the centre of the cyclic structure, favourable contacts between the two coiled coils are mediated by hydrogen bonds between multiple Glu, Lys and Arg residues that may be amenable to optimization (Fig. 4c). Altogether, the structure validates, and goes beyond, the initial design principles, and it suggests directions for further developments.

## Binding multiple proteins with multiple foldamers in one system

**Fig. 5:**
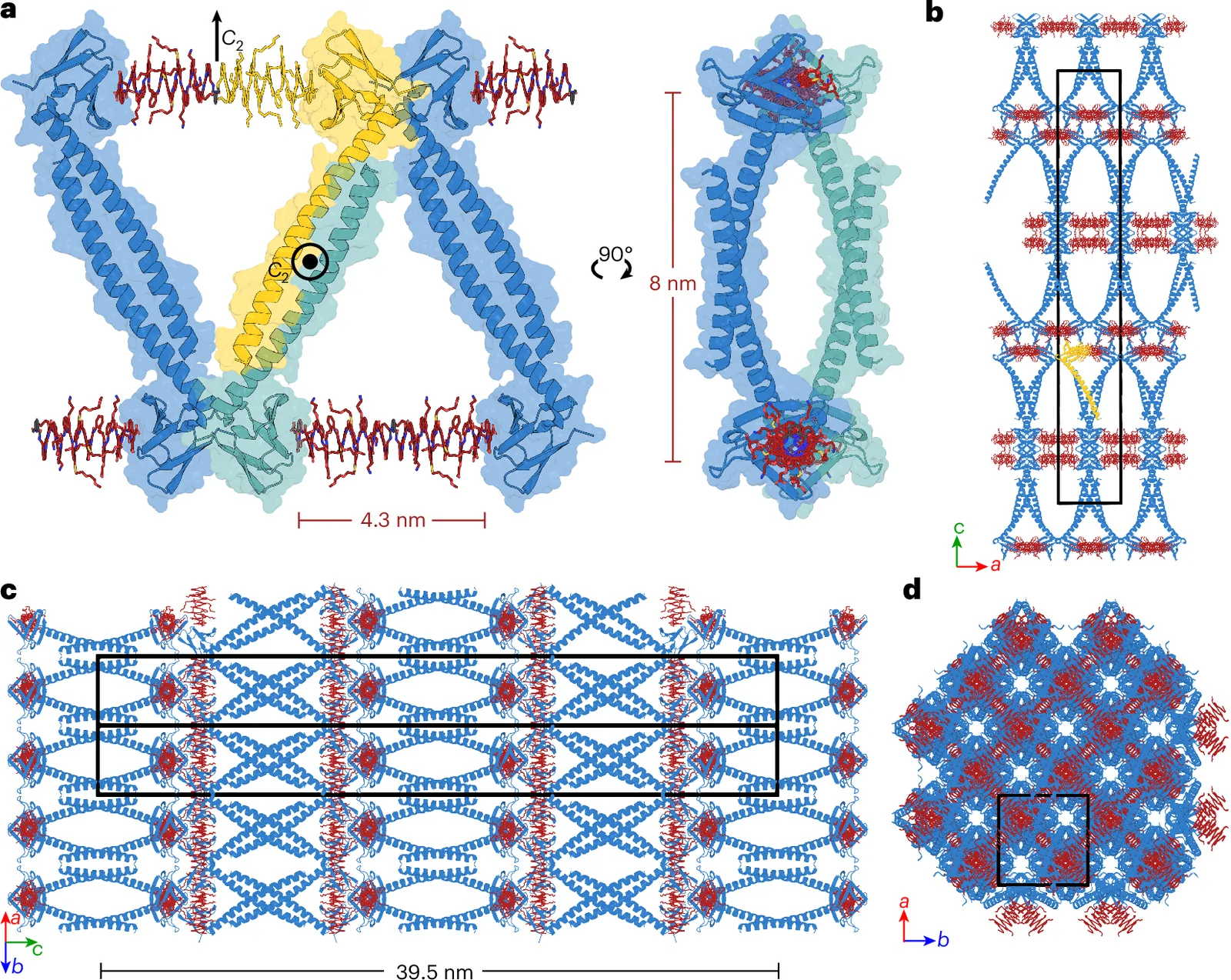
Crystal structure of the coiled-coil-mediated C10CE dimer in complex with the *C*_2_ symmetrical foldamer 4 (4•(C10CE)_2_) at 2.53 Å. **a**, Continuous ribbon built of protein dimers connected to the *C*_2_-symmetrical foldamer **4**. The ASU (highlighted in gold) contains one protein monomer and one-half of **4**. The imposed local *C*_2_ axes of the complex coincide with crystallographic symmetry axes, giving rise to a highly ordered assembly in the space group I4_1_22. **b**–**d**, Orthoscopic views of the crystal lattice along different directions: along (010) (**b**), along (110) (**c**) and along (001) (**d**). In all cases, the unit cell is shown in black. Proteins are shown as blue ribbons and foldamers as red sticks.

Based on the established principles of enforced multimerization around the foldamer–C10 supramolecular synthon, we lastly combined the rigid homochiral divalent single helix of **4** with the head-to-tail coiled-coil-mediated dimer of C10CE. Crystallization trials quickly yielded diffraction quality crystals and revealed a structure of high symmetry, with the ASU containing one protein monomer and half of the *C*_2_-symmetrical **4** (Fig. 5, Supplementary Table 1). This structure provided yet one more illustration of the conservation of C10–foldamer interactions (Supplementary Fig. 1–3, Supplementary Table 5). Through application of the symmetry operations, a 1D polymeric zig-zag-like network that extends in a sort of flat ribbon is built (Fig. 5a). The ribbon consists mainly of cognate foldamer–C10-binding interfaces and the α-helical coiled coiling included in the design, along with C10–C10 contacts associated with crystal packing. The coincidence of the symmetry elements of the building blocks with the crystallographic rotational axes can be interpreted as an enforcement of global symmetry by predisposed local symmetries^46^. Here the *C*_2_ symmetry axis of the foldamer and the *C*_2_ symmetry axis of the C10 coiled-coil dimer are perpendicular, hence the formation of a flat assembly, but they do not intersect, giving rise to a polymeric structure rather than a macrocycle. This assembly is the direct outcome of the distance and angular twist between the protein-binding epitopes of **4** encoded in the number of Q^*Xxx*^ residues and of the distance and angular twist between the foldamer-binding epitopes of the C10 coiled-coil dimer encoded by the length of the α-helices. In the gas phase, the 1D polymer was not observed by native nESI-MS (Supplementary Fig. 16).

While the crystal lattice appears to be tightly packed when viewed down its *c*-axis (Fig. 5d), it is in fact highly porous, with the foldamer spacing the proteins ~4.3 nm apart, while the coiled-coil domain keeps the foldamers ~8 nm apart (Fig. 5a–c). Computational analysis of the pores showed that the resulting channels in *a* and *b* directions reach a bottleneck radius of 15.5 Å and that the largest cavities can accommodate theoretical spheres with a diameter of ~5 nm (Supplementary Fig. 18)^47^, a value comparable to that of other highly porous frameworks^11^. A typical way to assess solvent content in protein crystal structures is through the Matthews coefficient, which describes the unit cell volume per protein mass (but does not tell anything about pore size). The mean Matthews coefficient of Protein Data Bank (PDB) protein structures is 2.67 Å^3^ Da^−^^1^ (ref. ^48^) to be compared with 3.51 Å^3^ Da^−^^1^ for the porous lattice mentioned above^11^ and 5.13 Å^3^ Da^−^^1^ for the structure of **4**•(C10CE)_2_. This contrasts with the low porosity of the structure obtained with the pentaamide helix **2b**•(C10CE)_2_ (Fig. 4), which has a calculated maximum bottleneck size of 8.6 Å, and reflects the benefit of the space between proteins imposed by **4**. Considering that the length of the foldamer is freely adjustable (within synthetic limits), spacing within such lattices is, in principle, tunable, highlighting the relevance of protein–synthetic foldamer assemblies in the context of porous solid design.

In summary, in contrast to smaller molecules or peptides, aromatic foldamers constitute large and rigid-enough synthetic molecules to allow for successful display selection of protein binders and the generation of a well-defined and robust binding interface. It appears that the foldamer aromatic cross-section acted as a hot spot—one such protein–foldamer contact had been observed before with carbonic anhydrase II (ref. ^49^), and this feature may be exploited in future studies to target proteins with flat surfaces. The identification of C10 also suggests that targets other than aromatic foldamers may be amenable to the selection of protein binders, to bridge further the synthetic and protein chemical spaces. In turn, both the foldamer and protein can be further designed without altering their binding interactions so as to produce discrete or extended aggregates and lattices combining both biogenic and synthetic components. Extensions of this work will take advantage of the inherent modularity and synthetic accessibility of foldamers, of the diversity of protein scaffolds amenable to display selection such as the Nanofitin described in our study^29^ and of the advanced computational approaches to design protein structures and interaction^1–3^. These extensions include the design, either structure-guided or using ab initio protein design tools, of foldamer–protein nanocages and porous 3D lattices in which the foldamers may bring functional groups not available to proteins. They also included the use of foldamers to bring together or, on the contrary, to keep at a well-defined distance, naturally occurring proteins that have been tagged with foldamer-binding domains to alter their biological functions.

## Methods

Detailed methods are provided in the Supplementary Information.

### Foldamer synthesis

All foldamers were obtained by manual or automated solid-phase-supported synthesis from Fmoc-amino acid monomers—Fmoc-Q^*Xxx*^-OH, Fmoc-Q_OMe_-OH, Fmoc-P-OH and Fmoc-B*-OH—bearing suitable acid-labile side chain-protecting groups, for example, Boc for Q^Orn^, Q^Dap^ and Q_OMe_ (Fig. 1a). The monomers used in this study are not commercially available and were synthesized following well-established protocols. In general, the carboxylic acid function of the monomers was activated as its acid chloride by applying in situ Appel’s conditions in the presence of triphenylphosphine and trichloroacetonitrile. Coupling reactions to the free amine of an oligomer were performed in the presence of 2,4,6-collidine as a base. Subsequent deprotection of the main chain amine, that is, Fmoc removal, was carried out with 2% DBU (1,8-diazabicyclo[5.4.0]undec-7-ene) in *N*-methyl-2-pyrrolidone. Removal of the silyl ether protection of Q^Dol^ was carried out on-resin with 1 M tetrabutylammonium fluoride in THF. Cleavage from the solid support was performed using a mixture of TFA, triisopropylsilane and H_2_O (95:2.5:2.5; v/v/v) for Wang resin, thereby leading to the deprotection of the other side chains, or with 1% TFA in dichloromethane for SASRIN resin, leaving the other side chain-protecting groups intact. Fragment condensation en route to the *C*_2_-symmetrical helical foldamer **4** was performed in DMF with the di-*N*-hydroxysuccinimide active ester of pyridine-2,6-dicarboxylic acid D and diisopropylethyamine as a base. All foldamer sequences were purified by RP-HPLC.

### Nanofitin selection

The combinatorial library of Nanofitins for ribosome display was assembled by two successive overlapping PCRs from degenerate oligonucleotides encoding NNS triplets (N=A, C, T or G and S=C or G). Finally, a PCR step added the 5′- and 3′-flanking regions necessary for ribosome display. The PCR-amplified library was transcribed, and the selection was performed in four rounds at 4 °C against 15 pmol of the immobilized biotinylated foldamer **1a**. Amplified DNA material from the last round was cloned between BamHI and HindIII restriction sites of a plasmid derived from pQE-30 (Qiagen), and the ligation mixture was transformed into *Escherichia coli* DH5α LacIq strains (Invitrogen). Expression of the Nanofitin clones was induced by the addition of isopropyl β-D-1-thiogalactopyranoside (IPTG). The selection target **1a** was immobilized on streptavidin (SA) MaxiSorp plates, and crude *E. coli* extracts were applied to wells with and without immobilized antigen for 1 h. Detection was carried out by the addition of an RGS–His antibody HRP conjugate (Qiagen), followed by the addition of *o*-phenylenediamine dihydrochloride substrate (Sigma-Aldrich) solution. Absorbance at 450 nm was measured using a Varioskan enzyme-linked immunosorbent assay plate reader (Thermo Scientific), revealing Nanofitin C10 as a binder with a strong signal-to-background ratio.

### Nanofitin expression and purification

Nanofitin C10 and the coil-extended versions were expressed recombinantly in *E. coli* BL21(DE3) as N-terminally His-tagged or MBP-His-tagged fusion proteins. Plasmids (pET-28a(+), pMAL-c5E) were purchased ready to use as lyophilized solids (GenScript). For unlabelled proteins, cultures were grown in LB. For the expression of the [^13^C,^15^N]-isotope-labelled C10, M9 minimal medium supplemented with d-glucose-^13^C_6_, ^15^NH_4_Cl, and trace element solutions was used. Overexpression was induced with IPTG. Purification was carried out by Ni-NTA IMAC. Tag removal was achieved by cleavage with HRV-3C protease overnight. Final purification was carried out by SEC.

### Reporting summary

Further information on research design is available in the Nature Portfolio Reporting Summary linked to this article.

## Online content

Any methods, additional references, Nature Portfolio reporting summaries, source data, extended data, supplementary information, acknowledgements, peer review information; details of author contributions and competing interests; and statements of data and code availability are available at https://doi.org/10.1038/s41557-026-02222-6.

## Supplementary information

Supplementary Information Supplementary Sections 1–5, including Supplementary Figs. 1–18, Supplementary Tables 1–5, legends of Supplementary Videos 1–4 and Supplementary References.

Reporting Summary

Supplementary Video 1 Supplementary video—MD simulation of the 1c•C10 complex.

Supplementary Video 2 Supplementary video—animated view of the (P-3•C10)_2_ complex.

Supplementary Video 3 Supplementary video—animated view of the 8+8 assembly of four (P-3•C10)_2_ complexes.

Supplementary Video 4 Supplementary video—animated view of the cyclic X-shape assembly of two foldamers P-2b and two C10 coiled-coil dimers.

## Source data

Source Data Fig. 1 Statistical source data for Fig. 1e,f (BLI and CD raw data).

Source Data Fig. 2 Statistical source data for Fig. 2f,g (CD and BLI raw data).

Source Data Fig. 3 Statistical source data for Fig. 3b–e (SEC, native MS and collision cross-section).

## Data Availability

X-ray crystallographic structures have been deposited at the PDB under 9QDO, 9QT7, 9QOG and 9QNU. The ensemble of 20 structures calculated for the **1c**•C10 complex has been deposited at the PDB under accession 9T0O. The chemical shift assignments for the unbound C10 and **1c•**C10 complex were deposited in the BMRB (https://bmrb.io/) under accession numbers 53300 and 53317, respectively. The raw data that served to create the graphs in Figs. 1, 2 and 3 as well as BLI data have been deposited at Open Data LMU under https://data.ub.uni-muenchen.de/701/1/C10_BLI-raw_Data.zip (https://doi.org/10.5282/ubm/data.818). All other data are available in the article or the Supplementary Information. Source data are provided with this paper.

## References

[1] Regan, L. & DeGrado, W. F. Characterization of a helical protein designed from first principles. *Science* **241**, 976–978 (1988). 10.1126/science.3043666 3043666

[2] Thomson, A. R. et al. Computational design of water-soluble α-helical barrels. *Science* **346**, 485–488 (2014). 10.1126/science.1257452 25342807

[3] Hsia, Y. et al. Design of a hyperstable 60-subunit protein icosahedron. *Nature* **535**, 136–139 (2016). 10.1038/nature18010 PMC4945409 27309817

[4] Suzuki, Y. et al. Self-assembly of coherently dynamic, auxetic, two-dimensional protein crystals. *Nature* **533**, 369–373 (2016). 10.1038/nature17633 PMC4991361 27135928

[5] Lai, Y. T., Cascio, D. & Yeates, T. O. Structure of a 16-nm cage designed by using protein oligomers. *Science* **336**, 1129–1129 (2012). 10.1126/science.1219351 22654051

[6] Luo, Q., Hou, C., Bai, Y., Wang, R. & Liu, J. Protein assembly: versatile approaches to construct highly ordered nanostructures. *Chem. Rev.* **116**, 13571–13632 (2016). 10.1021/acs.chemrev.6b00228 27587089

[7] Shui, S., Buckley, S., Scheller, L. & Correia, B. E. Rational design of small-molecule responsive protein switches. *Protein Sci.* **32**, e4774 (2023). 10.1002/pro.4774 PMC10510469 37656809

[8] Soini, L., Leysen, S., Davis, J. & Ottmann, C. Molecular glues to stabilise protein–protein interactions. *Curr. Opin. Chem. Biol.* **69**, 102169 (2022). 10.1016/j.cbpa.2022.102169 35749929

[9] Liu, Z. et al. An overview of PROTACs: a promising drug discovery paradigm. *Mol. Biomed.* **3**, 46 (2022). 10.1186/s43556-022-00112-0 PMC9763089 36536188

[10] Troup, R. I., Fallan, C. & Baud, M. G. J. Current strategies for the design of PROTAC linkers: a critical review. *Explor. Target. Anti-tumor Ther.* **1**, 273–312 (2020). 10.37349/etat.2020.00018 PMC9400730 36046485

[11] Engilberge, S., Rennie, M. L., Dumont, E. & Crowley, P. B. Tuning protein frameworks via auxiliary supramolecular interactions. *ACS Nano* **13**, 10343–10350 (2019). 10.1021/acsnano.9b04115 31490058

[12] van Dun, S., Ottmann, C., Milroy, L. G. & Brunsveld, L. Supramolecular chemistry targeting proteins. *J. Am. Chem. Soc.* **139**, 13960–13968 (2017). 10.1021/jacs.7b01979 PMC5639466 28926241

[13] Mockler, N. M., Raston, C. L. & Crowley, P. B. Making and breaking supramolecular synthons for modular protein frameworks. *Chem. Eur. J.* **31**, e202500732 (2025). 10.1002/chem.202500732 PMC12089892 40178192

[14] Gopalakrishnan, R., Frolov, A. I., Knerr, L., Drury, W. J. III & Valeur, E. Therapeutic potential of foldamers: from chemical biology tools to drug candidates? *J. Med. Chem.* **59**, 9599–9621 (2016). 10.1021/acs.jmedchem.6b00376 27362955

[15] Pasco, M., Dolain, C. & Guichard, G. in *Comprehensive Supramolecular Chemistry**II* 89–125 (Elsevier, 2017).

[16] Wang, S., Sigl, J., Allmendinger, L., Maurizot, V. & Huc, I. Design of an abiotic unimolecular three-helix bundle. *Chem. Sci.* **16**, 1136–1146 (2025). 10.1039/d4sc07336c PMC11615733 39640026

[17] Jiang, H., Leger, J. M. & Huc, I. Aromatic delta-peptides. *J. Am. Chem. Soc.* **125**, 3448–3449 (2003). 10.1021/ja029887k 12643704

[18] Zwillinger, M. et al. Development of aromatic foldamer building blocks bearing multiple biogenic side chains. *J. Org. Chem.* **90**, 3043–3052 (2025). 10.1021/acs.joc.4c02900 39964779

[19] Gong, B. et al. Creating nanocavities of tunable sizes: hollow helices. *Proc. Natl Acad. Sci. USA* **99**, 11583–11588 (2002). 10.1073/pnas.162277099 PMC129312 12177422

[20] Atcher, J. et al. Large-amplitude conformational changes in self-assembled multi-stranded aromatic sheets. *Angew. Chem. Int. Ed.* **60**, 2574–2577 (2021). 10.1002/anie.202014670 PMC7898896 33156974

[21] Corvaglia, V., Sanchez, F., Menke, F. S., Douat, C. & Huc, I. Optimization and automation of helical aromatic oligoamide foldamer solid-phase synthesis. *Chem. Eur. J.* **29**, e202300898 (2023). 10.1002/chem.202300898 37024428

[22] Wang, L. et al. Structure-based design of an aromatic helical foldamer–protein interface. *Chem. Sci.* **16**, 12385–12396 (2025). 10.1039/d5sc01826a PMC12154549 40510324

[23] Azzarito, V. et al. Stereocontrolled protein surface recognition using chiral oligoamide proteomimetic foldamers. *Chem. Sci.* **6**, 2434–2443 (2015). 10.1039/c4sc03559c PMC5646261 29308155

[24] Stillman, N. H. et al. Protein mimetic 2D FAST rescues alpha synuclein aggregation mediated early and post disease Parkinson’s phenotypes. *Nat. Commun.* **15**, 3658 (2024). 10.1038/s41467-024-47980-4 PMC11061149 38688913

[25] Deepak, D. et al. DNA mimic foldamer recognition of a chromosomal protein. *Angew. Chem. Int. Ed.* **64**, e202422958 (2025). 10.1002/anie.202422958 39714421

[26] Reddy, P. S. et al. Structure elucidation of helical aromatic foldamer–protein complexes with large contact surface areas. *Chem. Eur. J.* **25**, 11042–11047 (2019). 10.1002/chem.201902942 31257622

[27] Kang, S. et al. COMBINES-CID: an efficient method for de novo engineering of highly specific chemically induced protein dimerization systems. *J. Am. Chem. Soc.* **141**, 10948–10952 (2019). 10.1021/jacs.9b03522 PMC6661117 31260282

[28] Gebauer, M. & Skerra, A. Engineered protein scaffolds as next-generation antibody therapeutics. *Curr. Opin. Chem. Biol.* **13**, 245–255 (2009). 10.1016/j.cbpa.2009.04.627 19501012

[29] Helma, J., Cardoso, M. C., Muyldermans, S. & Leonhardt, H. Nanobodies and recombinant binders in cell biology. *J. Cell Biol.* **209**, 633–644 (2015). 10.1083/jcb.201409074 PMC4460151 26056137

[30] Arrata, I., Barnard, A., Tomlinson, D. C. & Wilson, A. J. Interfacing native and non-native peptides: using Affimers to recognise α-helix mimicking foldamers. *Chem. Commun.* **53**, 2834–2837 (2017). 10.1039/c6cc09395g 28217789

[31] Wang, L. et al. Display selection of peptide ligands for helical aromatic foldamers. *Org. Biomol. Chem.* **23**, 4641–4647 (2025). 10.1039/d5ob00228a 40241662

[32] Huet, S., Gorre, H., Perrocheau, A., Picot, J. & Cinier, M. Use of the nanofitin alternative scaffold as a GFP-ready fusion tag. *PLoS ONE* **10**, e0142304 (2015). 10.1371/journal.pone.0142304 PMC4634965 26539718

[33] Mouratou, B. et al. Remodeling a DNA-binding protein as a specific in vivo inhibitor of bacterial secretin PulD. *Proc. Natl Acad. Sci. USA* **104**, 17983–17988 (2007). 10.1073/pnas.0702963104 PMC2084283 17984049

[34] Robinson, H. et al. The hyperthermophile chromosomal protein Sac7d sharply kinks DNA. *Nature* **392**, 202–205 (1998). 10.1038/32455 9515968

[35] Garlich, J. et al. Discovery of APL-1030, a novel, high-affinity nanofitin inhibitor of C3-mediated complement activation. *Biomolecules* **12**, 432 (2022). 10.3390/biom12030432 PMC8946527 35327625

[36] Correa, A. et al. Potent and specific inhibition of glycosidases by small artificial binding proteins (affitins). *PLoS ONE* **9**, e97438 (2014). 10.1371/journal.pone.0097438 PMC4019568 24823716

[37] Behar, G. et al. Tolerance of the archaeal Sac7d scaffold protein to alternative library designs: characterization of anti-immunoglobulin G Affitins. *Protein Eng. Des. Sel.* **26**, 267–275 (2013). 10.1093/protein/gzs106 23315487

[38] Kwon, S. et al. Interrogating the potential of helical aromatic foldamers for protein recognition. *Org. Biomol. Chem.* **22**, 9342–9347 (2024). 10.1039/d4ob01436g 39501876

[39] Kendhale, A. M. et al. Absolute control of helical handedness in quinoline oligoamides. *J. Org. Chem.* **76**, 195–200 (2011). 10.1021/jo1019442 21142109

[40] Gingras, A. R. et al. The structure of the C-terminal actin-binding domain of talin. *EMBO J.* **27**, 458–469 (2008). 10.1038/sj.emboj.7601965 PMC2168396 18157087

[41] Chen, M. C. et al. Structural basis of G-quadruplex unfolding by the DEAH/RHA helicase DHX36. *Nature* **558**, 465–469 (2018). 10.1038/s41586-018-0209-9 PMC6261253 29899445

[42] Vallade, M. et al. Assessing interactions between helical aromatic oligoamide foldamers and protein surfaces: a tethering approach. *Bioconjugate Chem.* **30**, 54–62 (2019). 10.1021/acs.bioconjchem.8b00710 30395443

[43] Bindl, D., Heinemann, E., Mandal, P. K. & Huc, I. Quantitative helix handedness bias through a single H vs. CH3 stereochemical differentiation. *Chem. Commun.* **57**, 5662–5665 (2021). 10.1039/d1cc01452h 33972976

[44] Koehler, V. et al. High-affinity hybridization of complementary aromatic oligoamide strands in water. *Angew. Chem. Int. Ed.* **62**, e202311639 (2023). 10.1002/anie.202311639 37804233

[45] Gradišar, H. et al. Design of a single-chain polypeptide tetrahedron assembled from coiled-coil segments. *Nat. Chem. Biol.* **9**, 362–366 (2013). 10.1038/nchembio.1248 PMC3661711 23624438

[46] Laniado, J. & Yeates, T. O. A complete rule set for designing symmetry combination materials from protein molecules. *Proc. Natl Acad. Sci. USA* **117**, 31817–31823 (2020). 10.1073/pnas.2015183117 PMC7749312 33239442

[47] Pletzer-Zelgert, J. et al. LifeSoaks: a tool for analyzing solvent channels in protein crystals and obstacles for soaking experiments. *Acta Crystallogr. D.* **79**, 837–856 (2023). 10.1107/S205979832300582X PMC10478636 37561404

[48] Shao, C., Liu, Z., Yang, H., Wang, S. & Burley, S. K. Outlier analyses of the Protein Data Bank archive using a probability-density-ranking approach. *Sci. Data.* **5**, 180293 (2018). 10.1038/sdata.2018.293 PMC6289109 30532050

[49] Buratto, J. et al. Structure of a complex formed by a protein and a helical aromatic oligoamide foldamer at 2.1 A resolution. *Angew. Chem. Int. Ed. Engl.* **53**, 883–887 (2014). 10.1002/anie.201309160 24288253

[50] Schake, P., Bolz, S. N., Linnemann, K. & Schroeder, M. PLIP 2025: introducing protein–protein interactions to the protein–ligand interaction profiler. *Nucleic Acids Res.* **53**, W463–W465 (2025). 10.1093/nar/gkaf361 PMC12230730 40347107

