# Supplementary material for: A protein–foldamer supramolecular synthon for self-assembled hybrid architectures
Source: Nat Chem. 2026 Aug 17;18(10):1819–27. doi: 10.1038/s41557-026-02222-6 (PMC13623658; doi:10.1038/s41557-026-02222-6)
Supplement: Supplementary file 1 [file 41557_2026_2222_MOESM1_ESM.pdf]

nature chemistry

Supplementary information https://doi.org/10.1038/s41557-026-02222-6

A protein–foldamer supramolecular synthon for self-assembled hybrid architectures

In the format provided by the authors and unedited

Table of contents

1. Materials and Methods 4

1.1 Chemical Synthesis 4

1.1.1 Materials 4

1.1.2 General methods for HPLC analysis and purification, LCMS and NMR analyses 4

1.1.3 Solid phase foldamer synthesis (SPFS) 5

1.1.4 Synthesis procedures 8

1.1.5 Characterization of new synthetic compounds 17

1.2 Biochemistry 33

1.2.1 Generation and identification of Nanofitin C10 33

1.2.2 Materials and general methods 34

1.2.3 Expression and purification of proteins 35

1.3 Biophysical measurements and binding analyses 38

1.3.1 Circular dichroism (CD) spectroscopy 38

1.3.2 Analytical size exclusion chromatography 38

1.3.3 Mass spectrometry 38

1.3.4 Bio-layer interferometry (BLI) 38

1.4 NMR Structure Elucidation & MD Simulations 39

1.4.1 Experimental procedure 39

1.4.2 Chemical shift assignment 39

1.4.3 Initial structure calculation of the **1c**•C10 complex 40

1.4.4 Restrained molecular dynamics on **1c**•C10 41

1.4.5 Data deposition 43

1.5 Molecular modelling and other computational tools 43

1.6 Crystallography 44

1.6.1 Crystallization of C10 coiled-coil dimer, *P*-**2b**•C10CE, (*P*-**3**•C10)$_2$ and **4**•C10CE 44

1.6.2 X-ray data collection, structure determination and refinement 44

2. Supplementary Figures 45

Supplementary Figure 1 | Ribosome display selection against **1a**. 45

Supplementary Figure 2 part 2 | Crystal structure of the selected Nanofitin C10 fused to a coiled coil domain at 1.4 Å. 47

Supplementary Figure 3 | NMR spectroscopy of unbound Nanofitin C10. 48

Supplementary Figure 4 | NMR spectroscopy of Nanofitin C10 in complex with dodecaamide foldamer **1c**. 49

Supplementary Figure 5 | 2D filtered NOESY spectra on [^{13}C,^{15}N]-C10 bound to natural abundance **1c**. 50

Supplementary Figure 6 | Nomenclature used for **1c** NMR assignment and structure determination. 51

Supplementary Figure 7 | NMR distance restraints mapped onto representative model of **1c**•C10. 52

Supplementary Figure 8 | Atomic fluctuations during the microsecond production MD of the **1c**•C10 complex. 53

Supplementary Figure 9 |Analysis of intermolecular hydrogen bonding over the course of the MD simulation of the **1c**•C10 complex. 54

Supplementary Figure 10 | Solution phase binding assessment of AOFs towards C10. 55

Supplementary Figure 11 | Synthetic pathway towards the C_2 symmetrical 31mer **4** bearing two C10 binding epitopes including selected analytics for progress monitoring. 57

Supplementary Figure 12 | Comparison of apo and foldamer-bound C10 structures obtained by NMR and Xray crystallography. 58

Supplementary Figure 13 part 2 | Conservation of the C10-foldamer binding interface across five observed instances in three different crystal structures. 60

Supplementary Figure 14 part 2 | Extended view of the (*P*-**3**•C10)$_2$ complex crystal structure .. 62

Supplementary Figure 15 | Native nESI-MS and MSMS of the C10CE protein in complex with **2b**. 63

Supplementary Figure 16 | Native nESI-MS of the C10CE protein alone (a) and in complex with the 31 unit-long AOF **4** (b). 64

Supplementary Figure 17 | Extended views of the crystal structure of the coiled-coil extended C10 (C10CE) in complex with pentaamide *P*-**2b**. 65

Supplementary Figure 18 | Additional views on the crystal structure of **4**•C10-CE. 66

3. Supplementary Tables 67

Supplementary Table 1 | X-ray crystallography data collection and refinement statistics. 67

Supplementary Table 2 | Atom equivalence and 1H chemical shift assignment of C10-bound foldamer **1c**. 69

Supplementary Table 3 | NMR and refinement statistics for foldamer-bound Nanofitin C10. 70

Supplementary Table 4 | Intermolecular NMR distance restraints for **1c**•C10 from ARIA/CNS. 71

Supplementary Table 5 | Comparison of the cognate C10-foldamer binding interface across all observed crystal structures. 72

4. Supplementary Videos 73

Supplementary Video 1 (separate file). MD simulation of the 1c•C10 complex. 73

Supplementary Video 2. (separate file). Animated view of the (*P*-**3**•C10)$_2$ complex. 73

Supplementary Video 3 (separate file) Animated view of the 8+8 assembly of four (*P*-**3**•C10)$_2$ complexes. 73

Supplementary Video 4 (separate file). Animated view of the cyclic X-shape assembly of two foldamers *P*-**2**b and two C10 coiled-coil dimers. 73

5. Methods-only References 74

1. Materials and Methods

1.1 Chemical Synthesis

1.1.1 Materials

All chemicals were purchased from commercial suppliers (*Sigma-Aldrich*, *Fisher Scientific*, *IRIS Biotech*, *ABCR*) and used without further purification unless stated otherwise. Regular and low loading (LL)-Wang resins were purchased from *Merck-Novabiochem* and SASRIN™ resin was purchased from *Bachem*. Cl-MPA Protide resin was purchased from *CEM* (this resin has since been discontinued). Solvents were purchased from *Fisher Scientific* (cyclohexane, ethyl acetate, dichloromethane (DCM), methanol, tetrahydrofurane (THF) and acetone, analytical grade), *IRIS Biotech* (*N*-methyl-2-pyrrolidinone (NMP)) or *Carlo Erba* (*N*,*N*-dimethylformamide (DMF), peptide grade) and used without further purification. Anhydrous DCM and THF were obtained from a SPS-800 Solvent Purification System (*MBraun*). *N*,*N*-diisopropylethylamine (DIPEA), NEt_3 and $CHCl_3$ were freshly distilled over CaH_2 prior to use. HPLC grade acetonitrile (MeCN, *Fisher Scientific*) and ultra-pure water (dispensed from OmniaPure xs^{basic}, *Stakpure*) were used for RP-HPLC analyses and purification. LCMS grade MeCN (*Fisher Scientific*) was used for LCMS analyses.

1.1.2 General methods for HPLC analysis and purification, LCMS and NMR analyses

RP-HPLC analyses were performed on an Ultimate 3000 HPLC system (*Thermo Fischer Scientific*) equipped with an UV diode array detector, monitoring absorbance at 254 nm and 300 nm if not stated otherwise, using a Nucleodur C18 Htec (4.6 × 100 mm, 5 µm, *Macherey-Nagel*) or C8 Gravity column (4 × 100 mm, 5 µm, *Macherey-Nagel*). For acidic RP-HPLC analyses, 0.1% trifluoroacetic acid (TFA) in water (solvent A) and 0.1% TFA in acetonitrile (solvent B) were used as the mobile phase at a flow rate of 1 mL/min. All the RP-HPLC analyses were run at 50°C.

Semi-preparative RP-HPLC purification was performed on a Waters system with a 2707 autosampler, a 2489 UV/Visible detector, a 2545 quaternary gradient module and a fraction collector III (*Waters*), using a vario-prep Nucleodur C18 Htec column (21× 125 mm, 5 µm, *Macherey-Nagel*) at a flow rate of 25 mL/min. Semi-semi-preparative RP-HPLC was performed on an Ultimate 3000 HPLC system, using a Nucleodur C18 Gravity column (10 × 250 mm, 5 µm, *Macherey-Nagel*) at a flow rate of 5 ml/min. The same solvent composition to the analytical conditions was used.

LC-MS analyses were recorded on an Ultimate 3000 HPLC system, coupled to a micrOTOF II mass spectrometer (*Bruker Daltonics*) with electron spray ionization (ESI). The LC column used was a Nucleodur Gravity Ec column (2 × 50 mm, 1.8 µM, *Macherey-Nagel*). All LC analyses were run at 50°C. The MS spectrometer was calibrated, prior to analysis, in positive and negative mode by direct infusion of an ESI-Low Concentration Tuning Mix (*Agilent Technologies*).

^{1}H NMR spectra were recorded on Avance III HD 500 MHz BioSpin and Avance NEO BioSpin 700 MHz spectrometers (*Bruker*). All chemical shifts are reported in ppm and calibrated against residual solvent signals of CD_3CN (δ 1.94 ppm), DMSO-d_6 (δ 2.50 ppm), $CDCl_3$ (δ 7.26 ppm) and DMF-d_7 (δ 8.03 ppm). In the case of oligomers **2a**, **5**, **6** and **7**, analyses were performed in CD_3CN/H_2O (1:1; vol/vol) and recorded with water suppression with excitation sculpting using the zgesgp pulse sequence from the Bruker pulse sequence library. Coupling constants (*J*) are reported in Hz. Signal multiplicities are reported as *s*, singlet; *d*, doublet; *t*, triplet; *q*, quartet; *p* pentet; and *m*, multiplet.

1.1.3 Solid phase foldamer synthesis (SPFS)

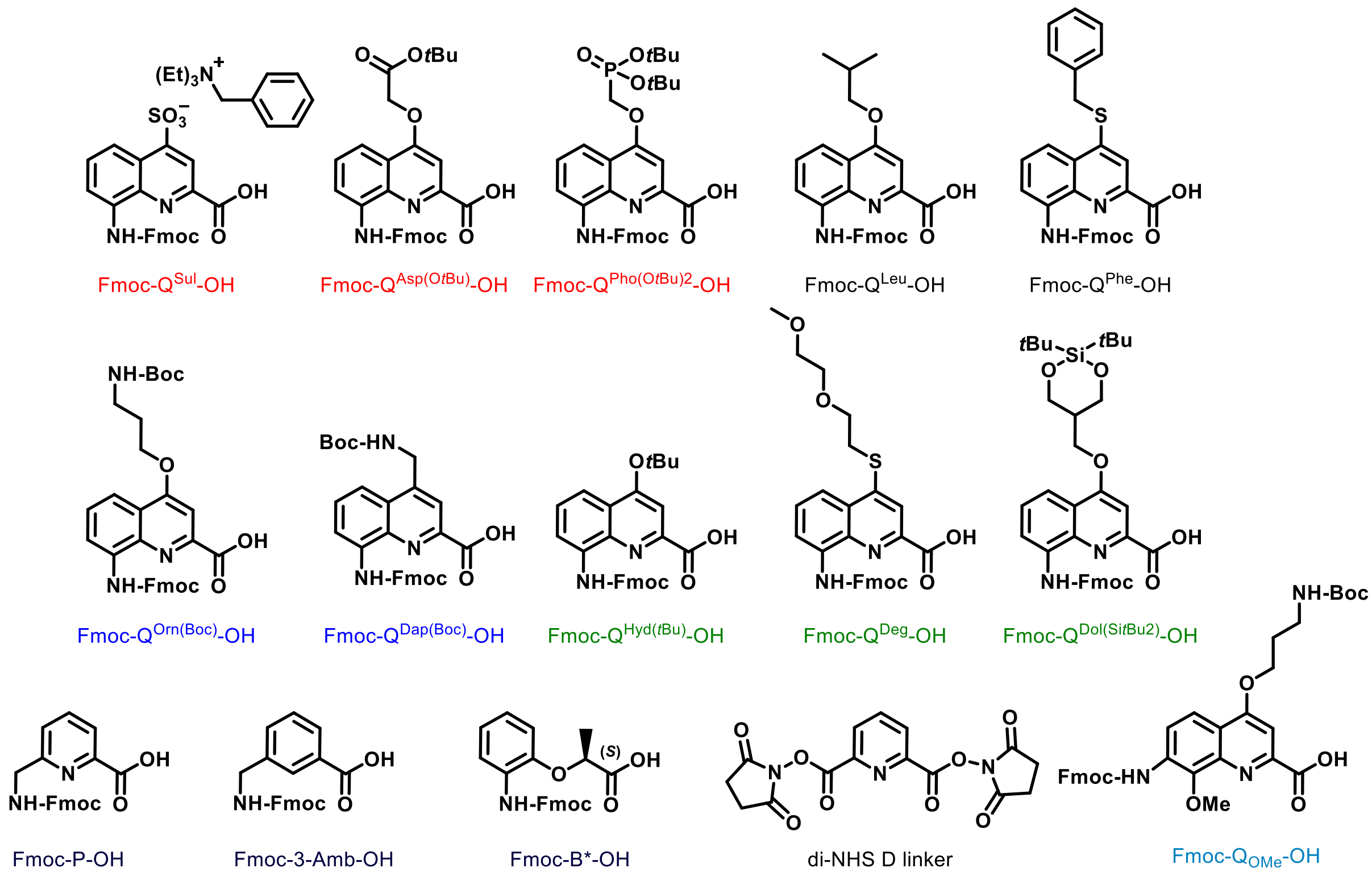

All protected Fmoc-Q$^{Xxx(PG)}$-OH and Fmoc-Q$_{OMe}$-OH, as well as Fmoc-P-OH and Fmoc-B*-OH monomers (shown above) used for the solid phase synthesis of the oligomers were prepared following reported synthetic protocols[44, 45, 51-57].

SPFS was performed following recently reported conditions either manually, for sequences **3, 5-7**, on a Discover Bio® microwave synthesizer (*CEM*) using open reaction vessels and an internal fiber optic probe for temperature control, or in automated mode, using a PurePep® Chorus peptide synthesizer (*Gyros-Protein Technologies*) for sequences **1c**, **2a**, **2b** and **fragment A**, the precursor of **4**[21]. Generally, all monomers were activated as their respective acid chlorides, by applying *in situ* Appel's conditions in the presence of PPh_3, trichloroacetonitrile (TCAN), and 2,4,6-collidine as base. For the synthesis of **2b** the activation procedure was modified to prevent deprotection of the acid labile *t*Bu phosphonate side chain during activation (see procedure below).

Foldamer **3** was synthesized according to recently reported SPFS using Ghosez's reagent for acid chloride activation on a Discover Bio® microwave synthesizer (*CEM*)[21,45].

The syntheses of compounds **1a** and **1b** have been recently described, alongside optimized Fmoc deprotection conditions. Oligomer **1c** was synthesized following the same procedures on Cl-MPA protide resin[38].

Additionally, we have recently developed a new loading procedure on Wang resins using DIC/OxymaPure conditions (see below).

Loading of the first quinoline unit on Wang resin (exemplified for the sequences **5**-**7**).

The first step consisted in loading the Fmoc-$Q^{Asp(OtBu)}$-OH to the Wang Resin (1 $mmol.g^{-1}$). 200 mg of resin (0.2 mmol) was dispensed in a 10 mL syringe equipped with a frit and swollen in 5 mL of dry DCM for at least 30 min. Fmoc-$Q^{Asp(OtBu)}$-OH (0.4 mmol, 2 equiv.) was dissolved in 1.5 mL of DCM. OxymaPure (2 equiv.) was dissolved in 1 mL of DMF and added to the monomer solution. Subsequently, the mixture was poured to the freshly filtered resin. In a separate vial, DMAP (0.1 equiv.) was dissolved in a minimum volume of DCM (0.5 mL). DIC (2 equiv.) was added to the suspended resin directly followed by the solution of DMAP. The resin was next gently shaken overnight at room temperature. After resin filtration and washings with DCM, a capping step was performed using acetic anhydride in DCM (1:1, vol/vol, 4 mL) for at least 30 min. The resin was lastly washed thoroughly with DCM. Loading determination by UV absorption spectroscopy gave a loading yield of 40% (0.40 $mmol.g^{-1}$, 0.08 mmol).

The same conditions were used for sequences **2a**, **2b** and **3** on LL-Wang resin (0.43 $mmol.g^{-1}$).

General procedure for Fmoc deprotection

The resin was washed three times with a solution of DCM:NMP (80:20; vol/vol), before adding a solution of 2% DBU in NMP. The Fmoc deprotection was performed for 2 × 3 min. After deprotection, the resin was washed two times with 20% (vol./vol.) NMP in DCM and then three times with dry THF.

In situ activation and aromatic monomer couplings

The aromatic monomers (Q, P, Amb and B*) were coupled on Wang resin-bound amines with *in situ* activation using an excess of three equivalents of monomer relative to the resin loading. Each coupling was performed twice at 50°C for 15 min[38].

Coupling of the Biotin-PEG(4)-COOH linker

The Biotin-PEG(4) linker (2 equiv. relative to resin loading) was coupled manually on the resin-bound H-Amb-foldamer by applying peptide coupling conditions with the use of PyBOP (2.3 equiv.) and DIPEA (4 equiv.) in dry DMF overnight at room temperature. Coupling efficiency was assessed by performing a colorimetric test with 2,4,6-trinitrobenzene sulfonic acid (TNBS).

TBAF deprotection of the di-*tert*-butyl silylether protection group of Q^{Dol}

Prior to TFA cleavage, the silyl protecting group on the diol side chain was removed using tetrabutylammonium fluoride (TBAF, 1 M in THF). The resin was suspended in dry THF and TBAF (8 equiv. per Q^{Dol}) was added. The deprotection took place under microwave irradiation (50 W, ramp to 50°C for 5 min, hold at 50°C for 15 min) and this step was repeated once. The resin was then thoroughly washed with DMF, and DCM prior to TFA cleavage.

TFA cleavage of the oligomers from the Wang resin

Cleavage of the oligomer from the Wang resin was performed using a mixture of TFA, tri-isopropyl silane (TIS) and H_2O (95: 2.5: 2.5; vol/vol/vol), for 2 h at room temperature. The crude oligomer was precipitated with diethylether (Et_2O), redissolved in H_2O/MeCN and lyophilized.

1.1.4 Synthesis procedures

1.1.4.1 Synthesis of **1c**

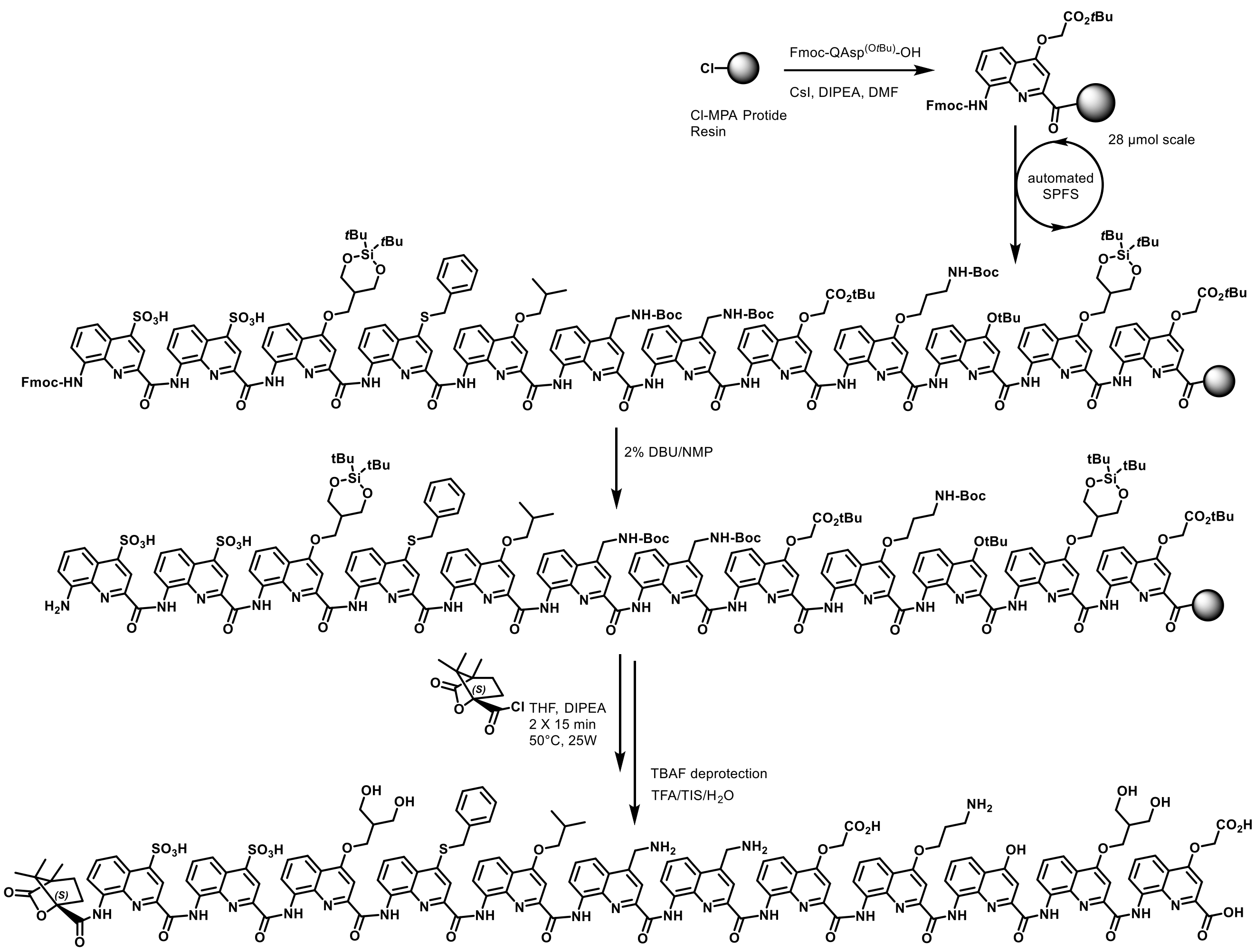

1c

Compound 1c: Oligomer **1c** was synthesized on Cl-MPA Protide resin (0.17 mmol.g^{-1}, 28 µmol scale after first monomer loading using CsI and DIPEA and following the same protocol as for **1a** and **1b**[58]. The N-terminal camphanyl moiety was introduced according to recently published procedures[38]. After TBAF deprotection of the silyl ether, TFA cleavage and purification by preparative RP-HPLC, compound **1c** was obtained as a yellow powder in 17% yield (5.2 mg, 5 µmol).

^{1}H NMR (500 MHz, DMSO-*d*$_6$): δ = 10.94 (m, 2H), 10.71 (s, 1H), 10.59– 10.43 (m, 3H), 10.32 (s, 1H), 10.04 (m, 2H), 9.73 (s, 1H), 9.05 (s, 1H), 8.32 (d, *J* = 8.1 Hz, 1H), 8.23 (d, *J* = 8.2 Hz, 1H), 8.14 (s, 1H), 8.01 (d, *J* = 7.2 Hz, 1H), 7.93 (d, J = 7.2 Hz, 1H), 7-82– 6.63 (m, 41H), 6.36 (s, 1H), 6.33 (s, 1H), 6.16 (s, 1H), 6.12 (s, 1H), 5.97 (s, 1H), 5.91 (s, 1H), 5.88 (s, 1H), 5.56 (s, 1H), 4.85 – 3.91 (m, 19 H), 3.87 – 3.55 (m, 14H), 3.50 (s, 2H), 2.35 (bp, 5H), 2.27 (m, 1H), 2.12 (m, 1H), 1.98 (m, 1H), 1.57 (m, 2H), 1.46 (m, 1H), 0.39 (d, *J* = 8.4 Hz, 6H), -0.12 (s, 3H).

HRMS (ESI⁺) *m/z* calculated for $C_{158}H_{133}N_{27}O_{37}S_3$ 1033.2842 $[M+3H]^{3+}$; found 1033.2800 $[M+3H]^{3+}$.

1.1.4.2 Synthesis of **2a**

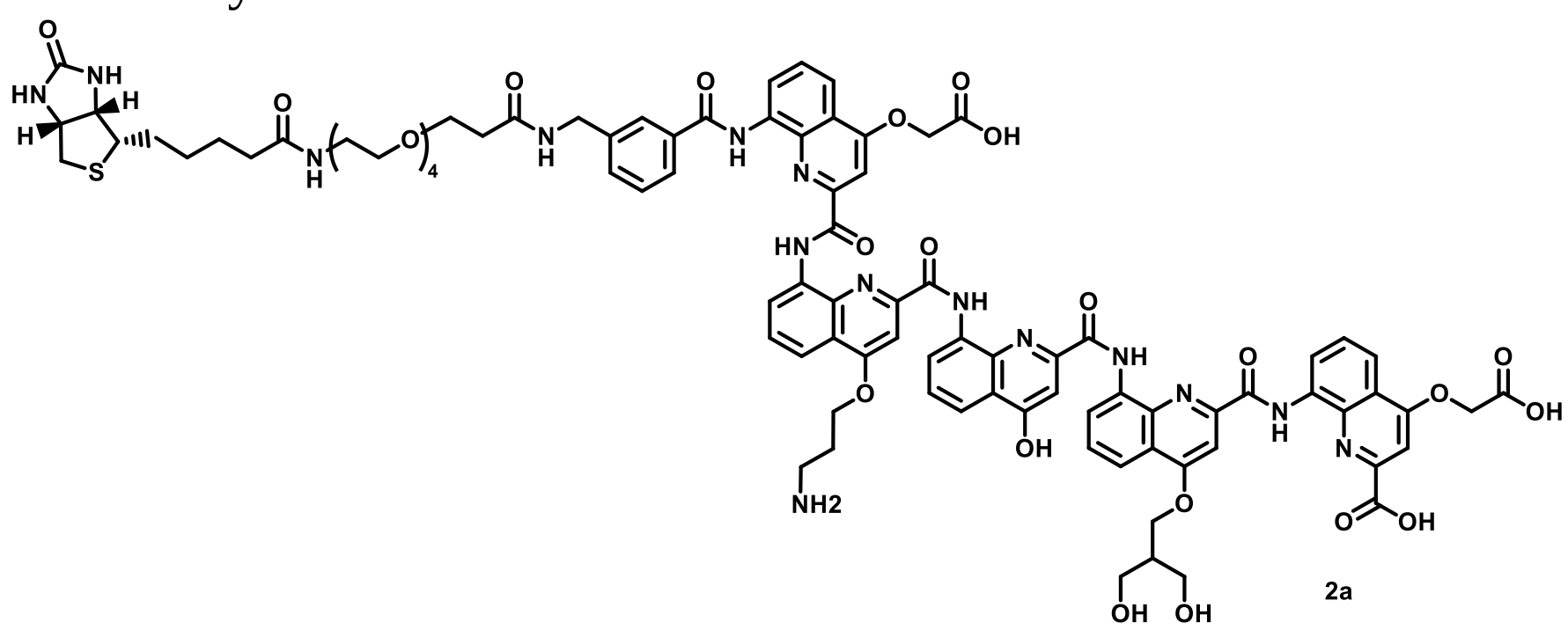

2a

Compound 2a: Oligomer **2a** was synthesized on LL-Wang resin (20 µmol scale). After TBAF deprotection of the silyl ether, TFA cleavage, lyophilization and purification by preparative RP-HPLC, compound **2a** was obtained as a yellow powder in 39% yield (14.2 mg, 7.82 µmol).

^{1}H NMR (500 MHz, CD_3CN/H_2O (1:1, vol/vol)): δ = 11.18 (s, 2H), 11.05 (s, 1H), 10.99 (s, 1H), 9.46 (s, 1H), 7.97 (s, 2H), 7.88 (d, *J* = 9.0 Hz, 1H), 7.75 (d, *J* = 9.1 Hz, 3H), 7.64 (t, *J* = 9.2 Hz, 1H), 7.51 (t, *J* = 7.2 Hz, 2H), 7.31 (t, *J* = 8.3 Hz, 1H), 7.22 (t, *J* = 8.5 Hz, 1H), 7.11 (t, *J* = 8.3 Hz, 1H), 7.03 (s, 1H), 6.94 – 6.87 (m, 4H), 6.85 (s, 1H), 6.48 (t, *J* = 8.1 Hz, 1H), 6.42 (s, 1H), 6.16 (s, 1H), 6.02 (s, 2H), 5.01 (d, *J* = 15.6 Hz, 1H), 4.94 (d, *J* = 15.3 Hz, 1H), 3.82 (d, *J* = 6.8 Hz, 1H), 3.56 – 3.48 (m, 1H), 3.29-3.41 (m, 18H), 3.13 (q, *J* = 5.6 Hz, 3H), 3.09 – 2.97 (m, 2H), 2.74 (dd, *J* = 5.0, 2.3 Hz, 1H), 2.55 (dd, *J* = 13.1, 3.5 Hz, 2H), 2.45 – 2.36 (m, 4H), 2.29 - 2.19 (m 4H), 1.99 (td, *J* = 7.9, 3.2 Hz, 2H), 1.49 (dt, *J* = 16.2, 6.6 Hz, 1H), 1.42 – 1.34 (m, 5H), 1.22 - 1.10 (m, 5H).

HRMS (ESI⁺) *m/z* calculated for $C_{90}H_{94}N_{15}O_{25}S_1$ 1816.6261 $[M+H]^+$; found 1816.6172 $[M+H]^+$.

1.1.4.3 Synthesis of **2b**

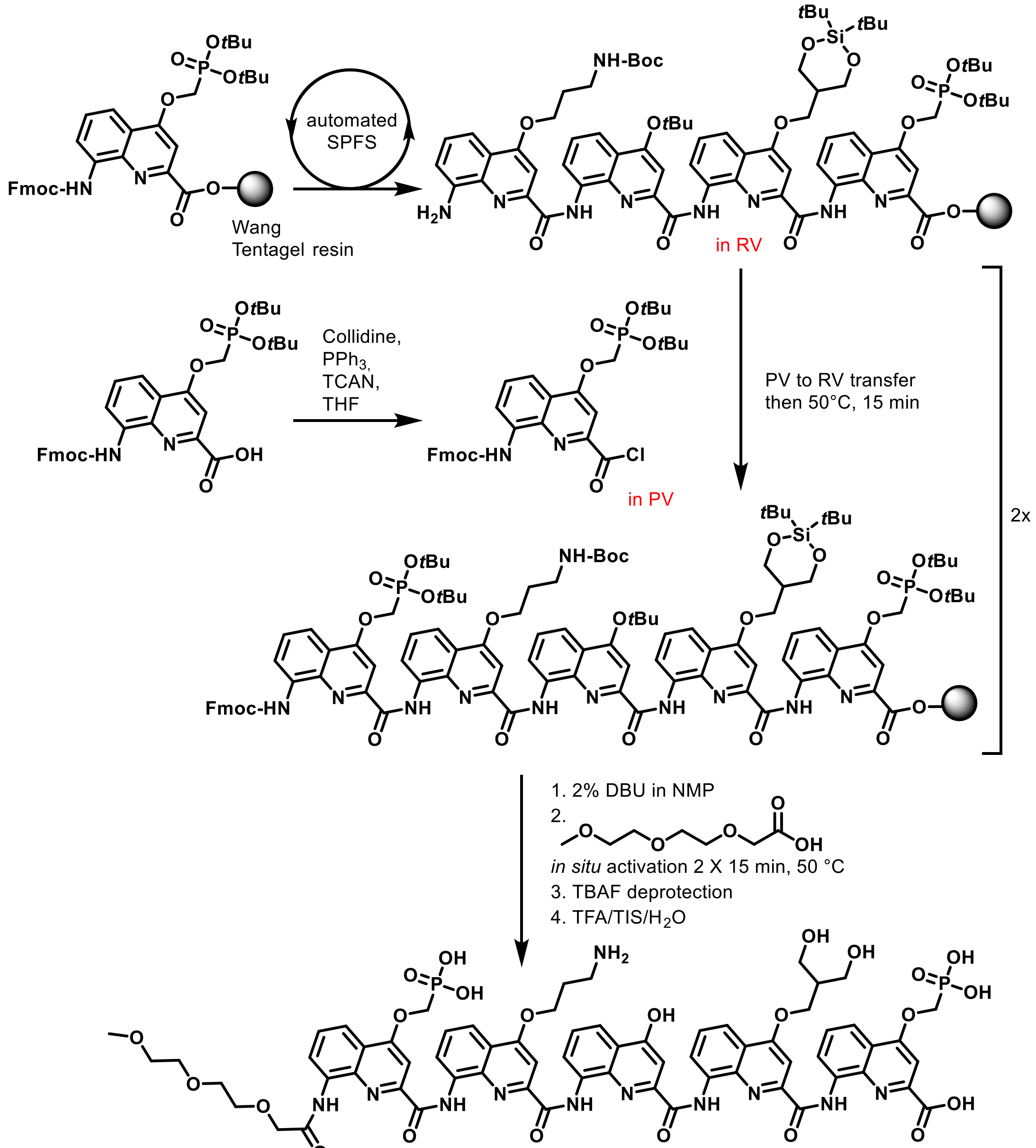

Compound 2b: Oligomer **2b** was synthesized on LL-Wang resin (30 µmol scale) using the automated SPFS procedure and loading with DIC/OxymaPure conditions. However, the coupling of the final aromatic monomer, Fmoc-$Q^{Pho(OtBu)2}$-OH, was carried out using a distinct protocol due to its acid sensitivity. The collidine solution in dry THF (9 equiv. relative to the resin loading) was first dispensed in the pre-activation vessel (PV), followed by the addition of Fmoc-$Q^{Pho(OtBu)2}$-OH (3 equiv.), PPh_3 (8 equiv.) and lastly the TCAN (9 equiv.) solution. Acid chloride activation was performed for 1 min in the PV. The solution was then dispensed to the RV containing the resin-bound amine tetramer (PV to RV step). Coupling was performed under heat induction at 50°C for 15 min. After resin filtration and washing twice with dry THF, the same procedure was repeated once.

After TBAF deprotection of the silyl ether, TFA cleavage, lyophilization and purification by preparative RP-HPLC in acidic conditions, compound **2b** was obtained as a yellow powder in 39% yield (16.7 mg, 11.6 μmol).

^{1}H NMR (500 MHz, DMSO-*d*$_6$): δ = 11.57 (s, 3H), 11.41 (s, 1H), 9.58 (s, 1H), 8.57 – 8.20 (m, 2H), 8.17 – 7.50 (m, 16H), 7.42 (t, *J* = 7.9 Hz, 1H), 7.37 – 7.14 (m, 4H), 6.85 – 6.47 (m, 3H), 4.75 – 4.24 (m, 5H), 4.14 (s, 3H), 4.05 (s, 1H), 2.70 (s, 10H).

HRMS (ESI$^+$): *m/z* calculated for $C_{66}H_{65}N_{11}O_{23}P_2$ 1442.3803 $[M+H]^+$; found 1442.3383 $[M+H]^+$.

1.1.4.4 Synthesis of **3**

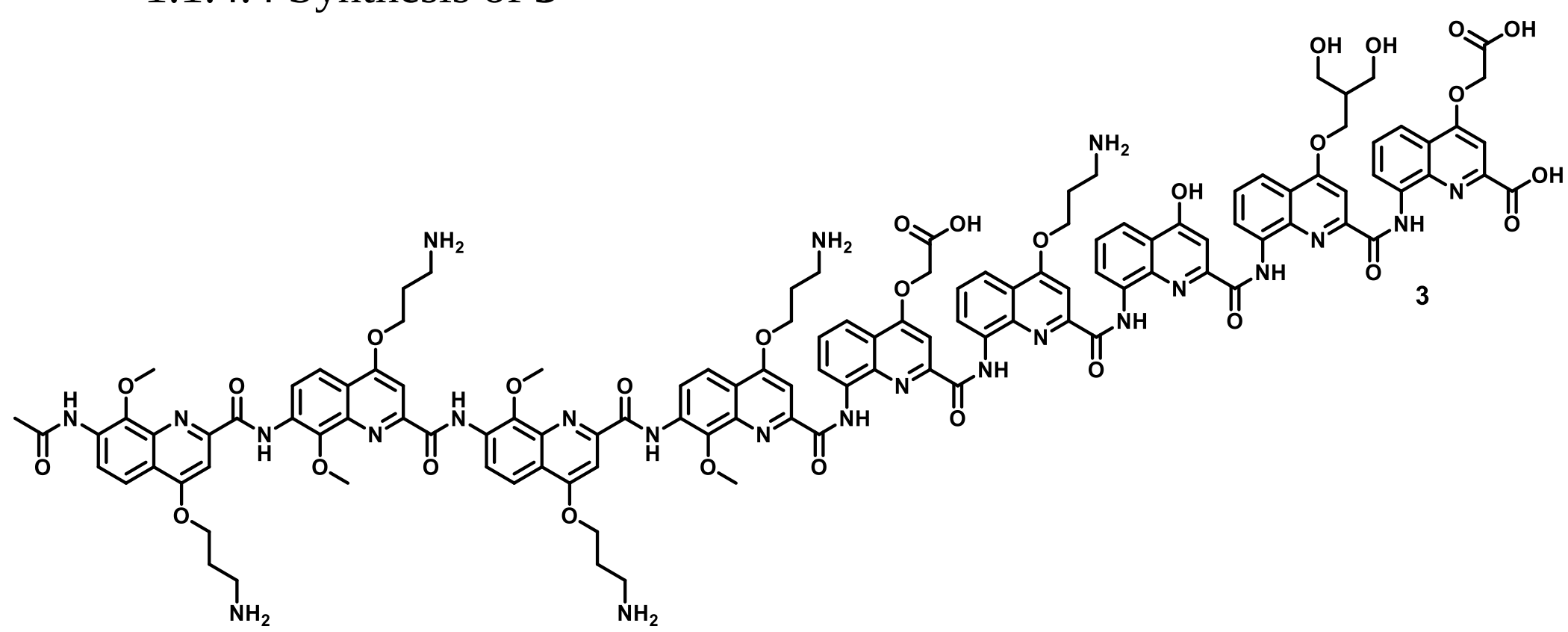

3

Oligomer **3** was synthesized on LL-Wang resin (21 μmol scale). The conditions of coupling and deprotection were the same to those recently published[21, 45]. Fmoc-Q^{Xxx}-Cl and Fmoc-Q_{OMe}-Cl (3 equiv. relative to the resin loading) were freshly prepared in the presence of chloroenamine and DIPEA (6 equiv.) was employed as base. Couplings were performed in dry THF (3 mL) under microwave irradiation (2×15 min, 50°C, 25 W). N-terminal acetylation was performed following the same conditions to those reported[45].

^{1}H NMR (700 MHz, DMSO-*d*$_6$): δ = 13.25 (s, 1H), 11.62 (s, 1H), 11.53 (s, 1H), 11.51 (s, 1H), 11.37 (s, 1H), 11.28 (s, 2H), 11.20 (s, 1H), 11.09 (s, 1H), 9.83 (s, 1H), 8.98 (s, 1H), 8.61 (s, 1H), 8.41 (s, 3H), 8.27 (dd, *J* = 17.2, 8.0 Hz, 4H), 8.22 (s, 4H), 8.11 (d, *J* = 7.3 Hz, 3H), 8.09 (s, 3H), 8.05 (s, 5H), 7.86 (d, *J* = 10.5 Hz, 3H), 7.79 (d, *J* = 7.5 Hz, 3H),7.74 (t, *J* = 6.8 Hz, 3H), 7.67 (t, *J* = 7.4 Hz, 4H), 7.61 (t, *J* = 7.6 Hz, 2H), 7.55 (t, *J* = 7.7 Hz, 1H), 7.33 – 7.21 (m, 4H), 6.90 (d, *J* = 9.6 Hz, 2H), 6.55 (t, *J* = 7.7 Hz, 1H), 6.51 (s, 1H), 6.40 (s, 1H), 4.84 (d, *J* = 16.0 Hz, 2H), 4.79 (d, *J* = 7.8 Hz, 2H), 4.69 (t, *J* = 14.5 Hz, 3H), 4.65 – 4.58 (m, 7H), 4.54 (d, *J* = 7.9 Hz, 2H), 4.42 (s, 4H), 4.28 (s, 3H), 4.21 (d, *J* = 9.5 Hz, 2H), 4.02 (t, *J* = 7.3 Hz, 2H), 3.94 (t, *J* = 6.9 Hz, 2H), 3.87 (s, 3H), 3.79 (d, *J* = 4.1 Hz, 4H), 3.58 –3.52 (m, 7H), 3.33 (s, 19H), 3.21 – 3.15 (m, 40H), 2.35 – 2.31 (m, 6H), 2.10 – 2.05 (m, 2H), 2.01 – 1.96 (m, 1H), 1.62 – 1.54 (m, 29H), 1.48 (q, *J* = 7.1 Hz, 2H), 1.25 (d, *J* = 8.5 Hz, 9H), 0.86 (tt, *J* = 7.5, 4.6 Hz, 2H).

HRMS (ESI$^+$): *m/z* calculated for $C_{119}H_{113}N_{23}O_{30}$ 1173.4099 $[M+2H]^{2+}$; found 1173.3991 $[M+2H]^{2+}$.

1.1.4.5 Synthesis of **fragment A**, precursor of **4**

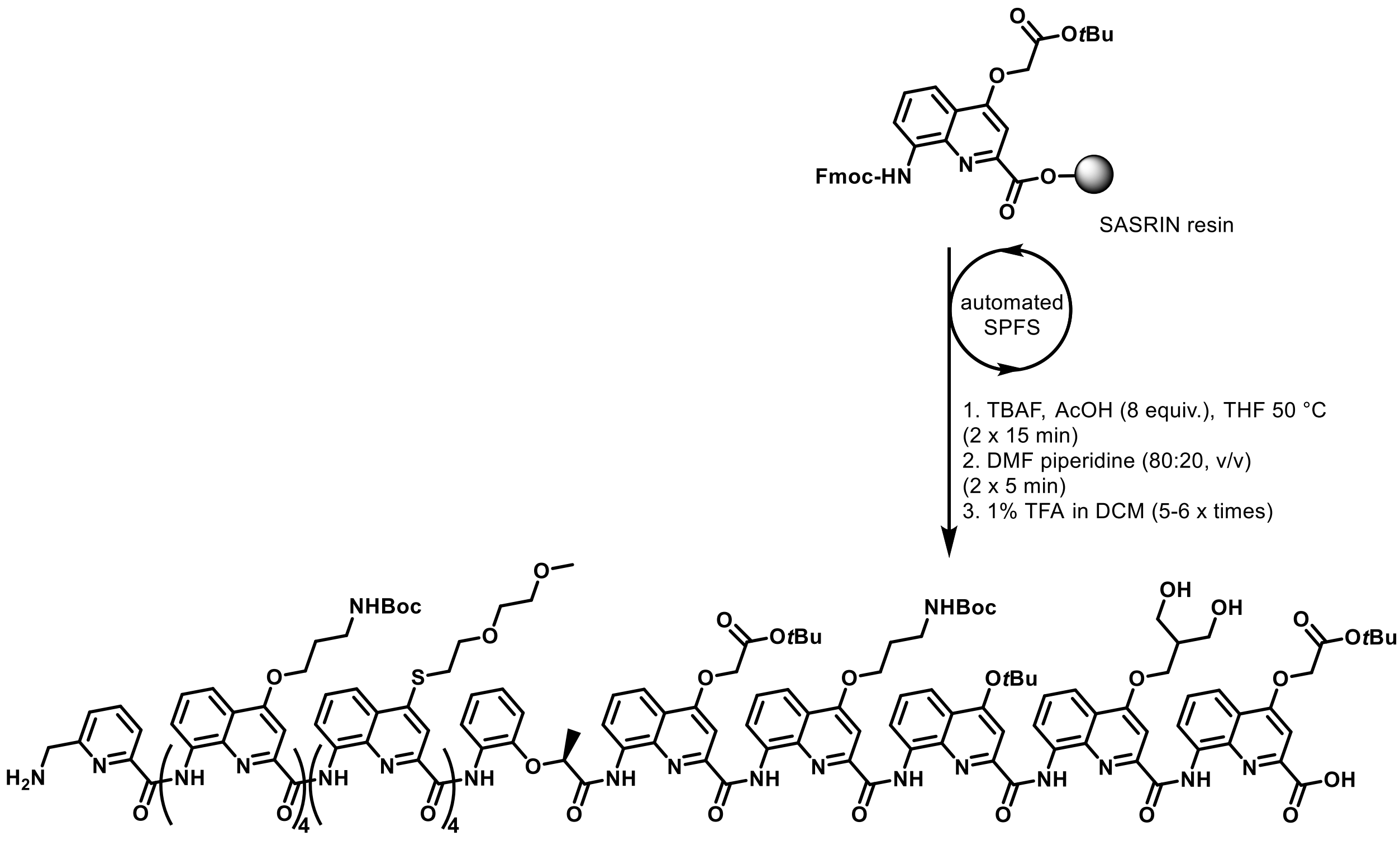

fragment A

The illustrated overview of the SPFS strategy towards the 31-unit C_2 symmetrical sequence **4** is shown in Supplementary Fig. 11. The sidechains protected 15mer precursor **fragment A** was synthesized by automated SPFS procedure on SASRIN at a 20 µmol scale (according to the UV loading determination of the resin-bound Fmoc-$Q^{Asp(OtBu)}$-OH see below).

<u>Fmoc-$Q^{Asp(OtBu)}$-OH loading on SASRIN resin (0.8-1.2 $mmol.g^{-1}$)</u>

Fmoc-$Q^{Asp(OtBu)}$-OH (0.150 mmol, 0.5 equiv.) and HBTU (0.225 mmol, 1.5 equiv.) were dissolved in dry DMF (5 mL), followed by the addition of freshly distilled DIPEA (104 µL, 0.6 mmol, 2 equiv.). The activated monomer was added to resin (300 mg, 0.3 mmol, 1.0 equiv.), and loading was carried out under microwave irradiation (50 ºC, 25 W, 20 min). The resin was next washed with DMF. The UV loading determination was next performed. In case of insufficient loading a second loading cycle was performed using 0.7 equiv. of Fmoc-$Q^{Asp(OtBu)}$-OH, 2.1 equiv. of HBTU and 2.8 equiv. of DIPEA.

<u>SASRIN resin capping</u>

After loading the first quinoline monomer, the resin was capped by adding a solution of benzoyl chloride in a DCM:pyridine solvent mixture (1:3:1; vol/vol/vol) for 30 min. The resin was then thoroughly washed with DCM[16].

SPFS procedure

Fragment A was synthesized on the PurePep Chorus synthesizer using *in situ* activation conditions in a 20 μmol scale.

TBAF deprotection

The silyl protecting group of Q^{Dol} was removed with a TBAF:AcOH solution (15% AcOH in 1 M TBAF in THF, 0.4 mL) in dry THF (V_{tot} = 3 mL), using microwave irradiation (2 × 15 min, 50°C, 25 W). The resin was washed with THF and DMF (3 × 4 mL each).

Fmoc deprotection

The N-terminal Fmoc group was removed using 3 mL DMF:piperidine (4:1; vol/vol) for 2 × 5 min. The resin was subsequently washed with DMF and lastly with DCM (3 × 4 mL each).

Cleavage from SASRIN resin

The cleavage of the (partly) protected foldamer **fragment A** from the SASRIN resin was performed using 1% TFA in DCM. The resin was placed into a 10 mL syringe equipped with a frit, washed with DCM (5 × 4 mL), and treated with 1% TFA in DCM for 2 min (2 mL). After each cycle of cleavage, the solution was quenched with saturated $NaHCO_3$ (aq.). The cleavage cycles were repeated until no more pink coloration of the resin was observed. The cleavage solutions were combined, extracted with DCM, and the solvent was removed by rotary evaporation.

Fragment A purification

The protected **fragment A** was purified by semi-preparative HPLC in neutral conditions (ultra pure water as solvent A and CH_3CN as solvent B) to afford pure **fragment A** in 8% yield (7.2 mg, 1.65 μmol).

1.1.4.6 Synthesis of **4**

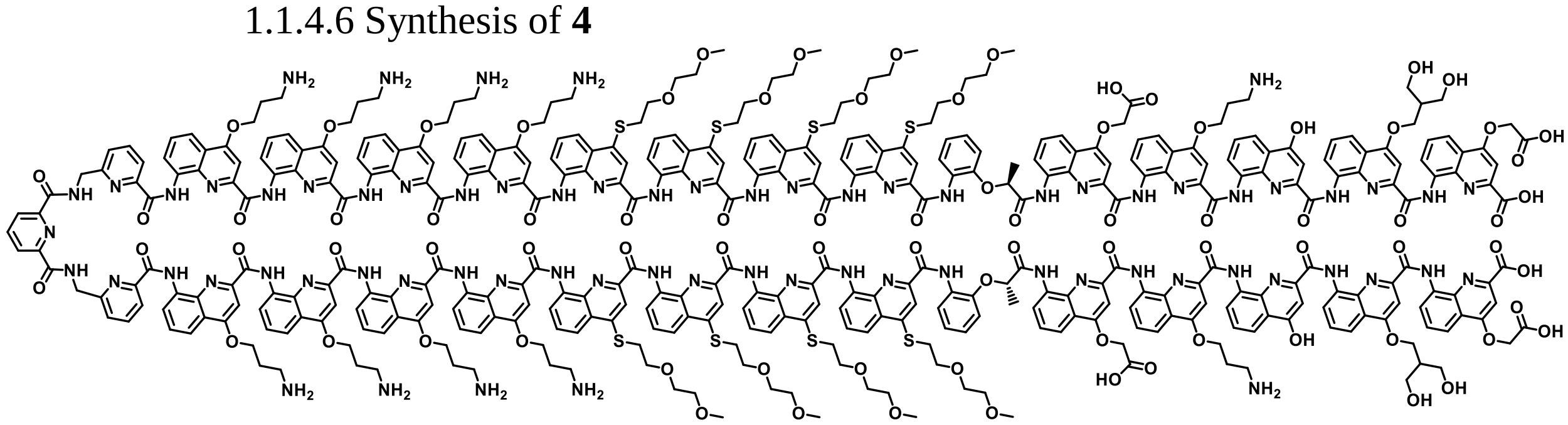

4

The di-NHS ester of pyridin-2,6-dicarbocylic acid D ($D(NHS)_2$) was first prepared according to literature procedures[59].

A DIPEA stock solution was next prepared by dissolving 17.5 µL of freshly distilled DIPEA into dry DMF (V_{tot} = 0.5 mL). A second stock solution was prepared by dissolving 3 mg of $D(NHS)_2$ in dry DMF (0.5 mL).

Fragment A (7.2 mg, 1.65 µmol, 2 equiv.) was dissolved in dry DMF (0.1 mL), degassed twice by freeze-pump-thaw cycles, and placed under N_2 environment. The concentration of the solution was determined by measuring the absorbance of a diluted sample at 375 nm, using an average extinction coefficient per Q unit ($\varepsilon = 13 \times 2675$ $M^{-1}.cm^{-1}$) for the calculation according to the Lambert-Beer law.

50 µL (12 equiv.) of DIPEA stock solution was added to the **fragment A** solution and stirred for 15 min. 50 µL of the $D(NHS)_2$ stock solution (0.825 µmol, 1 equiv.) was added dropwise to the previous solution over 20 min.

The progress of the reaction was monitored by RP-HPLC analysis and was completed in 4 h. Solvent was removed by freeze-drying the crude reaction solution. Subsequently, the (partly) protected dimer intermediate was deprotected using TFA:TIS:H_2O (95:2.5:2.5; vol/vol/vol) for 2 hours, lyophilized and purified by semi-preparative RP-HPLC with acidic conditions.

After lyophilization, compound **4** was incubated in DMF-d_7 for two days and its diastereomeric purity was monitored by ^{1}H NMR (complete *P* handedness induction). After DMF removal, compound **4** was lyophilized twice in the presence of 0.1 M HCl as anion exchange treatment.

Compound **4**, (HCl salt) was isolated in 2% overall yield (3 mg, 0.4 µmol) as a yellow solid according to the SPFS initial scale (*i.e.* 20 µmol).

^{1}H NMR (500 MHz, DMF-d_7) δ = 11.26 (s, 2H), 11.11 (s, 2H), 10.97 (s, 2H), 10.37 (s, 2H), 10.24 (s, 2H), 10.15 (s, 2H), 10.03 (s, 4H), 9.99 (s, 2H), 9.95 (s, 2H), 9.72 (s, 2H), 9.60 (s, 2H), 8.41 (s, 2H), 8.33 (s, 5H), 8.06 (s, 6H), 8.03 (s, 65H), 7.89 (d, *J* = 8.0 Hz, 3H), 7.74 – 7.63 (m, 9H), 7.63 – 7.56 (m, 4H), 7.51 (dd, *J* = 16.9, 6.6 Hz, 12H), 7.40 (dt, *J* = 22.8, 9.0 Hz, 11H), 7.26 (dd, *J* = 20.9, 11.5 Hz, 13H), 7.07 (s, 4H), 6.96 (s, 11H), 6.89 (d, *J* = 7.3 Hz, 1H), 6.87 – 6.75 (m, 19H), 6.72 (d, *J* = 7.8 Hz, 4H), 6.69 (s, 5H), 6.54 (s, 5H), 6.48 (d, *J* = 8.2 Hz, 3H), 6.35 (d, *J* = 7.0 Hz, 6H), 6.25 (s, 3H), 6.18 (t, *J* = 13.0 Hz, 12H), 6.03 (s, 6H), 5.95 (s, 3H), 5.85 (s, 3H), 5.77 (d, *J* = 8.6 Hz, 3H), 5.70 (s, 3H), 5.61 (s, 2H), 4.60 (s, 2H), 4.26 (s, 34H), 3.89 – 3.84 (m, 3H), 3.82 – 3.72 (m, 8H), 3.69 (d, *J* = 4.8 Hz, 4H), 3.67 – 3.58 (m, 20H), 3.54 (d, *J* = 9.4 Hz, 17H), 3.47 (s, 9H), 3.46 – 3.34 (m, 28H), 3.19 (d, *J* = 12.5 Hz, 5H), 3.03 – 2.96 (m, 63H), 2.87 – 2.79 (m, 66H), 2.60 (s, 8H), 2.39 (s, 25H), 2.31 (d, *J* = 6.9 Hz, 2H), 2.22 (s, 6H), 1.59 (s, 2H), 1.33 (d, *J* = 7.4 Hz, 4H), 1.30 (s, 9H), 0.90 (t, *J* = 7.2 Hz, 2H), -0.78 (d, *J* = 6.4 Hz, 6H).

HRMS (ESI$^+$) *m/z* calculated for $C_{385}H_{365}N_{69}O_{82}S_8$ $[M+4H]^{4+}$ 1882.6173; found 1882.6187 $[M+4H]^{4+}$.

1.1.4.7 Synthesis of **5**

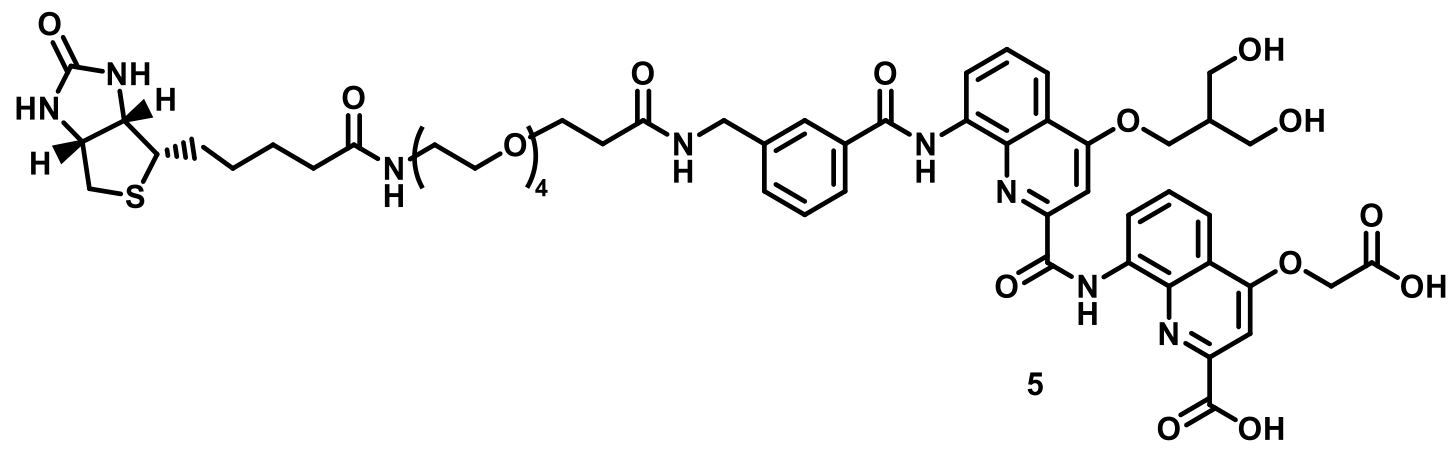

5

Compound 5: Compound **5** was synthesized on Wang resin (1 $mmol.g^{-1}$, 30 µmol scale). After TBAF deprotection, TFA cleavage and purification by preparative RP-HPLC compound **5** was obtained as a yellow powder (13.4 mg, 12.0 µmol, 40%).

^{1}H NMR (500 MHz, CD_3CN/H_2O (1:1, vol/vol): δ = (ppm) 11.90 (s, 1H), 10.31 (s, 1H), 8.51 – 8.40 (m, 2H), 7.80 (d, *J* = 8.9 Hz, 1H), 7.71 – 7.35 (m, 7H), 7.32 (s, 1H), 7.07 – 6.97 (m, 2H), 6.56 (t, *J* = 8.1 Hz, 1H), 3.81 – 3.75 (m, 1H), 3.61 (t, *J* = 13.0, 6.4 Hz, 1H), 3.51 – 3.34 (m, 12H), 3.17 (p, *J* = 5.5 Hz, 3H), 3.09 – 2.99 (m, 2H), 2.75 (dd, *J* = 13.1, 5.0 Hz, 1H), 2.57 (d, *J* = 13.1 Hz, 1H), 2.36 (t, *J* = 15.6, 6.1 Hz, 3H), 2.27 (p, *J* = 5.8 Hz, 1H), 2.02 (t, *J* = 7.8 Hz, 2H), 1.51 (m, *J* = 9.5, 6.5 Hz, 1H), 1.46 – 1.32 (m, 3H), 1.24 – 1.13 (m, 3H).

HRMS (ESI^{+}) *m/z* calculated for $C_{55}H_{67}N_8O_{17}S_1$ 1143.4339 $[M+H]^+$; found 1143.4273 $[M+H]^+$.

1.1.4.8 Synthesis of **6**

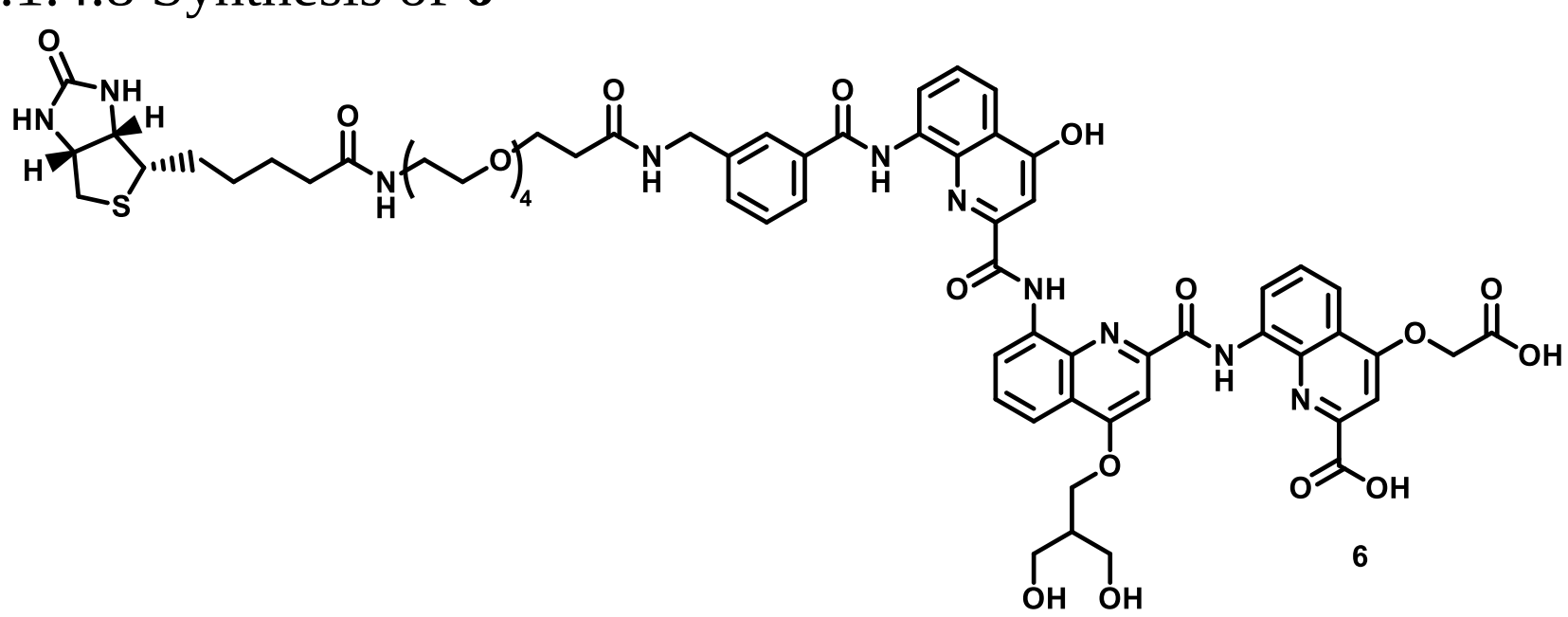

6

Compound 6: Oligomer **6** was synthesized on Wang resin (1 $mmol.g^{-1}$, 20 µmol scale). After TBAF deprotection, TFA cleavage, lyophilization and purification by preparative RP-HPLC, compound **6** was obtained as a yellow powder in 18% yield (4.70 mg, 3.60 µmol).

^{1}H NMR (500 MHz, CD_3CN/H_2O (1:1, vol/vol)): δ = 11.85 (s, 1H), 11.55 (s, 1H), 9.85 (s, 1H), 8.47 (d, *J* = 8.0 Hz, 1H), 7.89 (d, *J* = 8.4 Hz, 1H), 7.80 (d, *J* = 8.9 Hz, 1H), 7.77 – 7.62 (m, 4H), 7.54 – 7.42 (m, 5H), 7.28 – 7.18 (m, 4H), 7.15 – 7.08 (m, 2H), 6.62 (t, *J* = 8.3 Hz, 1H), 6.29 (s, 1H), 3.80 (d, *J* = 6.4 Hz, 1H), 3.63– 3.53 (m, 2H), 3.47 – 3.31 (m, 13H), 3.14 (q, *J* = 5.5 Hz, 2H), 3.11 – 2.97 (m, 2H), 2.75 (dd, *J* = 13.1, 5.0 Hz, 1H), 2.57 (d, *J* = 13.2 Hz, 1H), 2.34 (m, 3H), 2.29 (p, *J* = 5.9 Hz, 1H), 2.00 (t, *J* = 7.8 Hz, 3H), 1.56 – 1.44 (m, 1H), 1.44 – 1.31 (m, 3H), 1.22 – 1.13 (m, 3H).

HRMS (ESI^{+}) *m/z* calculated for $C_{65}H_{73}N_{10}O_{19}S_1$ 1329.4769 $[M+H]^+$; found 1329.4683 $[M+H]^+$.

1.1.4.9 Synthesis of **7**

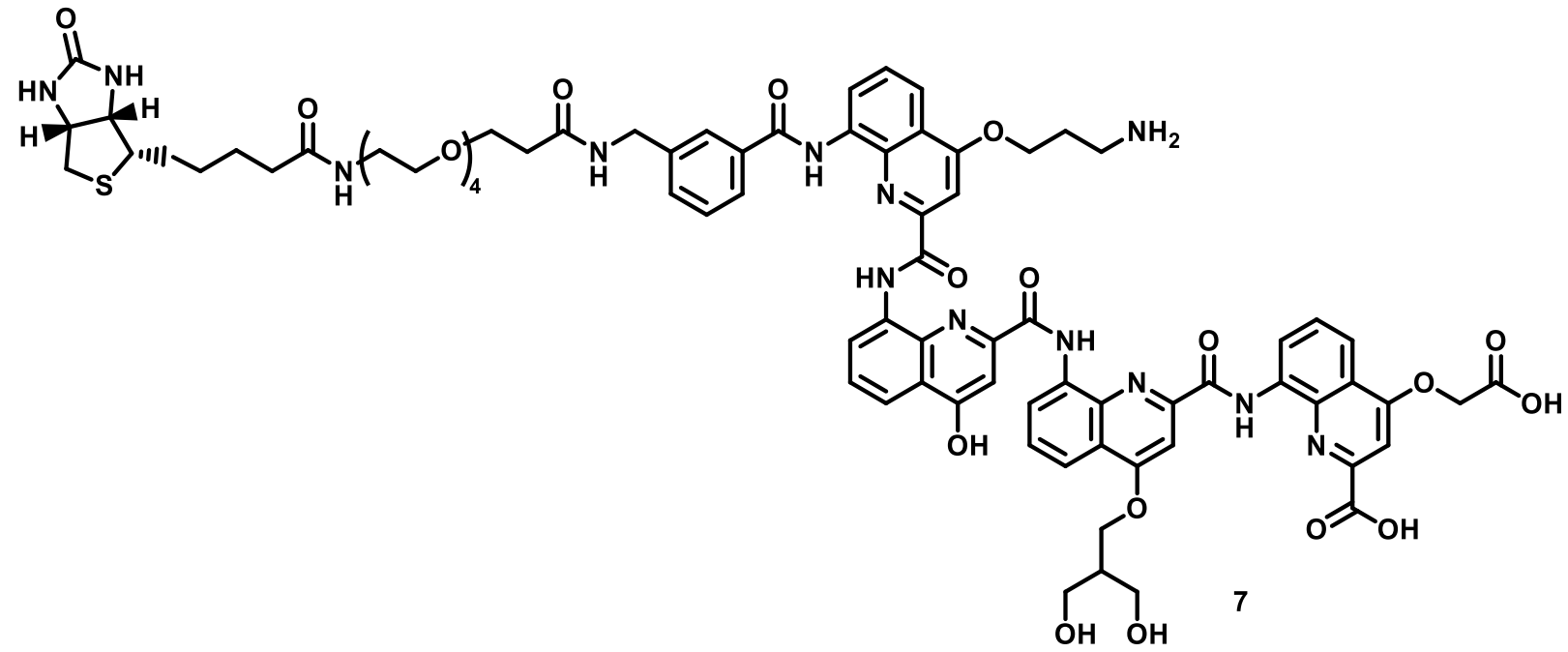

7

Compound 7: Oligomer **7** was synthesized on LL-Wang resin (0.43 mmol.g-1, 20 µmol scale). After TBAF deprotection, TFA cleavage, lyophilization and purification by preparative RP-HPLC, compound was obtained as a yellow powder in 28% yield (8.78 mg, 5.58 µmol).

^{1}H NMR (500 MHz, CD_3CN/H_2O (1:1, vol/vol)): δ = 11.48 (s, 1H), 11.37 (s, 1H), 11.29 (s, 1H), 9.63 (s, 1H), 8.04 (s, 1H), 7.89 – 7.67 (m, 7H), 7.61 (m, 3H), 7.56 – 7.46 (m, 3H), 7.38 (m, 1H), 7.30 (s, 1H), 7.28 – 7.04 (s, 7H), 6.58 (td, *J* = 7.9, 3.1 Hz, 1H), 6.30 –6.08 (m, 2H), 6.03 (s, 1H), 3.85 (d, *J* = 6.6 Hz, 1H), 3.62 (t, *J* = 6.5 Hz, 1H), 3.50 – 3.33 (m, 14H), 3.19 – 3.12 (m, 2H), 3.10 – 2.97 (m, 2H), 2.74 (dd, *J* = 13.0, 5.1, 1.7 Hz, 1H), 2.56 (d, *J* = 13.2 Hz, 1H), 2.44 – 2.36 (m, 5H), 2.30 (p, *J* = 5.8 Hz, 1H), 2.04 – 1.97 (m, 2H), 1.54 – 1.46 (m, 1H), 1.44 – 1.30 (m, 5H), 1.23 – 1.11 (m, 4H).

HRMS (ESI$^+$) *m/z* calculated for $C_{78}H_{86}N_{13}O_{21}S_1$ 1572.5776 $[M+H]^+$; found 1572.5862 $[M+H]^+$.

1.1.5 Characterization of new synthetic compounds

1.1.5.1 Compound **1c**

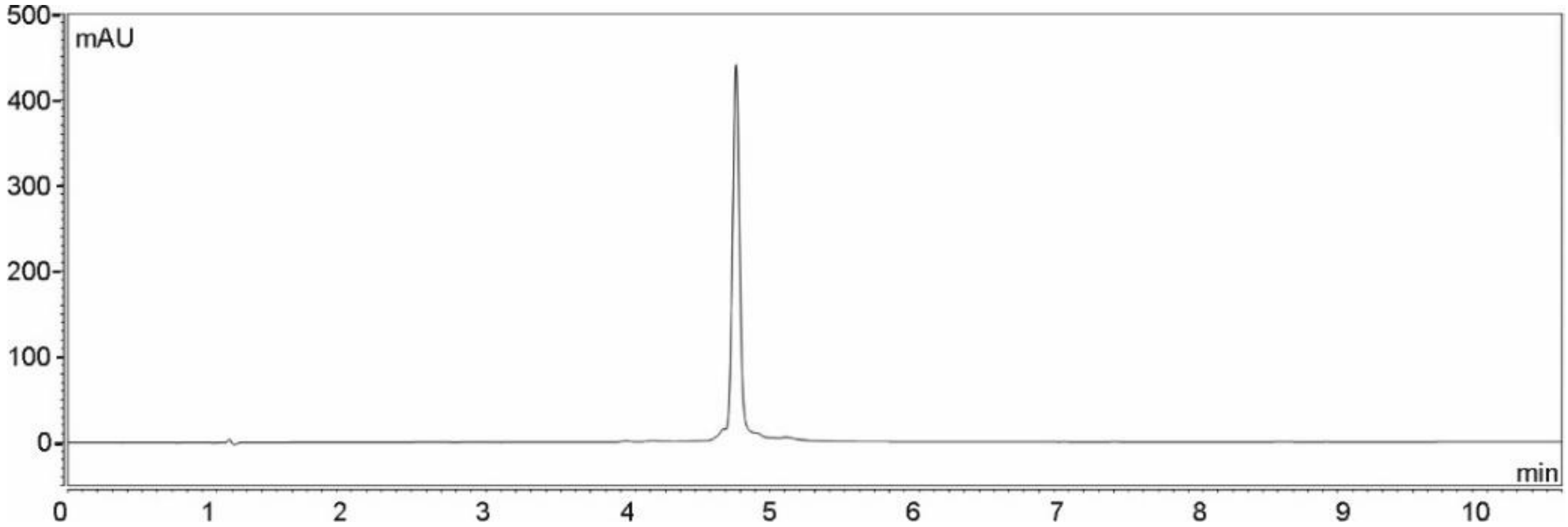

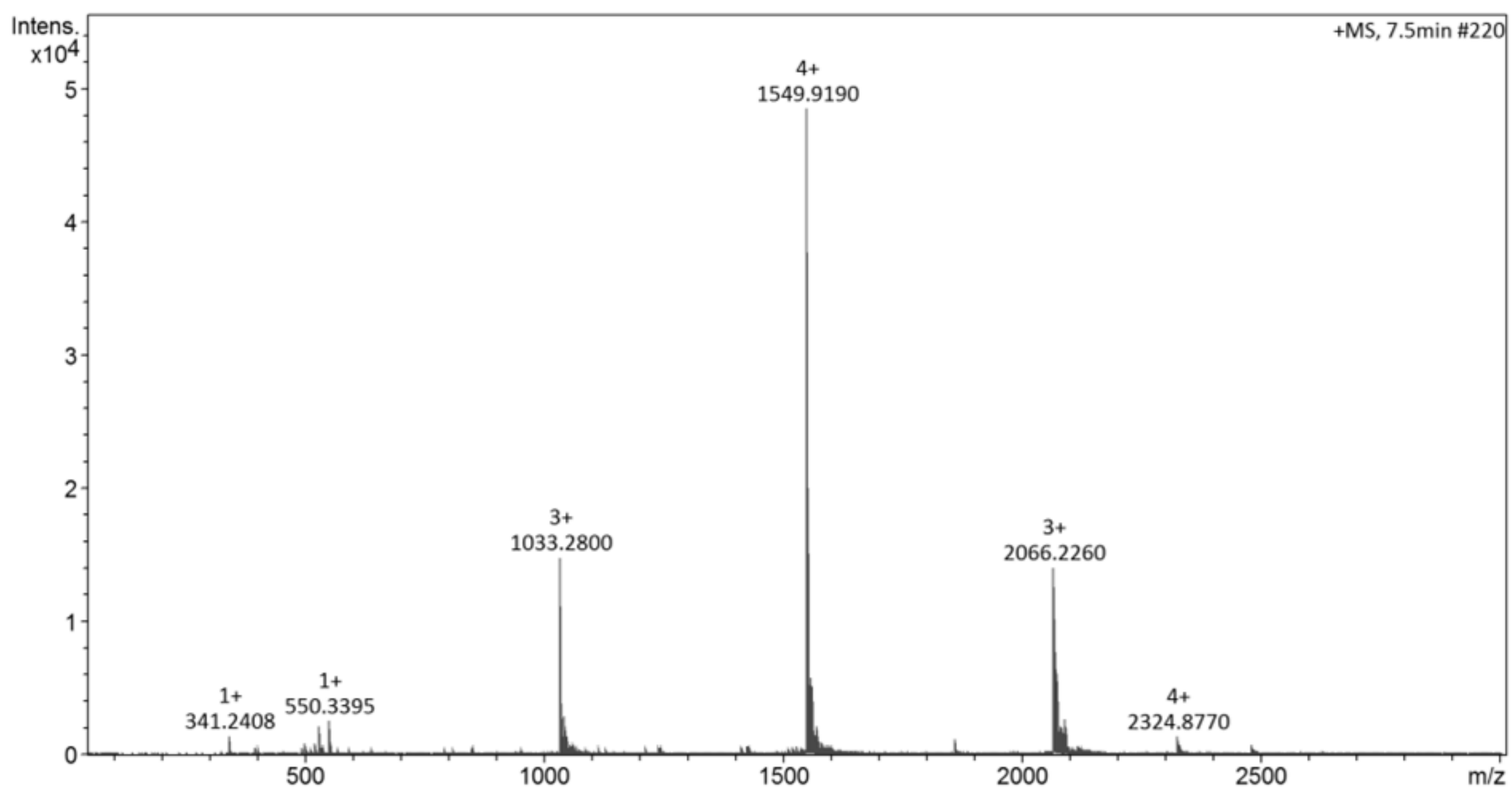

RP-HPLC chromatogram and ESI-MS spectrum of compound **1c.**

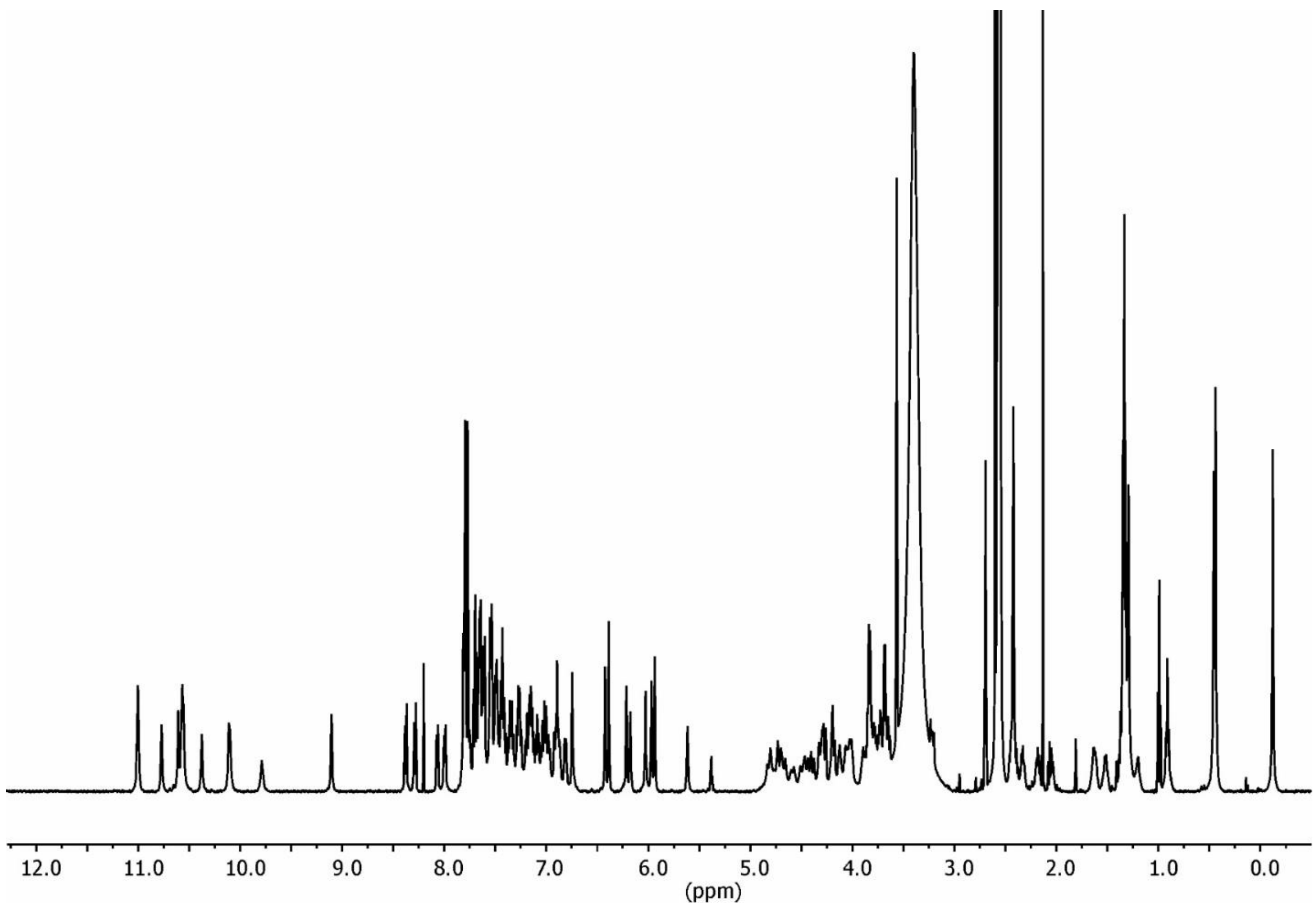

^{1}H NMR spectrum (500 MHz, DMSO-d_6, 25°C) of **1c**.

1.1.5.2 Compound **2a**

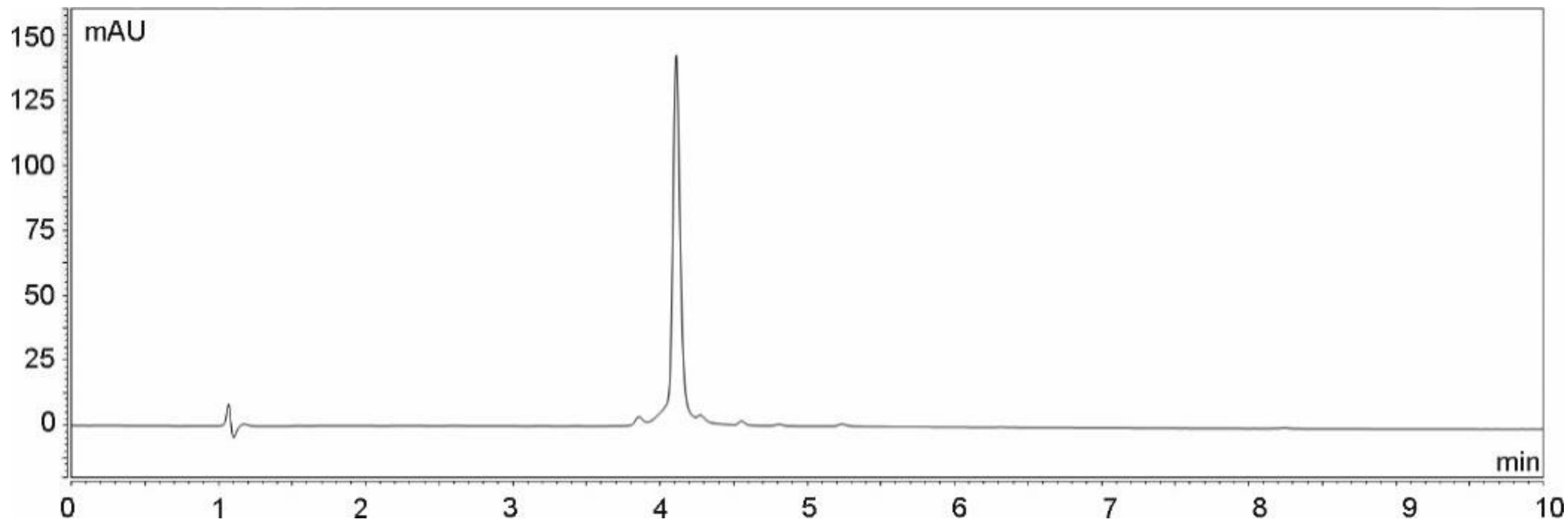

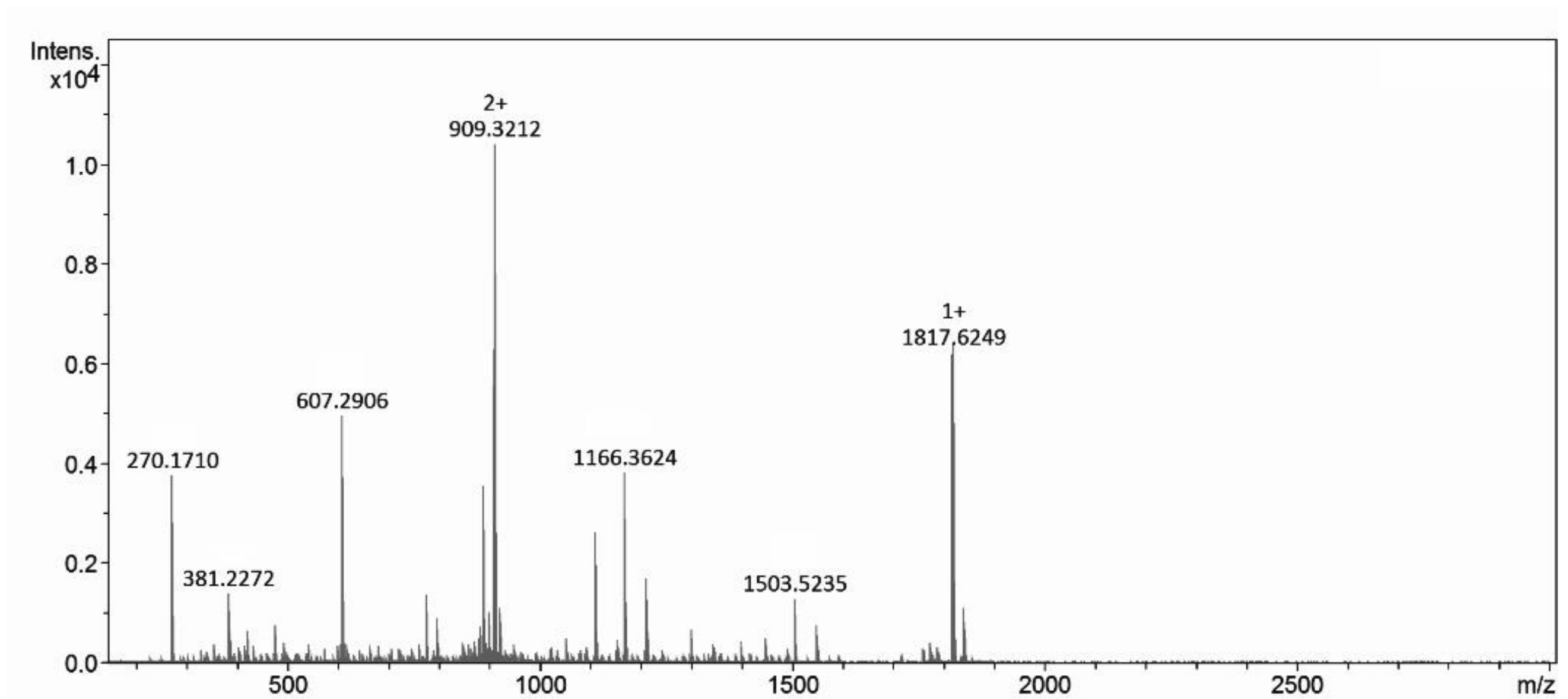

RP-HPLC chromatogram and ESI-MS spectrum of compound **2a**

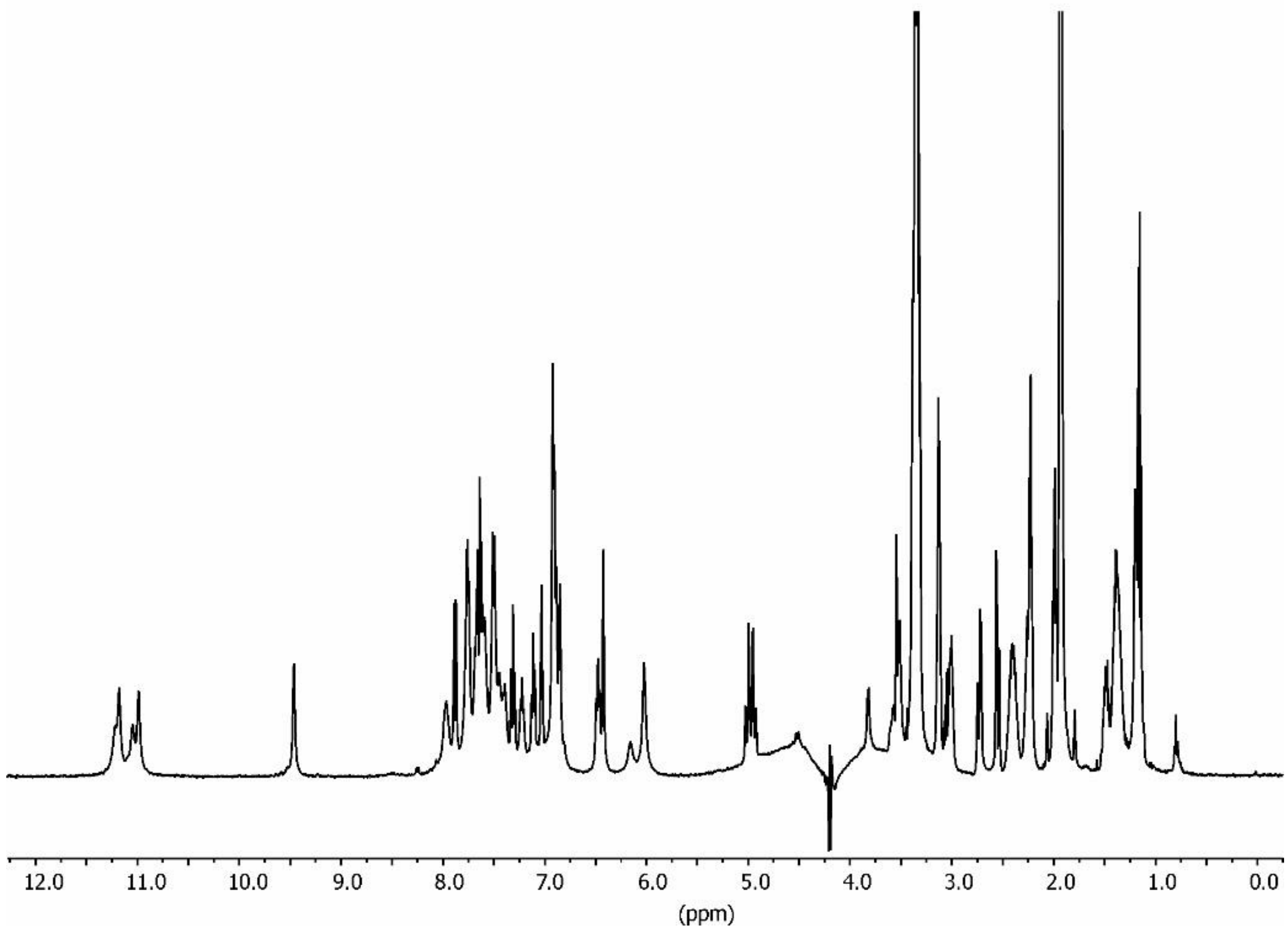

^{1}H NMR spectrum (500 MHz, CD_3CN/H_2O, 25°C) of **2a**.

1.1.5.3 Compound **2b**

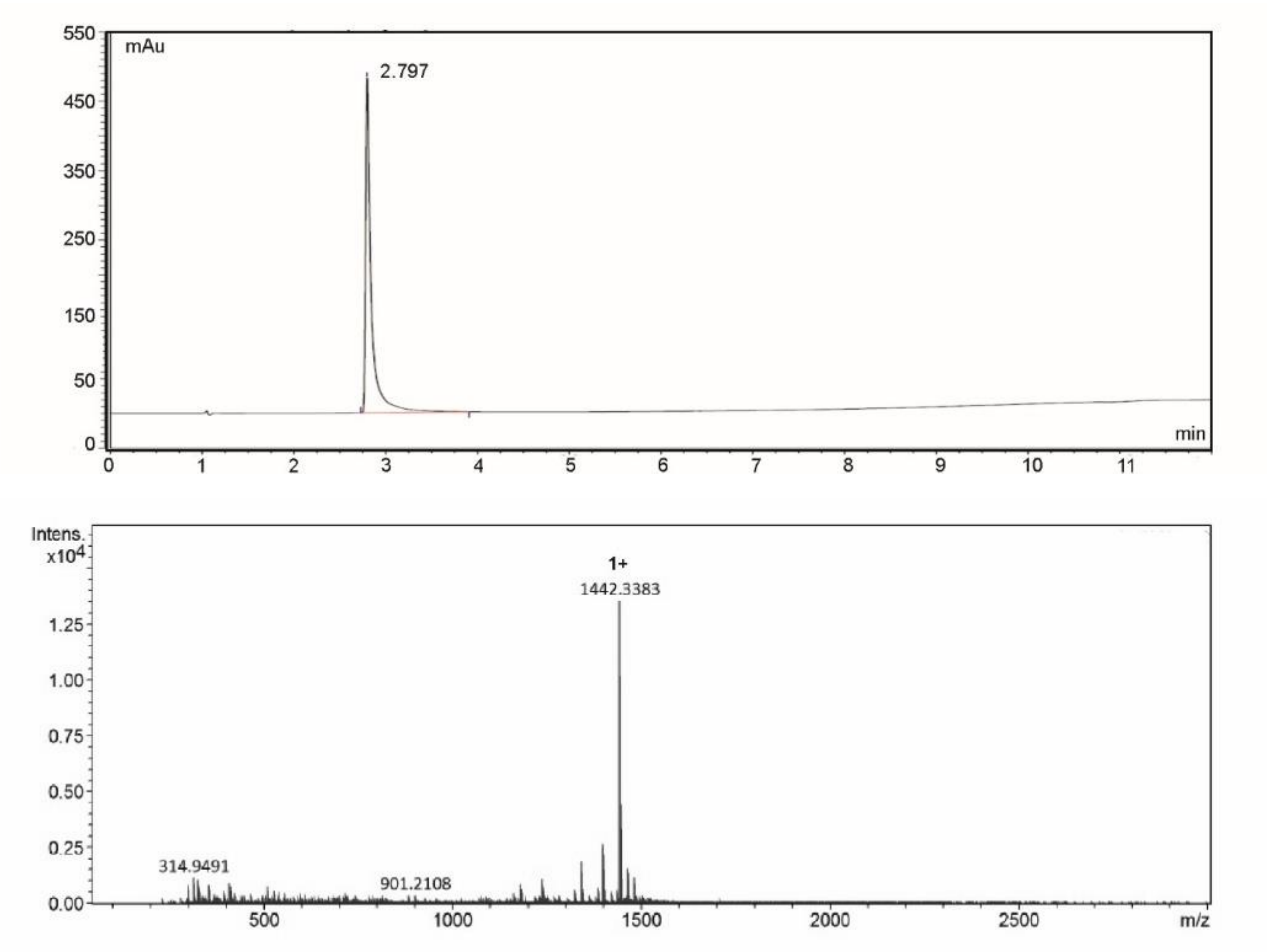

RP-HPLC chromatogram and ESI-MS spectrum of compound **2b**

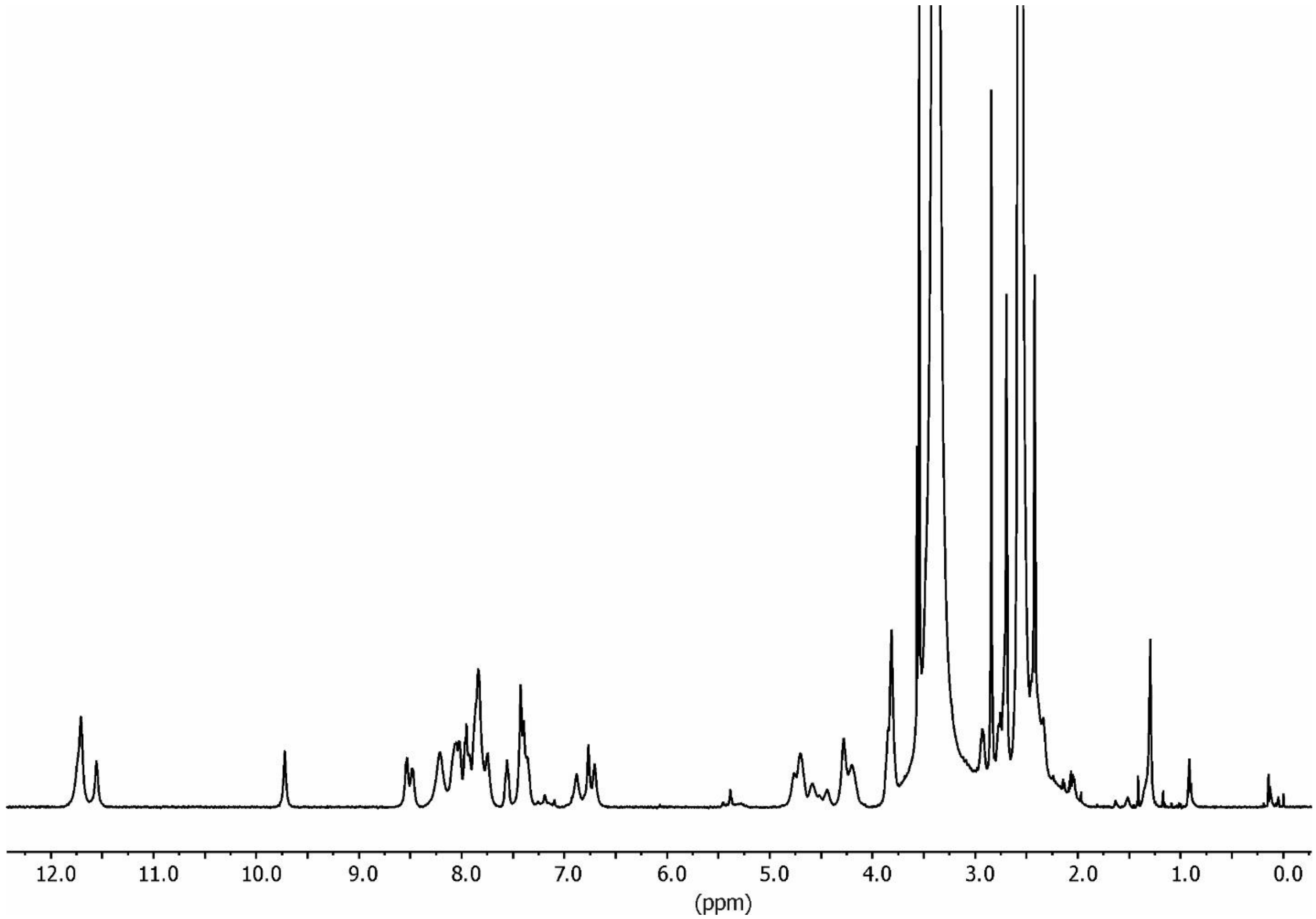

^{1}H NMR spectrum (500 MHz, DMSO-d_6, 25°C) of **2b** after anion (HCl) exchange.

1.1.5.4 Compound **3**

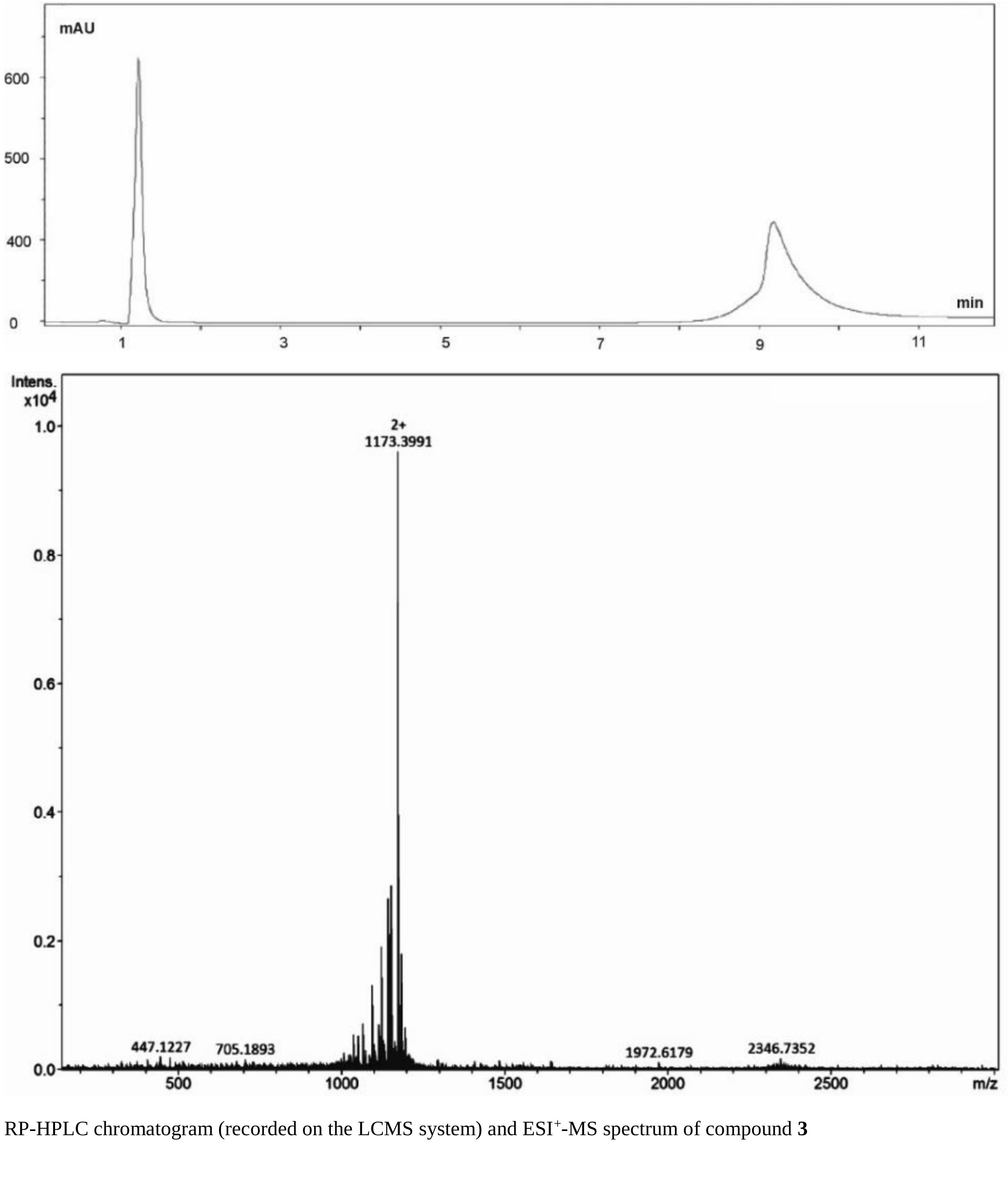

RP-HPLC chromatogram (recorded on the LCMS system) and ESI^{+}-MS spectrum of compound **3**

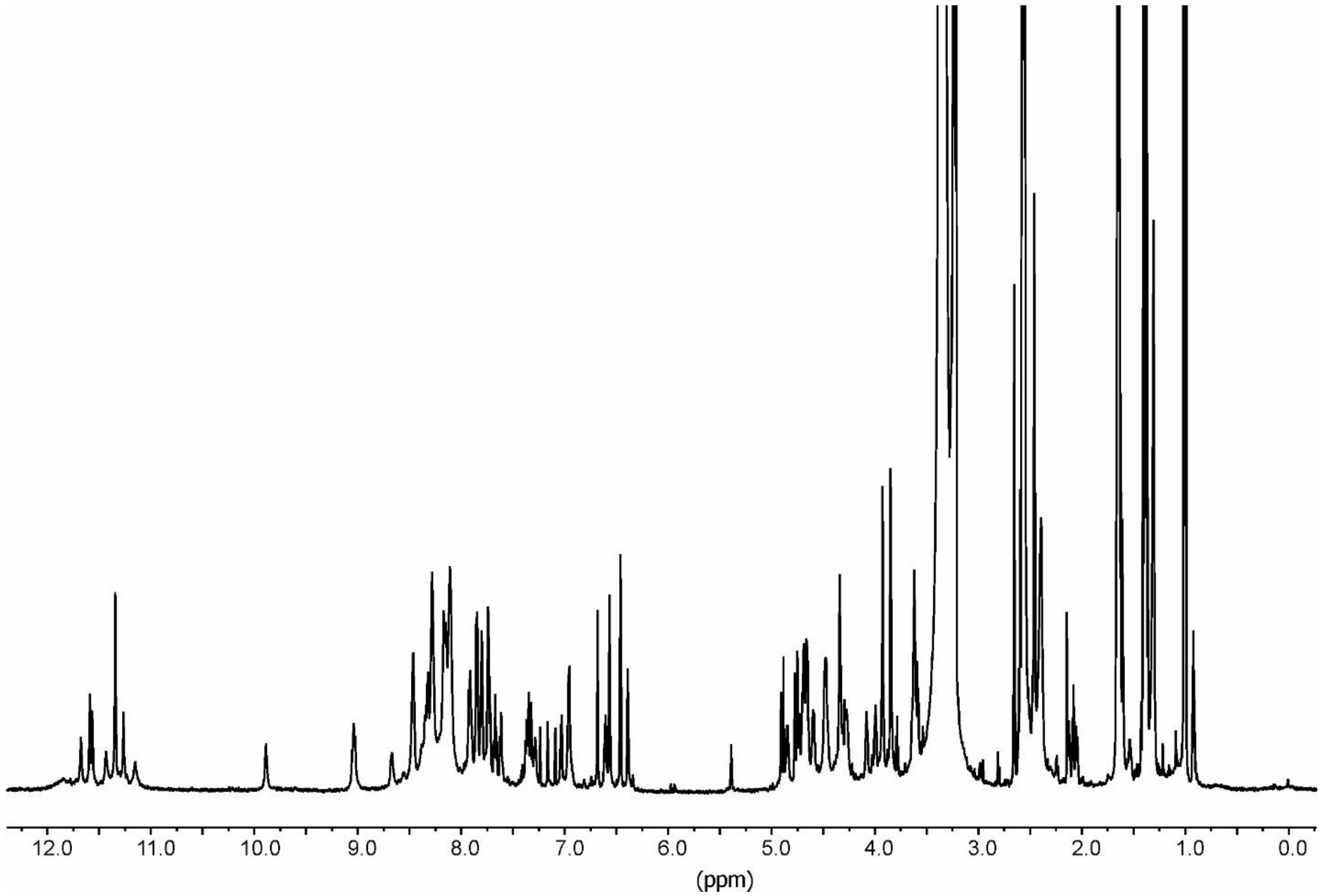

^{1}H NMR spectrum (700 MHz, DMSO-d_6, 25°C) of compound **3** after anion (HCl) exchange.

1.1.5.5 Compound fragment A

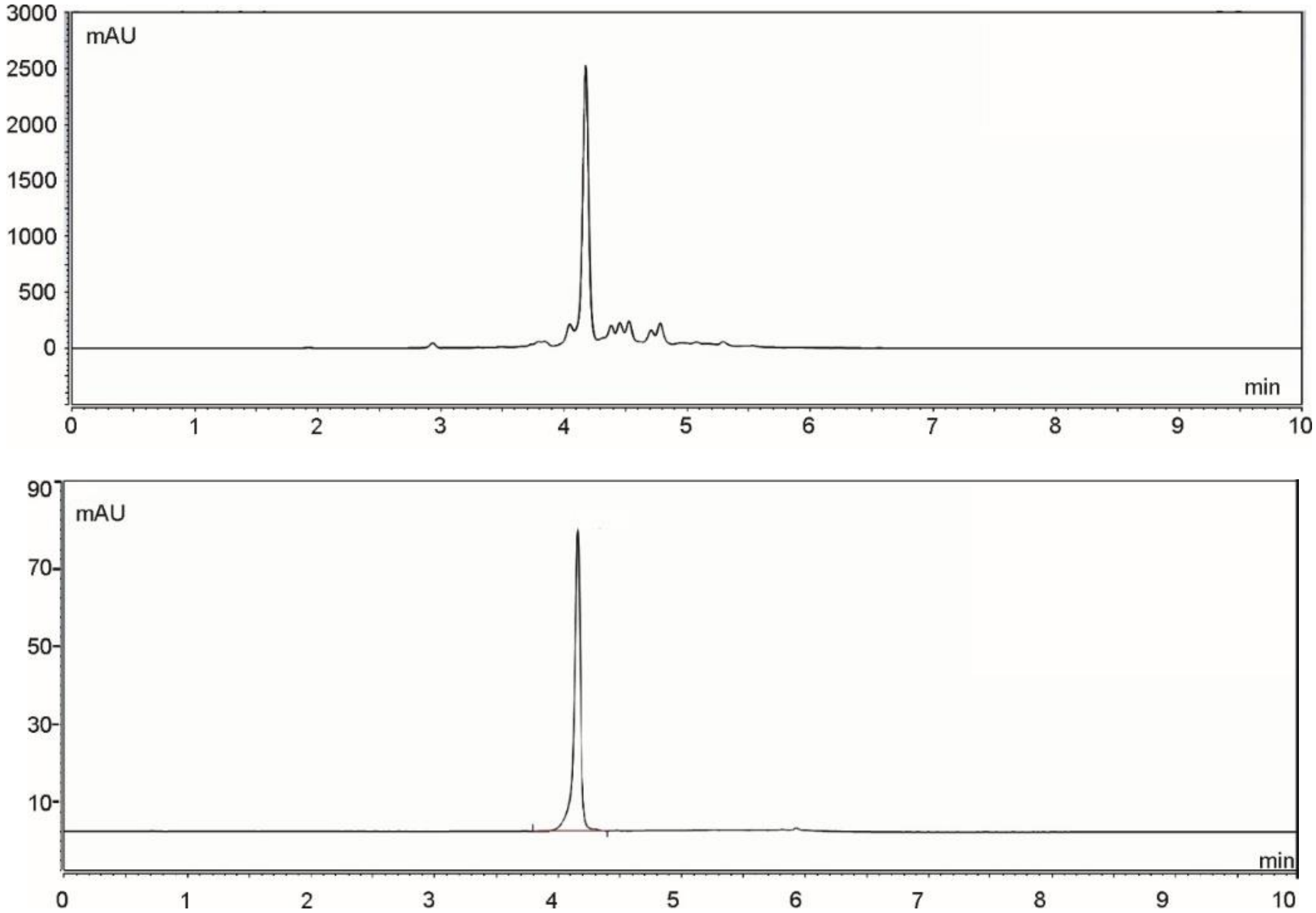

RP-HPLC chromatograms of crude (top) **fragment A** recovered after automated SPFS and pure (bottom) **fragment A** after semi-preparative RP-HPLC purification.

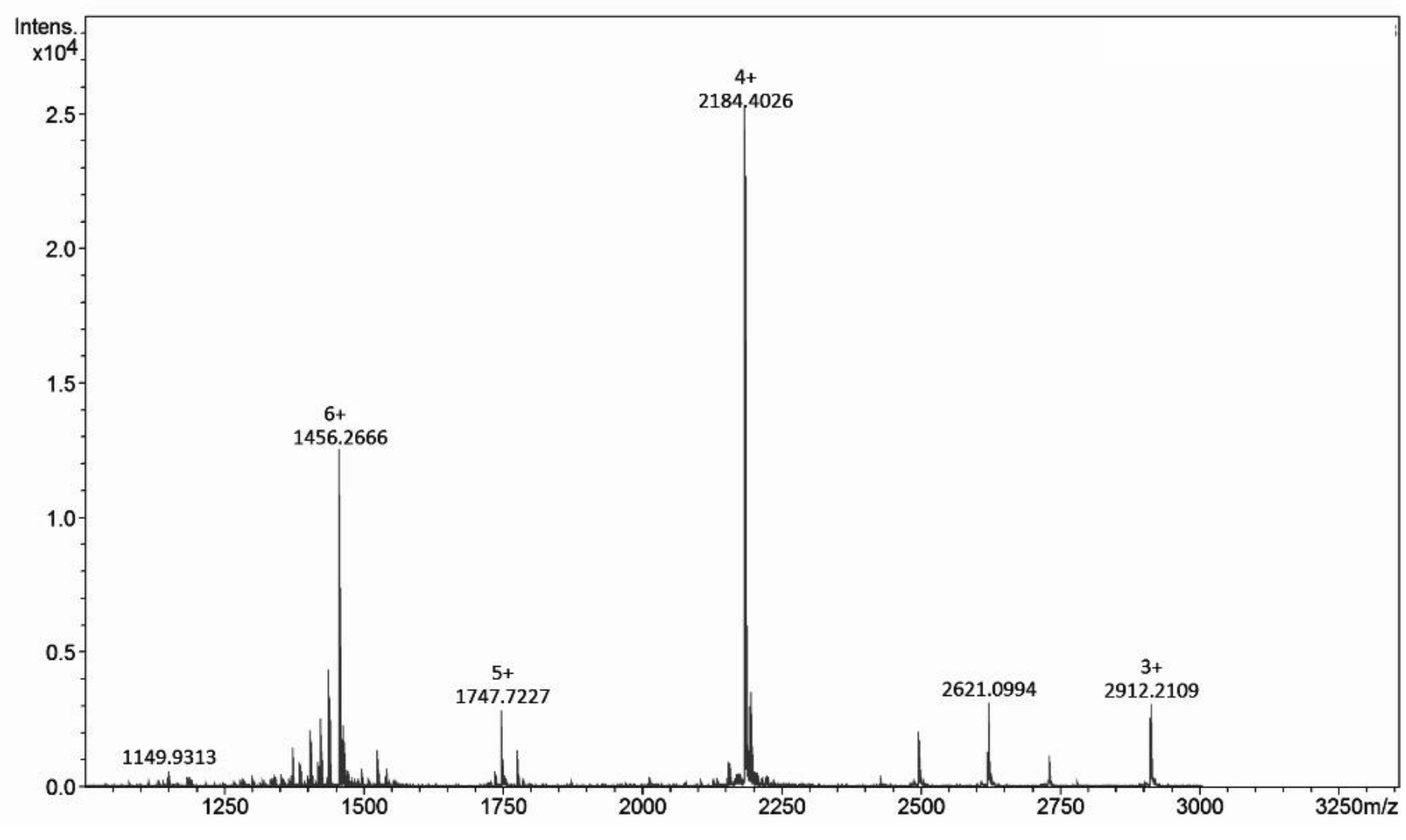

ESI^{+}-MS spectrum of purified **fragment A.**

1.1.5.6 Compound **4**

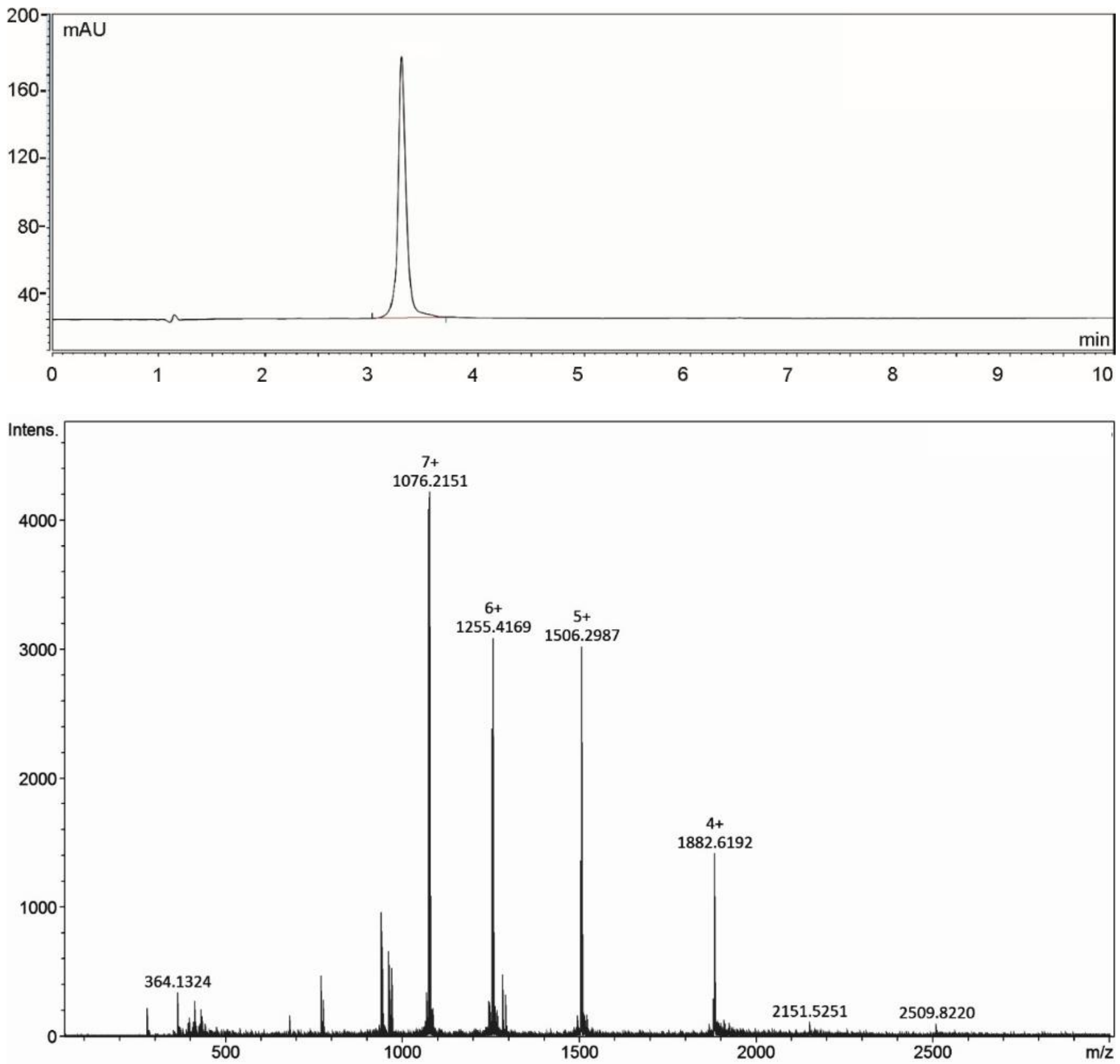

RP-HPLC chromatogram after semi-preparative purification and anion (HCl) exchange and ESI$^+$-MS spectrum of **4.**

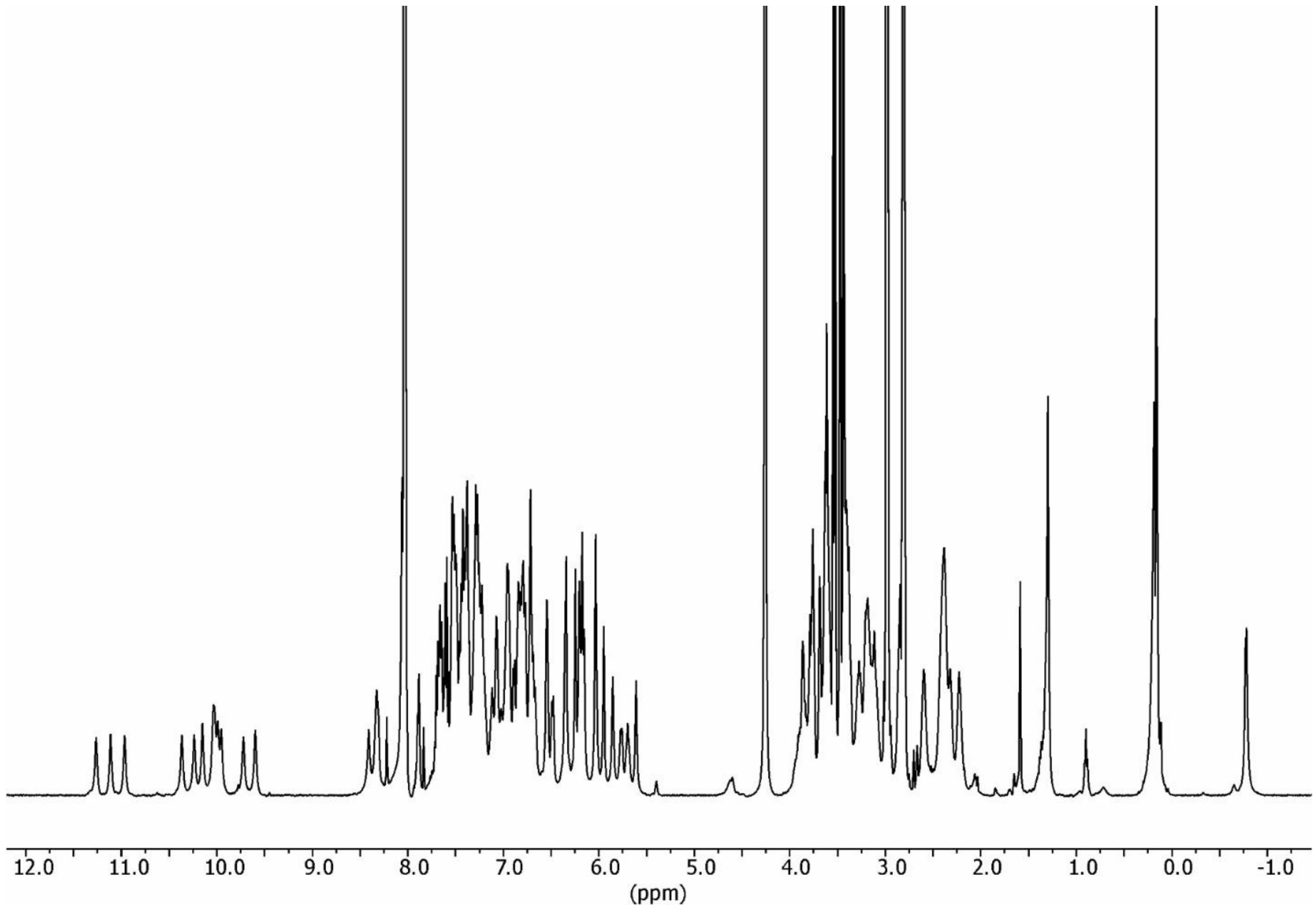

^{1}H NMR spectrum (500 MHz, DMF-d_7, 25°C) of compound **4.**

1.1.5.7 Compound **5**

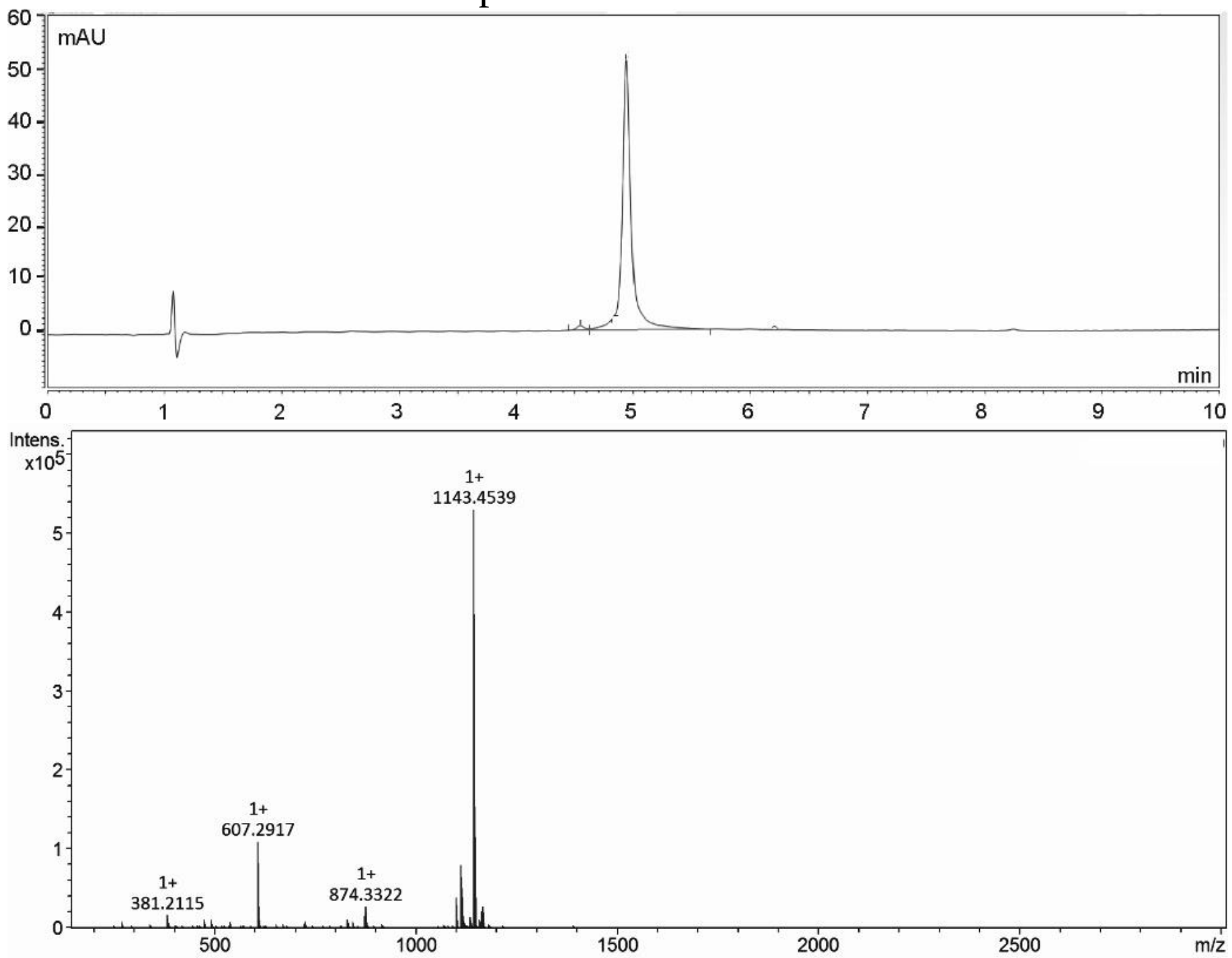

RP-HPLC chromatogram and ESI^{+}-MS spectrum of compound **5**

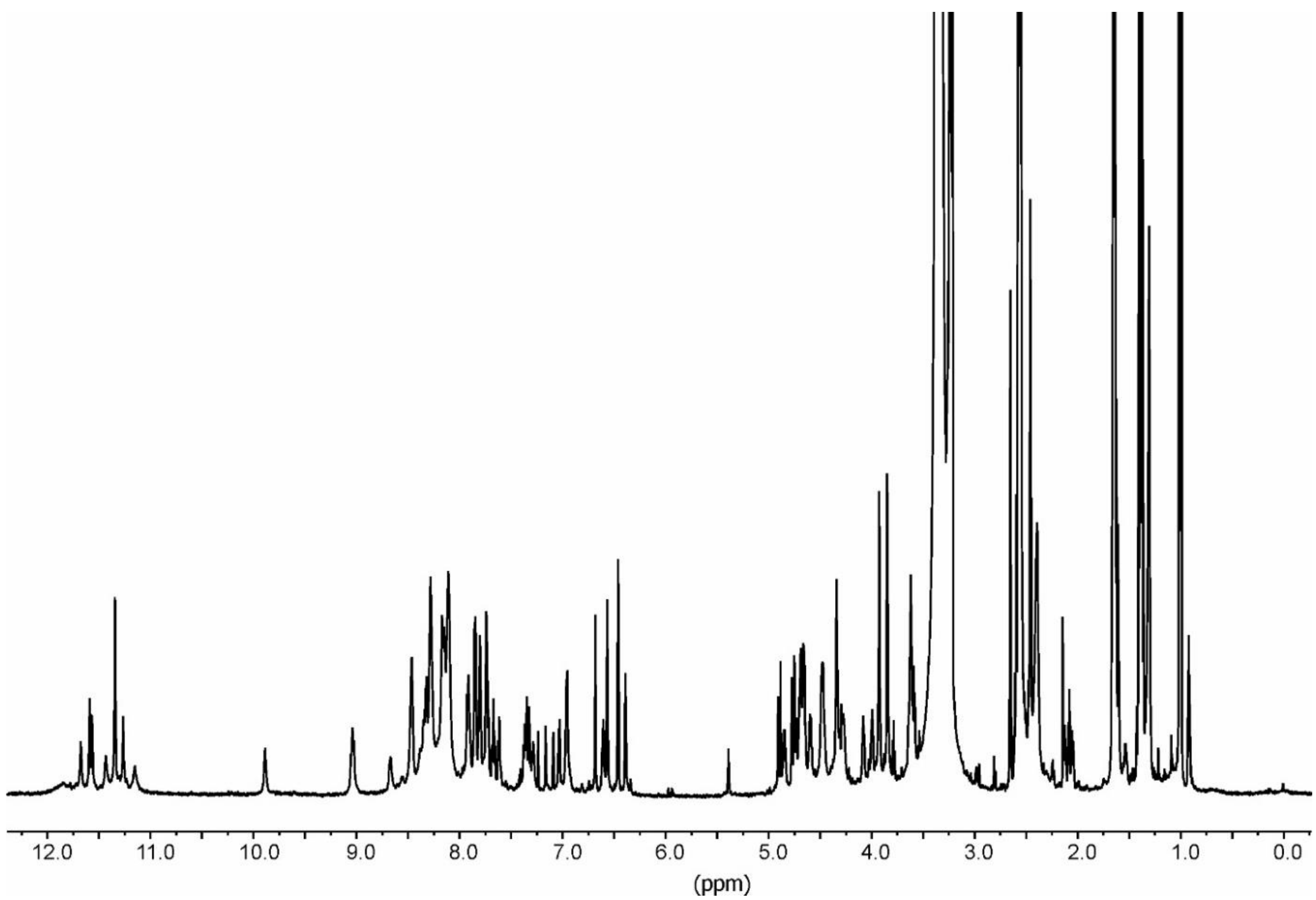

^{1}H NMR spectrum (500 MHz, CD_3CN/H_2O, 25°C) of compound **5.**

1.1.5.8 Compound **6**

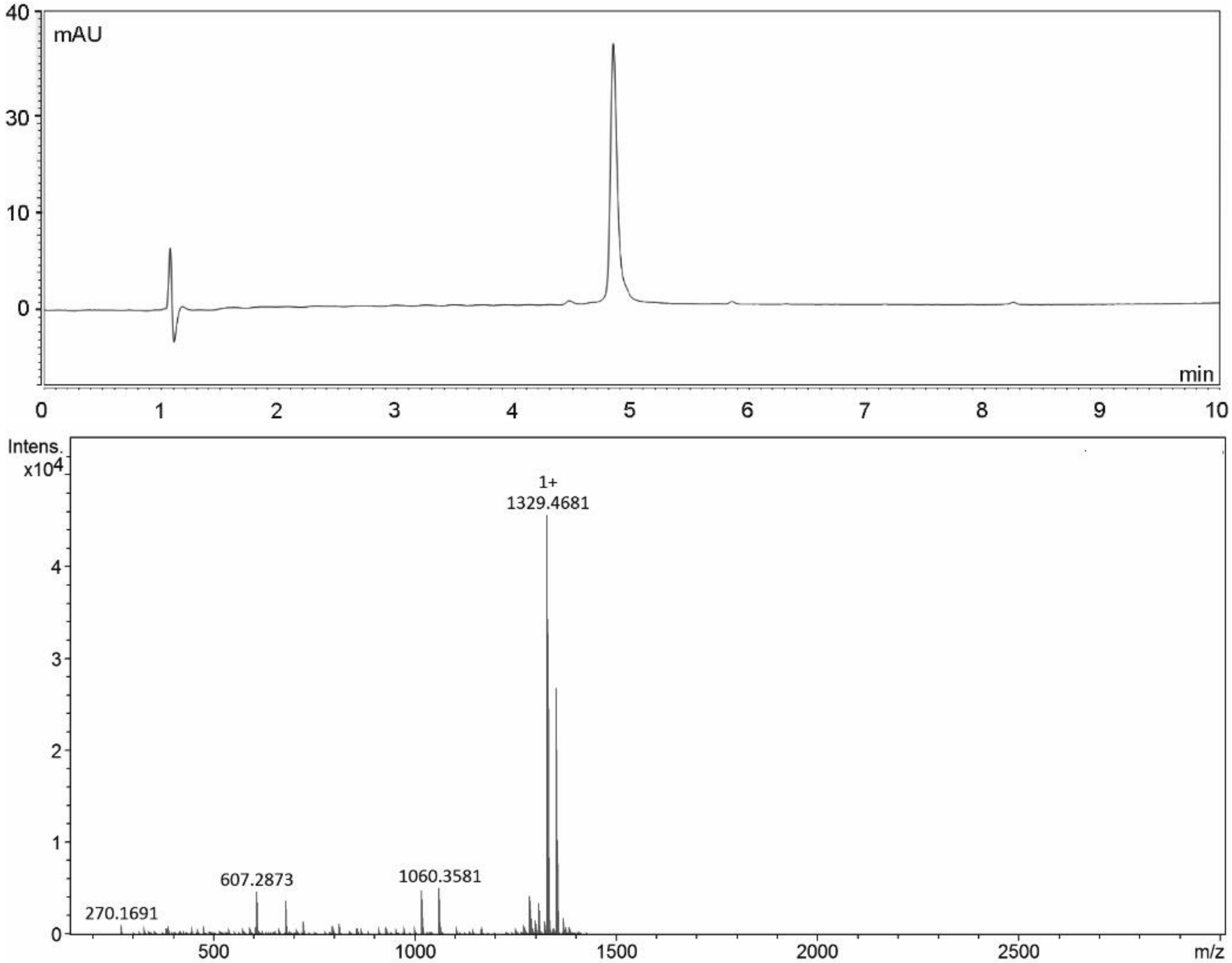

RP-HPLC chromatogram and ESI^{+}-MS spectrum of compound **6**

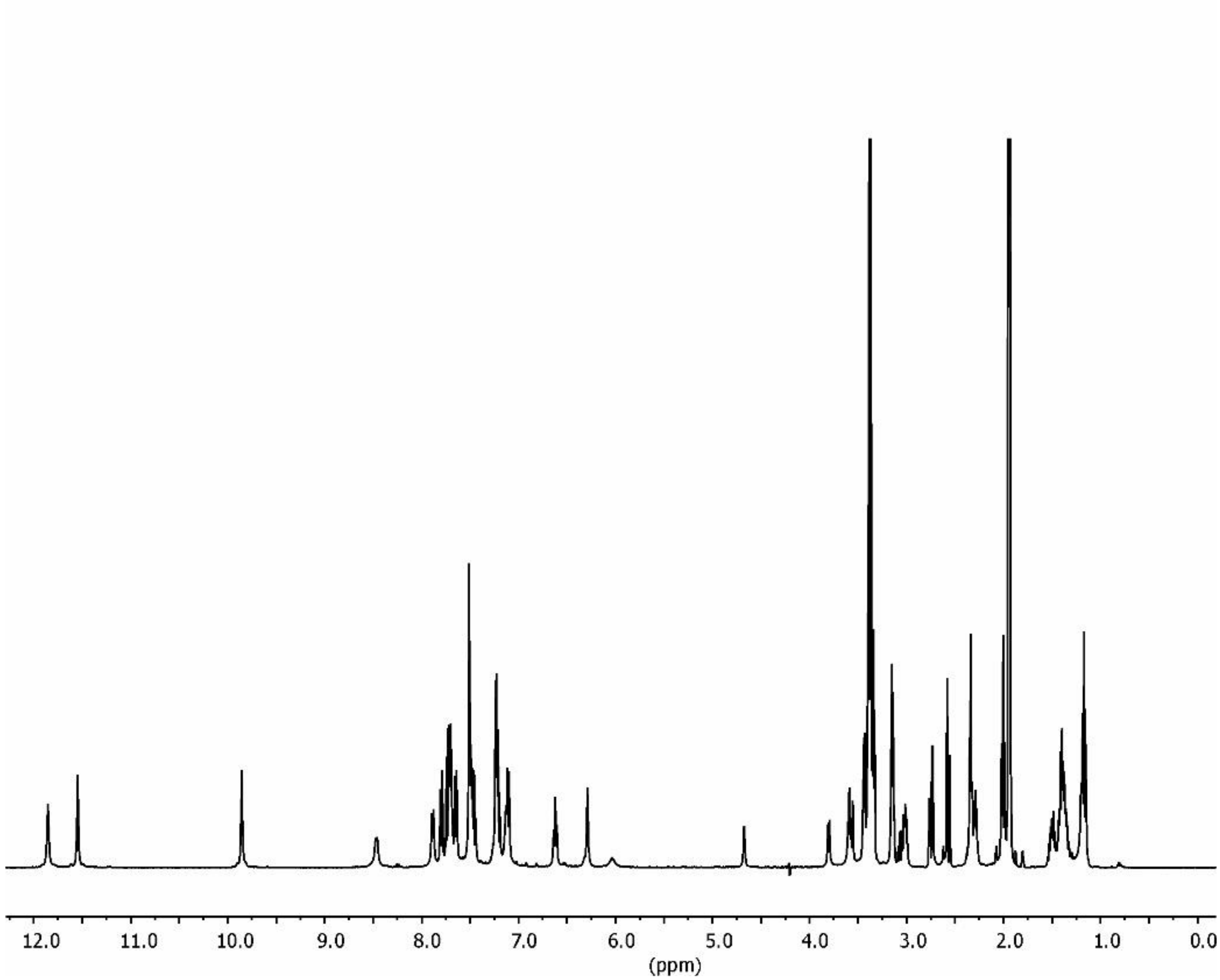

^{1}H NMR spectrum (500 MHz, CD_3CN/H_2O, 25°C) of compound **6.**

1.1.5.9 Compound 7

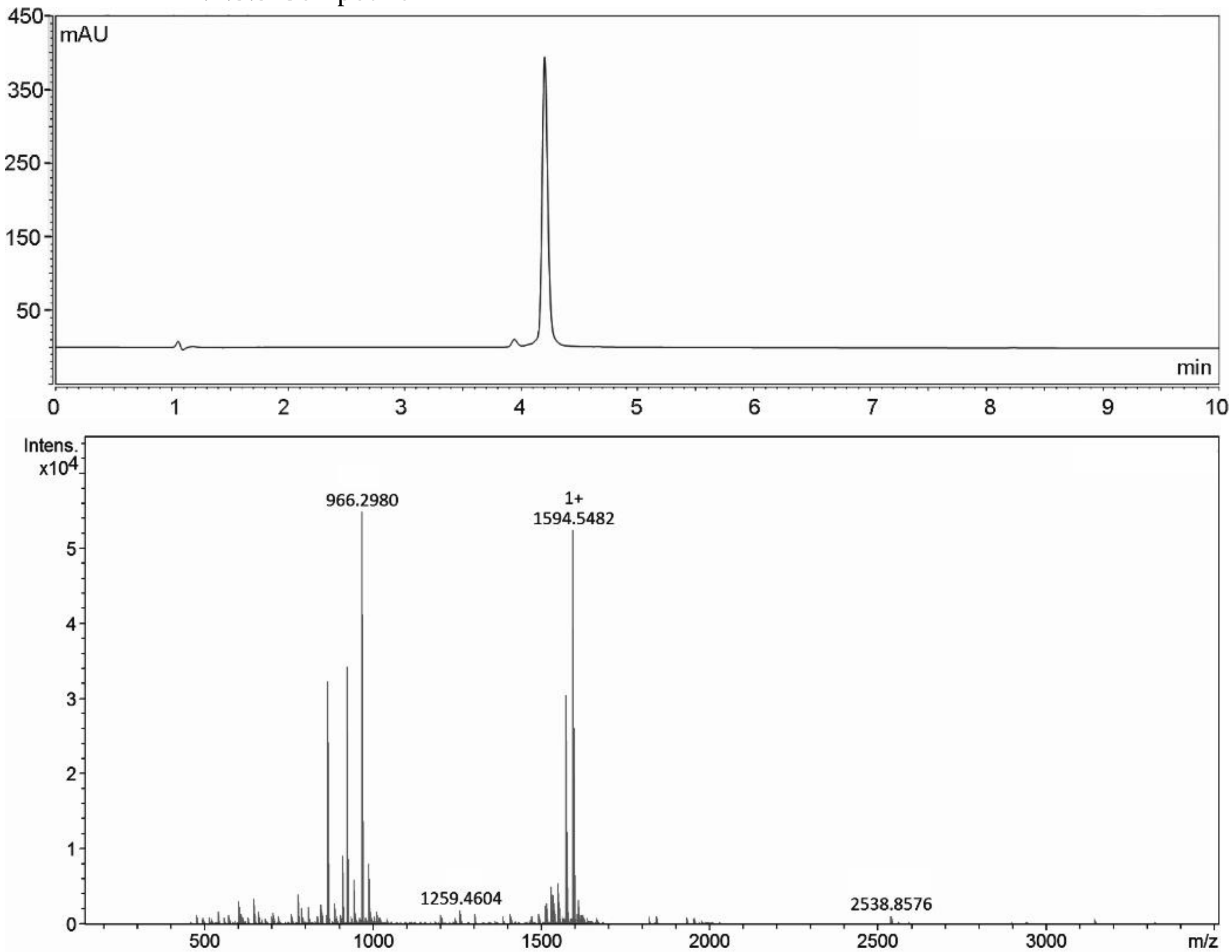

RP-HPLC chromatogram and ESI^{+}-MS spectrum of compound **7.**

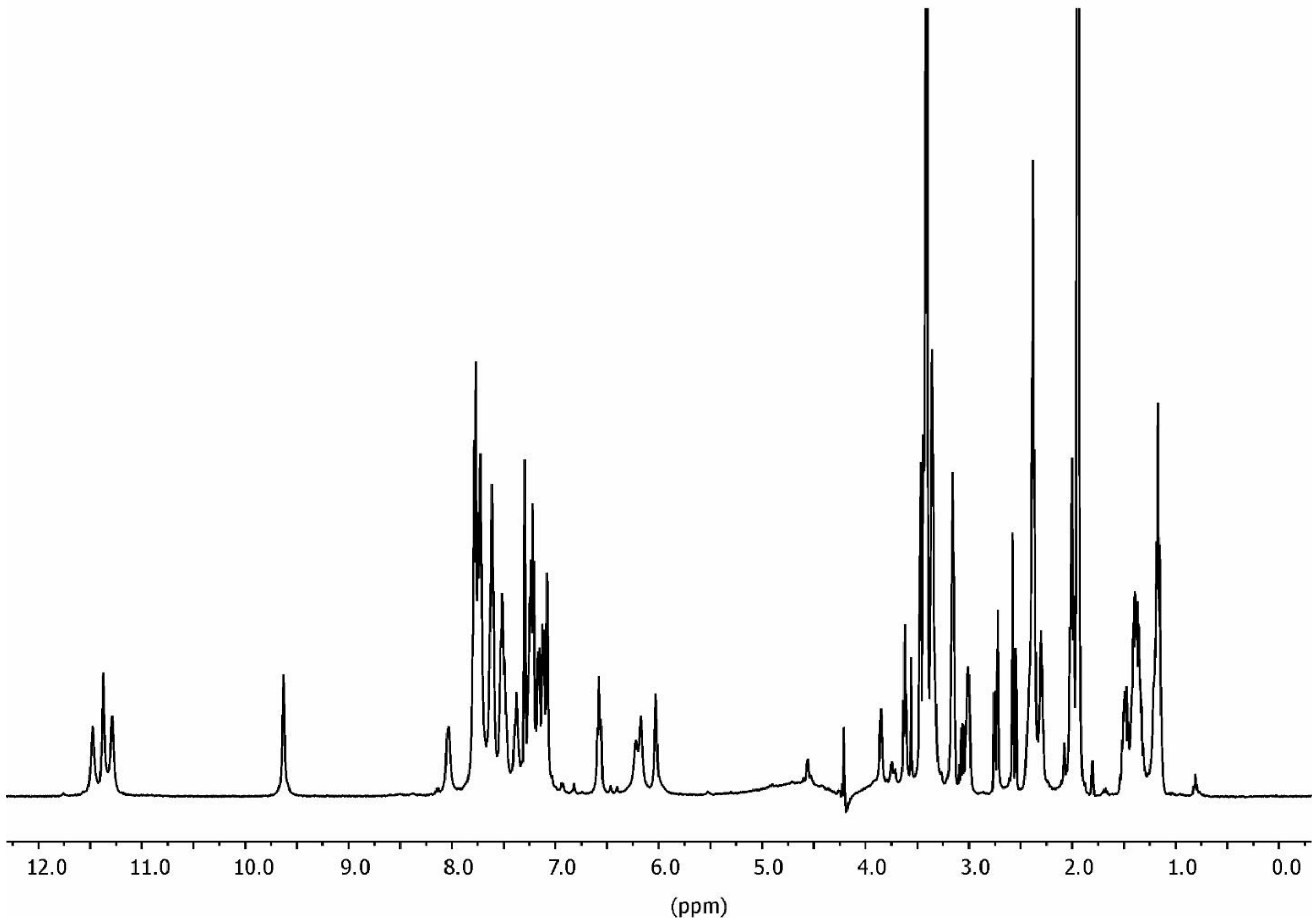

^{1}H NMR spectrum (500 MHz, CD_3CN/H_2O, 2 °C) of compound **7.**

1.2 Biochemistry

1.2.1 Generation and identification of Nanofitin C10

The combinatorial library of Nanofitins for ribosome display was prepared as previously described[32]. Briefly, the library was assembled by two successive overlapping PCRs from degenerated oligonucleotides encoding NNS triplets (N = A, C, T or G and S = C or G). Eventually, a final PCR step added the 5'- and 3'-flanking regions necessary for ribosome display. The PCR-amplified library was transcribed and the selection was performed at 4°C against 15 pmol of the immobilized biotinylated foldamer **1a**. Four rounds of selection were performed to isolate high-affinity binders. The pressure of selection was adjusted by gradually increasing the time in wash-steps during the 4 first rounds (8 washes of 30 s, 3 min and 15 min, respectively, for rounds 1, 2, and 3, then 4 washes of 15 min followed with 4 washes of 30 min for round 4).

Amplified DNA material from the fourth round was cloned between BamHI and HindIII restriction sites of a plasmid derived from pQE-30 (*Qiagen*), and the ligation mixture was transformed into *E. coli* DH5α LacIq strains (*Invitrogen*). Clones selected on 2xYT medium plates containing 100 µg/ml ampicillin and 25 µg/ml kanamycin were inoculated into a deep-well plate containing 0.75 ml of 2xYT medium with 100 µg/ml ampicillin, 25 µg/ml kanamycin, and 1% glucose in each well. After growing overnight cultures at 37°C under shaking at 600 rpm, 0.2 ml of each culture were used to inoculate another deep-well plate containing 1.25 ml of 2xYT medium supplemented with 100 µg/ml ampicillin, 25 µg/ml kanamycin, and 0.1% glucose per well. The plate was incubated at 37°C for 3 h under shaking at 600 rpm. Expression of the Nanofitin clones was induced by addition of 50 µl of Isopropyl β-D-1-thiogalactopyranoside at a final concentration of 0.5 mM and incubation at 30°C for 4 h under shaking at 600 rpm. Cells were pelleted by centrifugation (20 min at 2000g), and supernatants were discarded. Proteins were extracted with 100 µl of 1X BugBuster Protein Extraction Reagent (*Novagen*) per well with shaking for 1 h at room temperature, and 350 µl of TBS (20 mM Tris-HCl, 150 mM NaCl, pH 7.4) were added. Cell debris was pelleted by centrifugation (20 min at 2000 × g) and supernatants were used for screening.

Streptavidin (100 µl, 66 nM; *Sigma-Aldrich*) in TBS was immobilized in Maxisorp plate wells (*Nunc*) by overnight incubation at 4°C. Each of the following steps were run at room temperature, with shaking at 600 rpm for incubation steps. The wells were washed 3 times with 300 µl of TBS, then blocked with 300 µl of 0.5% BSA (bovine serum albumin; *Sigma-Aldrich*) in TBS for 1 h. The plate was flicked over and **1a** (100 µl, 40 nM) in TBS with 0.5% BSA was allowed to bind for 1 h. Prior to each of the following incubation steps, the wells were washed 3 times with 300 µl of TBS containing 0.1% Tween 20. Crude *E. coli* extracts (100 µl, undiluted or diluted 1:10 in TBS with 0.1% Tween 20) were applied to wells with and without immobilized antigen for 1 h. Revelation was then carried over by the addition of 100 µl of RGS His antibody HRP conjugate (*Qiagen*) diluted 1:4000 in TBS with 0.1% Tween 20 for 1 h, followed by the addition of 100 µl of o-Phenylenediamine dihydrochloride substrate (*Sigma-Aldrich*) solution at 1 mg/ml in revelation buffer (0.05 M citric acid, 0.05% hydrogen peroxide). Absorbance at 450 nm was measured using a Varioskan ELISA plate reader (*Thermo Scientific*), revealing Nanofitin C10 as a binder with a strong signal-to-background ratio.

1.2.2 Materials and general methods

All chemicals were purchased from commercial suppliers (*Fischer Scientific*, *Thermo Fischer Scientific*, *Sigma Aldrich* / *Merck*) in bio-grade quality and used without further purification. Tris-buffered saline (50 mM tris, 300 mM NaCl; TBS) and phosphate-buffered saline (137 mM NaCl, 2.7 mM KCl, 10 mM Na_2HPO_4, 1.8 mM KH_2PO_4; PBS) were freshly prepared from the respective compounds dissolved in ultra-pure water (dispensed from OmniaPure xs^{basic}, *Stakpure*), and pH was adjusted using a SevenCompact pH-meter (*Mettler Toledo*). Sterilization of buffers and stock solutions was achieved by vacuum filtration or syringe filtration through 0.2 µM polyvinylidene fluoride (PVDF) membranes. Concentration of protein solutions and foldamer solutions were determined by measuring absorbance at the respective absorption maxima (280 nm for proteins, 375 nm for foldamers) on a NanoDrop™ OneC photo spectrometer (*ThermoFischer Scientific*) and calculation via extinction coefficients. OD600 of bacterial cultures were monitored on the same device using 1 cm disposable cuvettes. Centrifugal concentrator units Pierce™ (*ThermoFischer Scientific*) with suitable molecular weight cut-offs were used to concentrate protein solutions. For buffer exchange Slide-A-Lyzer™ (*ThermoFischer Scientific*) dialysis cassettes were used.

All work with bacteria was performed under antiseptic conditions next to a Bunsen burner flame. Pipette tips and liquid containers were sterilized by autoclaving at 121°C. Transformation medium SOC (Super optimal broth with catabolite repression) was purchased pre-prepared (*Sigma Adlrich*), LB medium for bacterial growth cultures was prepared from solid LB broth (*CarlRoth*) and ultra-pure water and sterilized by autoclaving at 121°C. Ampicillin and Kanamycin were used as 1000 x stock solutions in respective dilution. Competent *E. coli* BL21(DE3) cells were purchased as cryo-stocks (*New England BioLabs*, *ThermoFischer Scientific*), stored at −80°C and handled on ice during usage.

Plasmids were purchased as lyophilized solids (*Genescript*) and dissolved in autoclaved water to a final concentration of 200 ng/µL. Bacterial colony selection on LB-agarose culture plates, containing 50 µg/mL of antibiotic were conducted overnight at 37°C. Cryo-stocks of selected colonies were produced by small culture growth overnight and liquid nitrogen flash freezing 600 µL of the growth media mixed 1:1 with 60% vol/vol sterile glycerol solution. IPTG for expression induction was used as 1 M sterile filtered stock solution in respective dilution. Pelleting of expression cultures was achieved by centrifugation in an Avanti JXN-26 (*Beckman Coulter*) centrifuge. Cell lysis was performed on an UP200St (*Hielscher*) ultrasonic homogenizer.

HisPur™ Ni-NTA resin (*ThermoFischer Scientific*) was used for affinity chromatography of His-tagged proteins.

SDS-PAGE gels were casted using commercially available SureCast™ (*ThermoFischer Scientific*) stacking and resolving buffers, aqueous acrylamide solution (40 vol%), APS and TMED, according to the manufacture's composition guide for the desired volume-percentage of acrylamide. Protein samples were mixed with 4 × sample loading buffer (0.2 M Tris-HCl, 8% vol/vol SDS, 6 mM bromphenol blue, 4.3 M glycerol) and heated to 95°C for 10 min prior to loading. Protein marker color prestained protein standard broad range 10 – 250 kDa (*New England BioLabs*) was loaded in the marker lane (3 µL). Electrophoresis was performed with 1 x SDS running buffer in a Mini-PROTEAN® Tetra Cell (*BioRad*) at 125 W/0.03 A, delivered by a PowerPac HC (*BioRad*) power supply. Separated proteins were stained with Brilliant Blue R Concentrate (*Sigma-Aldrich*) for 15 min under shaking and SDS-PAGE gels were subsequently destained in a H_2O/AcOH/MeOH (6:3:1, vol/vol/vol) solution.

Protein purification by FPLC-SEC was performed on a modular Azura system (*Knauer*) with a MWD 2.1L UV-detector, equipped with HiLoad 16/600 Superdex 75 and 200 pg SEC gel filtration columns (*Cytiva*) or on an Äkta Go system (*Cytiva*) with a UV on 9L Cpl, equipped with a HiLoad 26/600 Superdex 200 pg SEC gel filtration column (*Cytiva*). Chromatographic monitoring was performed at λ = 280 nm. Data was processed in OriginPro V.2019b (*OriginLab*).

1.2.3 Expression and purification of proteins

Standard recombinant protein expressions

All variants of C10 were expressed using either the pMAL-c5E or pET-28a(+) vector systems, encoding for the target sequence N-terminally fused to a HRV 3C cleavable MBP-10×His or 6×His tag respectively. Plasmids were transformed into competent *E. coli* BL21(DE3) cells by mixing 1 µL of plasmid stock solution with one tube of bacteria stock (50 µL), incubation on ice for 10 min and subsequent heat-shock at 42°C for 30 s. The transformed cells were cooled on ice for 5 minutes, mixed with 600 mL S.O.C. medium and incubated at 37°C for 1 h under shaking (600 rpm). The mixture was spread on LB-Amp (for pMAL-c5E) or LB-Kan (for pET-28a(+)) agarose plates and incubated over-night at 37°C. Subsequently, starter cultures were grown from single colonies in 10 – 50 mL LB medium containing the respective antibiotic (Amp: 100 µg/mL; Kan: 50 µg/mL) at 37°C overnight. Starter cultures showing visible growth were used for inoculation (1:100 vol%) of 250 mL – 4 L LB-Amp/Kan medium and cells were grown at 37°C under shaking (200 rpm) until an OD600 of 0.5 – 0.8 was reached. For pET-28a(+) constructs, expression was induced by adding IPTG (1 mM final concentration) and continued for 3 h at 37°C. For pMAL-c5E constructs the temperature was lowered to 22°C for 1.5 h and protein expression was induced by adding IPTG (1 mM final concentration) and continued for 18 h. After the expression cells were harvested by centrifugation at 8000 × g for 15 – 20 min at 4 °C. The supernatant was discarded, and cell pellets were either frozen at −20 °C or used directly in the next step.

Expression of [^{13}C,^{15}N]-labeled C10

The pMAL-c5E vector system, encoding for the target sequence N-terminally fused to a HRV 3C cleavable MBP-10×His tag was used for the expression of the [^{13}C,^{15}N]-isotopic labeled C10. The starter culture was grown from glycerol stocks of transformed cells in 25 mL LB-Amp medium at 37°C overnight. The overnight culture was then harvested by centrifugation at 4000 × g for 20 min, and the resulting cell pellet was resuspended in 1 L of M9 minimal medium supplemented with 1 g D-glucose-$^{13}C_6$ and 2 g $^{15}NH_4Cl$, 10 ml of 100x trace element solutions, 1 ml of 1 M $MgSO_4$, 0.3 ml 1 M $CaCl_2$, 1 ml biotin (1 mg/ml), 1 ml thiamin (1 mg/ml) and 100 µg/ml ampicillin. The culture was grown at 37°C under shaking (200 rpm) until an OD600 of 0.6 was reached. The temperature was reduced to 22°C prior to induction. Protein expression was induced by the addition of IPTG (0.5 mM final concentration). Cells were harvested after 18 h of expression by centrifugation at 8000 × g for 15 – 20 min at 4°C. The supernatant was discarded, and cell pellets were frozen at −20°C.

Small-scale protein purification

Harvested or frozen cell pellets were resuspended in PBS lysis buffer (PBS, 30 mM imidazole, 1×BugBuster, pH = 7.4) and incubated for 10 – 20 min. The lysate was cleared by centrifugation at 15000 × g and loaded onto 100 µL His SpinTrap™ columns. The resin was washed with 5 CVs wash buffer 1 (PBS, 30 mM imidazole, pH = 7.4), 2 CVs wash buffer 2 (PBS, 50 mM imidazole,

pH = 7.4), and the target protein was eluted with elution buffer (PBS, 300 mM imidazole, pH = 7.4). The final elution was concentrated, buffer-exchanged into PBS and either used directly in subsequent experiments or aliquoted and stored at −80°C.

Large-scale protein purification

Harvested or frozen cell pellets were resuspended in lysis buffer (TBS, 30 mM imidazole, pH = 7.4). Cells were sonicated in 4-5 rounds of 3 min each on ice (Frequency = 90%; Amplitude = 100%, Power = max) with 10 minutes breaks between rounds to prevent sample overheating. The resulting suspension was centrifuged at 45000 × g for 45 min at 4°C and the supernatant was incubated with Ni-NTA resin (5 mL suspension per 1 L of expression) at 4°C for 30 min to 1 h. The incubated resin was loaded onto a gravity-flow column, washed with 10 CVs of lysis buffer, 3 CVs of wash buffer (TBS, 50 mM imidazole, pH = 7.4) and the target fusion protein was lastly eluted with elution buffer (TBS, 300 mM imidazole, pH = 7.4). The UV absorbance at 280 nm was monitored using a Nanodrop photo spectrometer to track protein elution and assist with sample collection during the washing and elution steps. The elution fractions were combined, HRV 3C protease (1 u/100 µg) was added and the mixture was dialyzed against lysis buffer (TBS, 30 mM imidazole, pH = 7.4) overnight. The cleavage mixture was incubated with Ni-NTA resin for 30 min at 4°C and subsequently loaded on a gravity-flow column and the flow-through, containing the cleaved target protein was collected and concentrated for SEC purification in TBS.

Fractions containing the target protein were pooled, concentrated and either used directly in subsequent experiments or aliquoted and stored at −80°C.

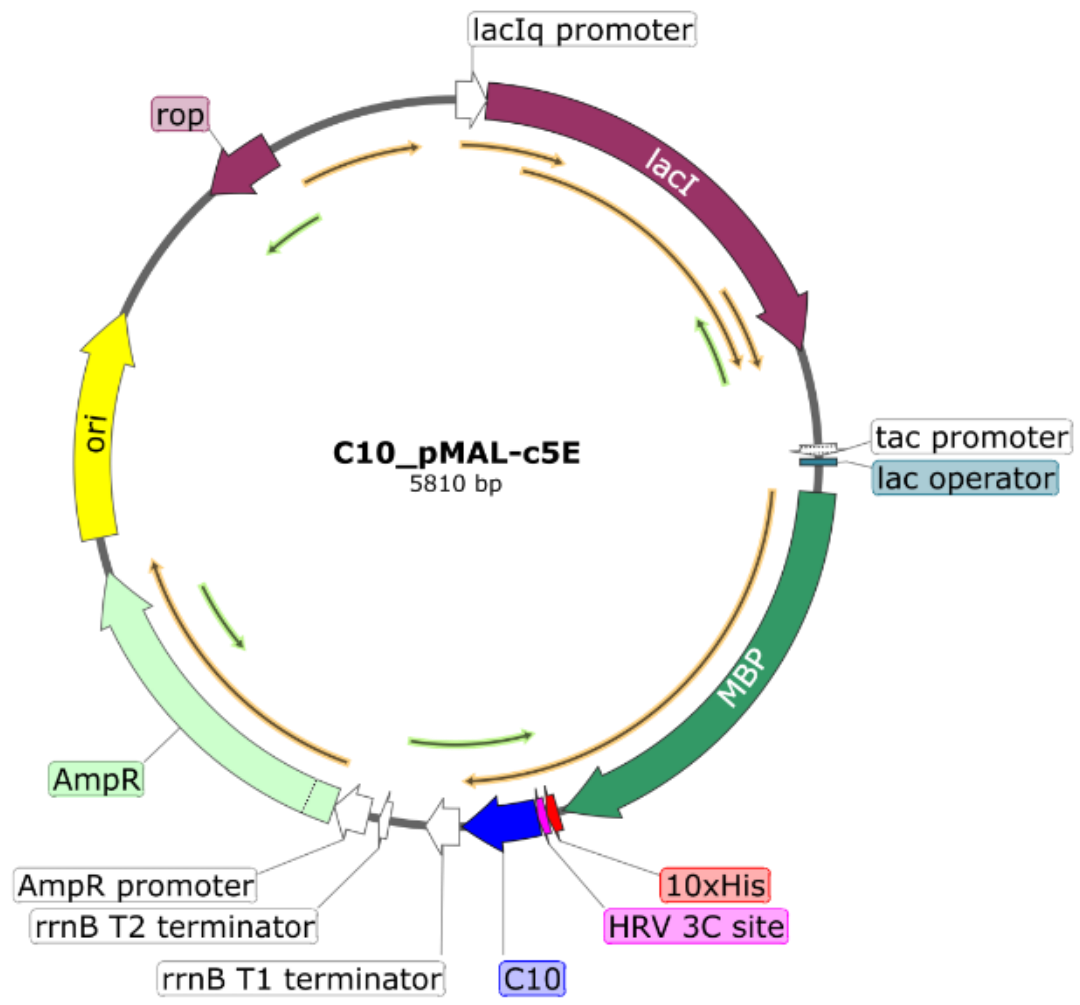

MKIEEGKLVIWINGDKGYNGLAEVGKKFEKDTGIKVTVEHPDKLEEKFPQVAATGDGPDI
IFWAHDRFGGYAQSGLLAEITPDKAFQDKLYPFTWDAVRYNGKLIAYPIAVEALSLIYNK
DLLPNPPKTWEEIPALDKELKAKGKSALMFNLQEPYFTWPLIAADGGYAFKYENGKYDIK
DVGVDNAGAKAGLTFLVDLIKNKHMNADTDYSIAEAAFNKGETAMTINGPWAWSNIDTSK
VNYGVTVLPTFKGQPSKPFVGVLSAGINAASPNKELAKEFLENYLLTDEGLEAVNKDKPL
GAVALKSYEEELVKDPRIAATMENAQKGEIMPNIPQMSAFWYAVRTAVINAASGRQTVDE
ALKDAQTNSSSHHHHHHHHHHLEVLFQ↓GPVKVKFKYKGEEKEVDTSKITHVFRHGKLVV
FYYDDNGKTGHGLVPEKDAPKELLDMLARAEREK*

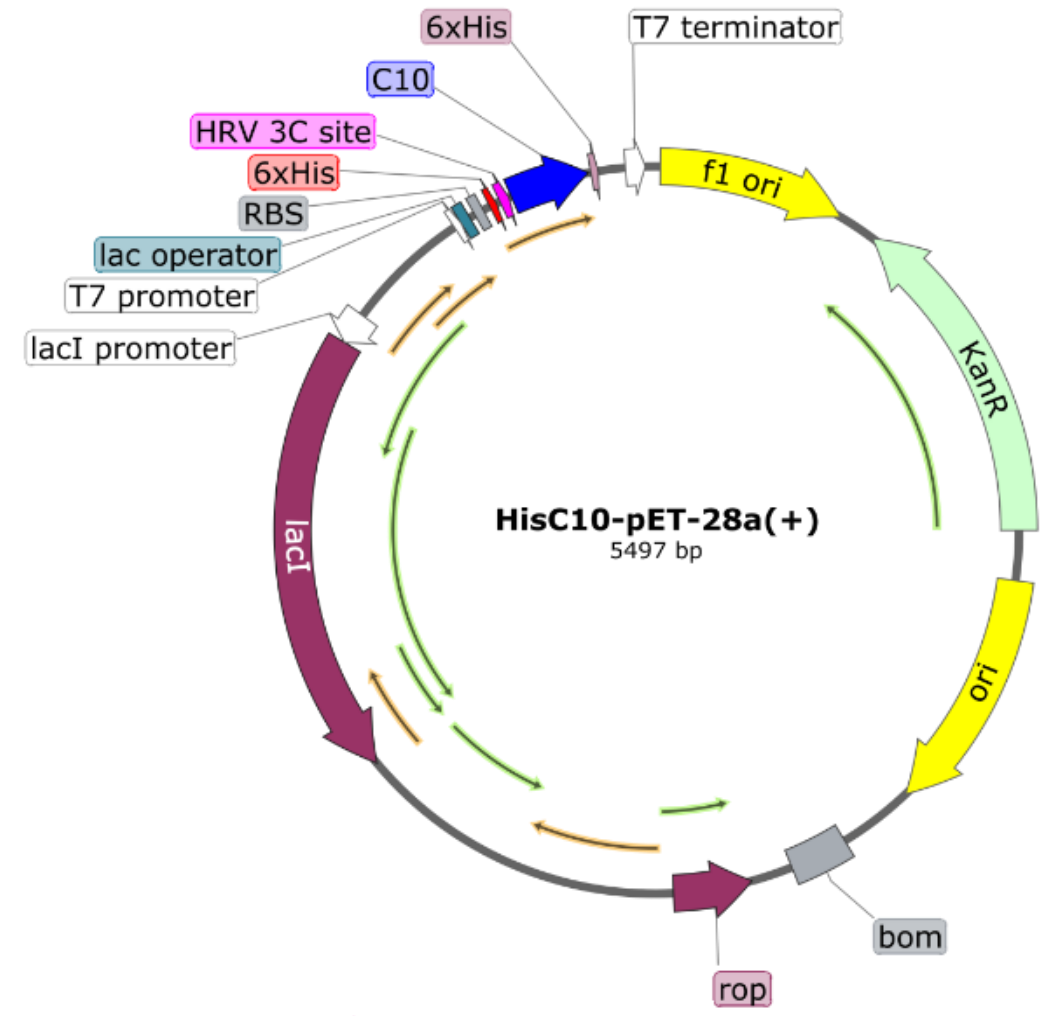

MGSSHHHHHHSSGLEVLFQ↓GPHMVKVKFKYKGEEKEVDTTKITHVARGG
KYVHFTYDDNGKTDTGRVPKKAAPKELLDMLARAEREK*

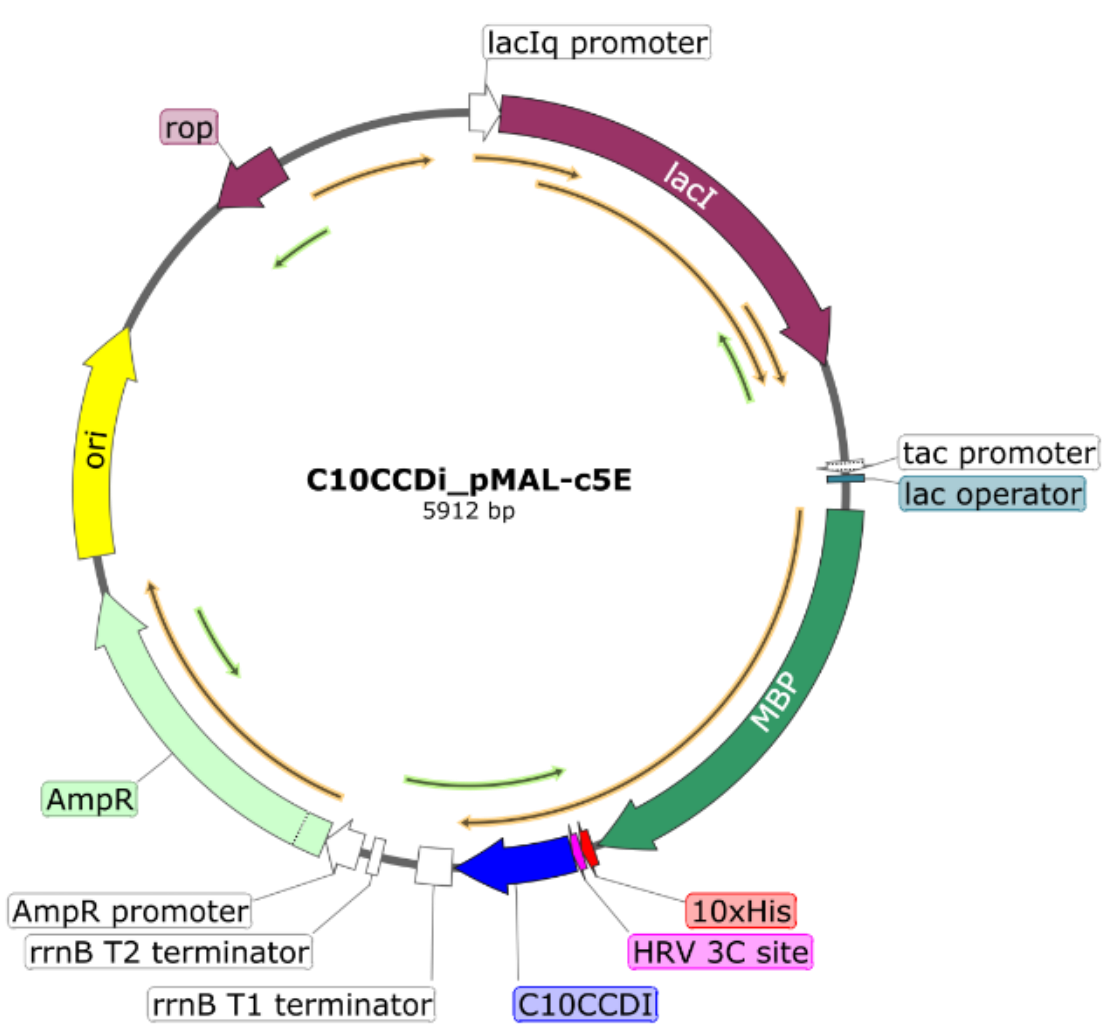

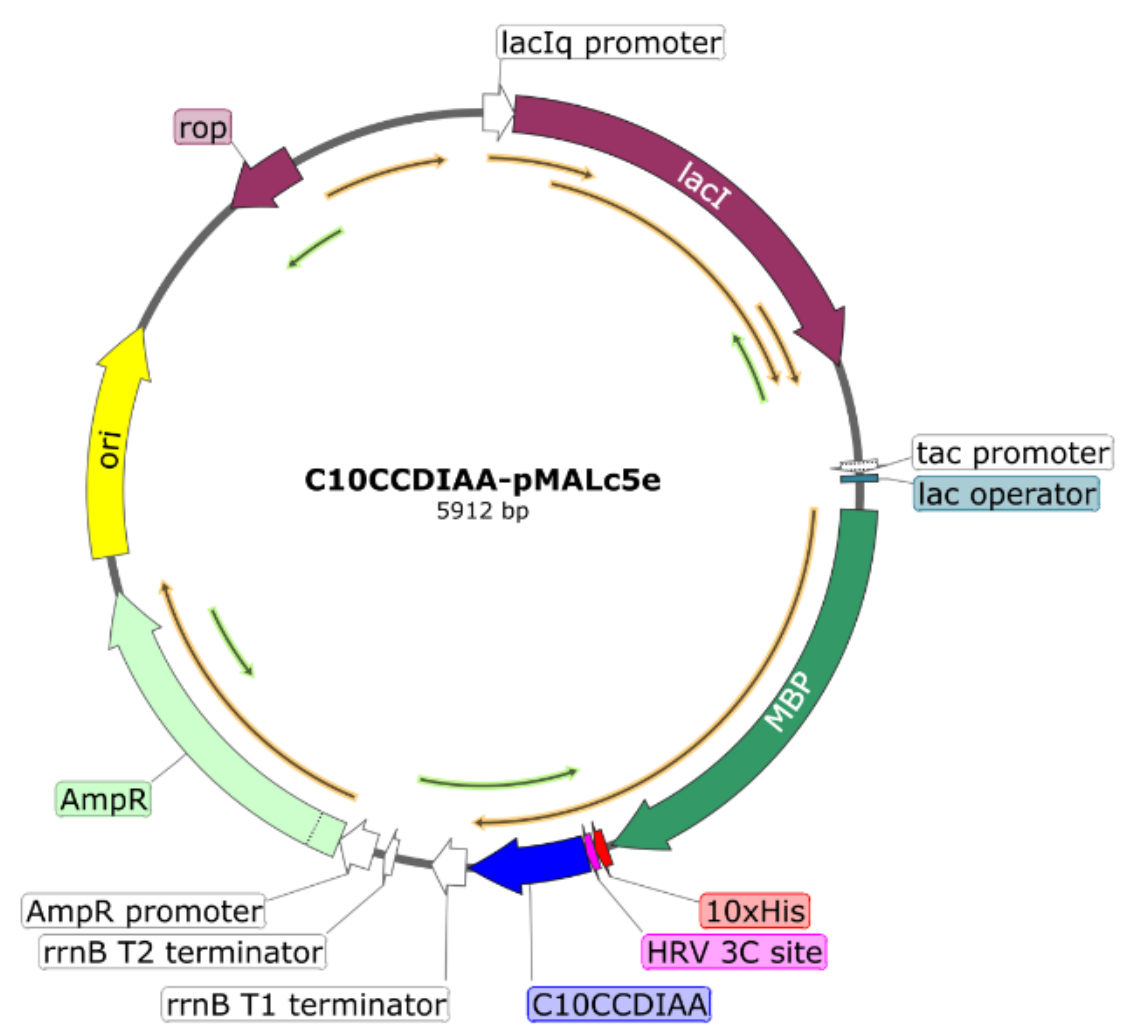

MKIEEGKLVIWINGDKGYNGLAEVGKKFEKDTGIKVTVEHPDKLEEKFPQVAATGDGPD
IIFWAHDRFGGYAQSGLLAEITPDKAFQDKLYPFTWDAVRYNGKLIAYPIAVEALSLIY
NKDLLPNPPKTWEEIPALDKELKAKGKSALMFNLQEPYFTWPLIAADGGYAFKYENGKY
DIKDVGVDNAGAKAGLTFLVDLIKNKHMNADTDYSIAEAAFNKGETAMTINGPWAWSNI
DTSKVNYGVTVLPTFKGQPSKPFVGVLSAGINAASPNKELAKEFLENYLLTDEGLEAVN
KDKPLGAVALKSYEEELVKDPRIAATMENAQKGEIMPNIPQMSAFWYAVRTAVINAASG
RQTVDEALKDAQTNSSSHHHHHHHHHHLEVLFQ↓GPVKVKFKYKGEEKEVDTSKITHVF
RHGKLVVFYYDDNGKTGHGLVPEKDAPKELLDMLARAEREKGGIAQIIAAQEEMLRKER
ELEEARKKLAQIRQQQ*

MKIEEGKLVIWINGDKGYNGLAEVGKKFEKDTGIKVTVEHPDKLEEKFPQVAATGDGPD
IIFWAHDRFGGYAQSGLLAEITPDKAFQDKLYPFTWDAVRYNGKLIAYPIAVEALSLIY
NKDLLPNPPKTWEEIPALDKELKAKGKSALMFNLQEPYFTWPLIAADGGYAFKYENGKY
DIKDVGVDNAGAKAGLTFLVDLIKNKHMNADTDYSIAEAAFNKGETAMTINGPWAWSNI
DTSKVNYGVTVLPTFKGQPSKPFVGVLSAGINAASPNKELAKEFLENYLLTDEGLEAVN
KDKPLGAVALKSYEEELVKDPRIAATMENAQKGEIMPNIPQMSAFWYAVRTAVINAASG
RQTVDEALKDAQTNSSSHHHHHHHHHHLEVLFQ↓GPVKVKFKYKGEEKEVDTSKITHVF
RHGKLVVFYYDDNGKTGHGLVPEKDAPKELLDMLARAEREKAAIAQIIAAQEEMLRKER
ELEEARKKLAQIRQQQ*

Expression vectors used in this work. C10 and C10 coiled-coil fusion protein sequences are marked blue, poly-His tags red and the MBP expression tag green. The recognition site for HRV-3C protease is highlighted in pink with a downwards arrow indicating the cleavage position. The stop codon is indicated with an asterisk.

1.3 Biophysical measurements and binding analyses

1.3.1 Circular dichroism (CD) spectroscopy

CD data were recorded on a J-1500 spectrometer (*Jasco*) using 1 or 2 mm optical pathlength quartz cuvettes. Fixed temperature spectra were measured at 1 nm bandwidth with a scan speed of 100 nm/min, baseline corrected and averaged over five accumulations. Variable temperature spectra were recorded in 10°C intervals, heating/cooling with a 5°C/min ramp rate, conducted in duplicate at each temperature and averaged. Variable temperature single wavelength CD were recorded in 0.5°C intervals, heating/cooling in 0.5°C steps with 5°C/min ramp rate and a 2 second delay for data acquisition at each point. Processing and plotting of the data were performed in OriginPro V.2019b (*OriginLab*).

1.3.2 Analytical size exclusion chromatography

Analytical SEC was performed on a modular Azura system (*Knauer*) with a MWD 2.1L UV-detector, equipped with a Superdex Increase 3.2/300 column (*Cytiva*). Protein-foldamer samples were mixed in stoichiometric proportions with a final complex concentration of 30 µM in TBS (300 mM NaCl, 50 mM TRIS, pH = 7.4) and injected via a 10 µL capillary loop. Chromatographic runs were performed with a flowrate of 0.07 mL/min at room temperature and monitored at λ = 280 nm and 375 nm. Data was processed in OriginPro V.2019b (*OriginLab*).

1.3.3 Mass spectrometry

Sample preparation and ionisation: Samples were ionized from solutions of 200 mM ammonium acetate and transferred to the gas phase by nano-electrospray (nESI) ionization from borosilicate glass emitters manufactured in-house on a Sutter P-1000 micropipette puller (*Sutter Instrument*). Emitters were manufactured pre-opened, to emitter opening-diameters of approximately 1.2 µm [60,61]. Ionization was initiated by applying a capillary voltage of 0.8-1.2 kV *via* a platinum wire inserted in contact with the sample solution.

Mass Spectrometry and Tandem Mass Spectrometry: A Q Exactive Ultra-High-Mass-Range (UHMR) Hybrid Quadrupole-Orbitrap Mass Spectrometer (*Thermo Fisher*) was used for mass spectrometry experiments[62]. Specifically, for MS^2 experiments, target ions were *m/z*-isolated in a quadrupole filter, accelerated to a user-defined kinetic energy and injected into the higher-energy C-trap dissociation (HCD) cell filled with nitrogen gas, before their transfer to the Orbitrap mass analyser.

Ion Mobility Mass Spectrometry (IM-MS): Experiments were performed on a Synapt G2-S platform (*Waters Corporation*) under typical native conditions. $^{TW}CCS_{N2\rightarrow He}$ values (TW = Traveling Wave; N2→He = measured in nitrogen and calibrated with CCS_{He} values) were obtained through calibration with ions generated from nESI ionization of ubiquitin, β-lactoglobulin (monomer and dimer) and bovine serum albumin (BSA) from solutions of 200 mM ammonium acetate. Calibration was performed by fitting of charge-normalized calibrant mobilities to a power-law, according to the methods and reference CCS_{He} values of Bush and Thalassinos[63, 64].

1.3.4 Bio-layer interferometry (BLI)

BLI experiments were performed on an Octet R8 instrument (*Sartorius*) according to manufacturers' recommendations. In all cases, Streptavidin (SA) Octet biosensors were soaked for

15 min in PBS prior to the assays. Before loading, a baseline was recorded in PBS+0.05% Tween 20 (PBS-T) for 60 seconds.

For single concentration binding assays, the SA biosensors were loaded with the biotinylated oligomers (1.5 – 2.5 µg/mL in PBS-T) for 30 s, followed by a washing and base line step in PBS-T for 30 s. The association of C10 (250 nM in PBS-T) was recorded for 120 s. The dissociation step was recorded in the same wells used for the baseline for 120 s.

For kinetic assays, the SA biosensors were loaded with the biotinylated oligomers (1.5 – 2.5 µg/mL in PBS-T) for 30 s, followed by a washing and base line step in PBS-T for 30 s. The association was recorded against 2 x serial column dilutions of C10 (concentration ranges are indicated at the respective figures) in PBS-T. Dissociation was recorded in the same wells used for the baseline. A SA biosensor without an immobilized foldamer was used as a control, to ensure no unspecific binding of the protein towards the sensor tips. All experiments were conducted at 25°C in triplicate. Data were processed in Octet Analysis Studio V.13 and K_D values were calculated from group fitting the kinetic curves of association and dissociation to a 1:1 binding model.

The raw BLI data have been deposited under https://data.ub.uni-muenchen.de/701/1/C10_BLI-raw_Data.zip and is provided in form of their respective experiment folders of the Octet system for easy access in Octet Analysis Studio directly. The folders each also contain the respective result files in .csv format.

Directory names are associated with plotted data in the main text and supplementary information as follows:

1_P-12mer loaded against His6-C10 1 uM

2_ M-12mer against His6-C10 1 uM + loading 2ug

These two folders contain the data for the BLI sensorgrams shown in Fig. 1**e** of *P*-**1a** (blue curves) and *M*-**1a** (red curves) used as ligands on SA-biosensors, against Nanofitin C10 (analyte) in a serial dilution from 250 nM to 62.5 nM in PBS with 0.05% Ttween-20 (PBS-T).

1_series Q5432 C10 250nM single conc PBST

This folder contains the data for the BLI sensorgrams shown in Supplementary Fig. 10b BLI sensorgrams of single concentration binding assays of 2a, 5, 6 and 7 against 250 nM of C10.

Bio-P-Q12mer + Bio- Q5mer against C10 1uM

This folder contains the data for the BLI sensorgrams shown in Supplementary Fig 10c, d Kinetic BLI measurements of **1a c** and **2a d** against a serial dilution of C10 from 500 nM to 15.625 nM. Curve fits are displayed as black dashed lines.

1.4 NMR Structure Elucidation & MD Simulations

1.4.1 Experimental procedure

The NMR spectra were measured on a Bruker Avance Neo spectrometer at a field strength of 800 MHz equipped with a cryogenic triple gradient probe. Data were collected using Topspin version 4 and processed using NMRPipe[65]. Spectra were analyzed using Sparky version 3.190 within NMRFAM-SPARKY version 1.470[66].

1.4.2 Chemical shift assignment

For the unbound Nanofitin C10 protein, the backbone chemical shift assignment was performed at a temperature of 298 K on a 270 µM sample of [^{13}C,^{15}N]-C10 in PBS, with 5% (vol/vol) DMSO-d_6 and 10% D_2O (vol/vol). Assignment spectra included 2D ^{1}H,^{15}N-HSQC, 3D HNCO, 3D

HNCACO, 3D HNCA, 3D CBCACONH, and a 3D HACACONH. During the assignment process it was clear that many backbone ^{1}H-^{15}N amide crosspeaks were not visible which was likely due to partial dimerization of the protein at NMR concentrations (Supplementary Fig. 3).

The protein backbone chemical shift assignment for the **1c**-bound C10 was performed at 298 K on a 550 µM sample of [^{13}C,^{15}N]-C10 in complex with 550 µM natural-abundance **1c**, in PBS with 5% (vol/vol) DMSO-d_6 and 10% (vol/vol) D_2O. Assignments of the C10 protein backbone $^{1}H^{\alpha}$, $^{1}H^{N}$, $^{13}C^{\alpha}$, $^{13}C^{\beta}$, $^{13}C'$ and $^{15}N^{H}$ nuclei were based on 2D ^{1}H,^{15}N-HSQC, 2D ^{1}H,^{13}C-HSQC, 3D HNCO, 3D HN(CA)CO, 3D HNCA, 3D HNCACB, 3D CBCA(CO)NH, and 3D HA(CACO)NH spectra. The completeness of assignment for the backbone nuclei are: $^{1}H^{\alpha}$ 92%, $^{1}H^{N}$ 95 %, $^{13}C^{\alpha}$ 96%, $^{13}C^{\beta}$ 97 %, $^{13}C'$ 85%, and $^{15}N^{H}$ 95%. The sidechain aliphatic ^{1}H-^{13}C assignments for C10 relied on 2D ^{1}H,^{13}C-HSQC, 2D constant-time methyl-centered ^{1}H,^{13}C-HSQC, 3D H(CCO)NH, 3D (H)C(CO)NH, and 3D (H)CCH-TOCSY spectra. Within a protein sidechain, the methylene ^{1}H and the methyl ^{1}H-^{13}C nuclei were not stereospecifically assigned. Aromatic ^{1}H assignments were based on 2D ^{1}H,^{1}H-NOESY (150 ms mixing time), 2D ^{1}H,^{1}H-TOCSY (60 ms mixing time) and 2D ^{1}H,^{1}H-DQF-COSY spectra collected on a 100% D_2O sample of 250 µM natural abundance C10 in complex with 250 µM **1c**, in PBS with 5% (vol/vol) DMSO-d_6. For the **1c** foldamer bound to C10, the chemical shift assignment initially relied on the sample with 550 µM [^{13}C,^{15}N]-C10 bound to natural abundance **1c** sample in 10% D_2O using a double-filtered 2D ^{1}H,^{1}H-NOESY (120 ms mixing time) for suppression of protein ^{1}H signals connected to ^{13}C and ^{15}N nuclei. Spin systems were completed using the 2D ^{1}H,^{1}H-NOESY (150 ms mixing time), 2D ^{1}H,^{1}H-TOCSY (60 ms mixing time) and 2D ^{1}H,^{1}H-DQF-COSY spectra collected on the 100% D_2O sample. For each quinoline sidechain, the methylene ^{1}H and the methyl ^{1}H nuclei were not stereospecifically assigned. Selected protein assignment spectra for **1c**-bound [^{13}C,^{15}N]-C10 are included in Supplementary Fig. 4 A list of assigned **1c** ^{1}H nuclei is found in Supplementary Table 2.

1.4.3 Initial structure calculation of the **1c**•C10 complex

The large size of the foldamer as the ligand molecule in the complex required us to approach the NMR structure determination using an atypical protocol that we derived from standard protein structure determination. As described below, we used a modified ARIA/CNS[67] procedure to create the initial structures of the **1c**•C10 complex and to assist with the NOESY assignment, followed by restrained MD simulations in AMBER[68, 69] to generate the structural ensemble.

A starting peak list for protein intramolecular ^{1}H-^{1}H NOE crosspeaks was obtained from a 3D simultaneous ^{13}C/^{15}N-edited ^{1}H,^{1}H-NOESY (120 ms mixing time) on the 550 µM **1c** complex with [^{13}C,^{15}N]-C10 in 90% H_2O/10% D_2O that was used in the chemical shift assignment. Similarly, the foldamer intramolecular ^{1}H-^{1}H NOEs were obtained from a 2D double-filtered ^{1}H,^{1}H-NOESY spectrum (120 ms mixing time) on the same sample. The use of the double-filtered pulse program suppresses ^{1}H signals connected to ^{13}C and ^{15}N from the isotopically enriched C10 protein, allowing for a spectrum of **1c** in the protein-bound form. Unambiguous intermolecular protein-foldamer NOEs were identified using a 2D x2-filtered ^{1}H,^{1}H-NOESY (120 ms mixing time) and looking for additional crosspeaks that appeared when compared to the double-filtered NOESY. This half-filtered spectrum retains the ^{13}C/^{15}N-filter for one dimension to suppress the protein ^{1}H signals, but the second dimension now includes all ^{1}H in the sample with new peaks arising from NOEs from the foldamer to the protein (Supplementary Fig. 5). A further combination of protein, foldamer, and intermolecular NOEs were obtained from the 2D ^{1}H,^{1}H-NOESY (150 ms mixing time) with the 250 µM natural abundance **1c**•C10 complex in 100% D_2O.

Due to the size of the complex and the large number of ^1H chemical shifts, it was not possible to unambiguously assign all NOESY crosspeaks in advance, and therefore we decided to follow the iterative NOESY assignment process provided by ARIA/CNS. This program has been parameterized for biomolecules and some small ligands, so the first step was to obtain the relevant parameter and topology files for the **1c** foldamer. We used the online tool PRODRG[70] to help create these files, which also required an initial step to divide the foldamer into two halves so as to not exceed the atom number limit. The PRODRG program also provides unique nomenclature for every atom (preserved in the final PDB files, as previously shown for ^1H nuclei in Supplementary Table 2 and Supplementary Fig. 6). This nomenclature was particularly useful as the atom names did not overlap with ARIA/CNS atomic definitions that were already optimized for biomolecules. Using the CNS output option, we created *toppar* and *param* files for **1c**, which were appended within the ARIA program files *atomnames.xml*, *iupac.xml*, *topallhdg5.3.pro* and *parallhdg5.3.pro*. Additional modifications were made to these files to correct small errors in foldamer dihedral angles during PDB template generation in ARIA/CNS from the raw PRODRG output. Similar to the process of calculating a protein structure with NMR data in ARNA/CNS starting from a linear peptide conformation, our approach also starts with a linear foldamer conformation to be folded based on the NMR restraints.

Following the procedure used in NMR protein structure determination, we collected the four NOESY peak lists described above: protein NOEs (*13c15n_noesy*, 722 peaks), foldamer NOEs (*x2_noesy*, 416 peaks), intermolecular NOEs (*inter_noesy*, 36 peaks), and general NOEs (*d2o_noesy*, 258 peaks). These peak lists included a mix of fully assigned, partially assigned and unassigned NOESY crosspeaks. The peak lists and peak heights, along with the chemical shift assignments, were converted to the ARIA2.3/CNS1.2 format using the *conversion.py* script. Using the chemical shift assignments for the complex, we also obtained 56 pairs of protein backbone φ and ψ dihedral angles for C10 using TALOS-N[71]. During the iterative rounds within ARIA2.3/CNS1.2, the distance and dihedral restraints were active in each of eight iterations, to arrive at final annotated and calibrated distance restraints based on the NOESY peak lists (see NMR statistics in Table S2). The advantage of the ARIA/CNS approach was the established method for dealing with unassigned NOE crosspeaks through iterative steps, and the inclusion of ambiguous restraints where there is overlap between two or more contributing crosspeaks in the spectra. However, the nature of the foldamer component in this complex, in that the conformation and complex formation involve a high number of aromatic interactions and stacking, meant that a different forcefield was required to generate the final **1c**•C10 structures. Therefore, from the final ARIA/CNS iteration we retained the calibrated unambiguous distance restraints as well as the protein backbone dihedral angle restraints to be used in a final step using the program AMBER.

1.4.4 Restrained molecular dynamics on **1c**•C10

1c was parameterized for MD as follows. A file with the partial charges of 1c containing AMBER-compatible atom types was first generated with Antechamber[72, 73]. The charge values were then replaced by those obtained by two-stage Restrained Electrostatic Potential (RESP) charge calculations performed with Multiwfn 3.8[74-75], starting from the single-point energy of the crystal structure, calculated in Orca 6.0 using density functional theory with the B3LYP functional and 6-31G(d) basis set, as implemented in Orca 6.[76-87]. The resulting charge file was used to prepare a **1c** prepin file describing the topology and parameters of **1c** was generated using prepgen, and the corresponding force field parameters were obtained with parmchk2[88].

The **1c**•C10 complex was prepared for restrained MD in Amber24 using Leap[88]. The complex was described using the ff19SB force field complemented by the **1c** parameters derived above[89]. The complex was solvated in a truncated octahedral water box, with a 14 Å minimum distance from the solute to the box edge, using the OPC model with Li/Merz ion (12-6 set)[90, 91].

The number of sodium and chloride ions added to neutralize the system and set the ionic strength to 150 mM were determined using the SPLIT and SLTCAPS methods, yielding identical results[92, 93].

A robust 10-step minimization and relaxation protocol was used to resolve any close contacts and stabilize system density prior to production, as described by Roe and Brooks elsewhere[94].

All minimization steps were performed on double-precision CPU (to avoid numerical overflowing) using pmemd, while MD production runs were executed with pmemd.cuda on an NVIDIA H100 PCIe Tensor Core GPU, hosted on the DOREMI CALI v3 cluster at the *Mésocentre de Calcul Intensif Aquitain* (Université de Bordeaux, France)[95-97]. A minimized ensemble was produced by minimization of the complex after 20 independent 10-ns production runs under NPT conditions (1 atm, 300 K), with the following parameters: Langevin thermostat (collision frequency: 5 ps^{-1}), Monte Carlo barostat, 9 Å non-bonded cutoff, SHAKE constraints on bonds involving hydrogen, 20.0 $kcal.mol^{-1}.Å^{-2}$ distance restraint force constant, 100.0 $kcal.mol^{-1}.rad^{-2}$ torsion restraint force constant. Ensemble statistics are reported in Table S3, with final intermolecular distance restraints listed in Supplementary Table 4. All distance restraints are shown on a representative model in Supplementary Fig. 7.

Longer runs were conducted in triplicate for 1 µs, with different random seeds used across runs to avoid synchronization artifacts[98]. Trajectory and ensemble analysis, visualization and depiction was carried out using cpptraj 6.18.1, PLIP, 2.3.0, PyMOL 3.1.6 (Schrödinger, LLC), ChemDraw 25.0 (Revvity Signals Software, Inc.) and R 4.5.1[99-102]. Pairwise RMSD were computed for each production run on C10 backbone atoms and **1c** heavy atoms after alignment on C10. Atomic fluctuations were calculated for all C10 atoms, summarized as mass-weighted average fluctuation by residue (Supplementary Fig. 8). H-bond analysis was carried out on the concatenated 3-µs trajectory (3.2 Å, 135° cut-offs; Supplementary Fig. 9). Equivalent heavy atoms from donor/acceptor groups (e.g. OCV and OCX or ODF and ODE are pairs of equivalent oxygen atoms from carboxylate groups of **1c**) were grouped and H-bond formation events were concatenated within these groups. Formation frequencies and mean distances were recalculated accordingly.

1.4.5 Data deposition

The chemical shift assignments for the unbound and **1c**-bound C10 were deposited in the BMRB (https://bmrb.io/) under accession numbers 53300 and 53317, respectively. The ensemble of 20 structures calculated for the **1c**•C10 complex have been deposited in the PDB under accession 9T0O. It must be noted that residue numbering of the protein in the deposited files, supplementary figures and tables can differ from the conventions used in crystal structure files and annotations throughout this work to facilitate some aspects of the calculations starting the chain at residue 1 including the cleavage-site overhang.

1.5 Molecular modelling and other computational tools

Molecular modelling was carried out in Maestro MacroModel (*Schrödinger*) version 11.5[103]. Energy minimizations were run with the OPLS3 forcefield using the embedded PRCG method in water with a gradient convergence threshold of 0.05 $kJmol^{-1}$.

Molecular model visualization, surface area calculations and structure alignments/superpositions were carried out in PyMol (*Schrödinger*) version 3[104].

Pore bottleneck calculations were carried out with LifeSoaks, accessed via the proteins.plus web interface[48, 105].

1.6 Crystallography

1.6.1 Crystallization of C10 coiled-coil dimer, *P*-**2b**•C10CE, (*P*-**3**•C10)$_2$ and **4**•C10CE

Initial broad screening for crystallization conditions of all compounds was carried out at the Crystallization Facility of the Max-Planck-Institute for Biochemistry (Martinsried, Germany) in small scale (200 – 300 nL) sitting drop vapor diffusion experiments. Optimization of conditions was conducted in hanging-drop (0.5 – 2 µL) vapor diffusion set-ups at 4°C.

Crystals of the C10 coiled-coil dimer were obtained using 25% PEG 2000 and 50 mM HEPES pH 7.5 as the precipitant, with a protein stock concentration of 2 mM at 4°C.

For *P*-**2b**•C10-CCDi, a 2 mM complex solution was incubated for 72 h at room temperature prior to the crystallization set-up. Crystals were obtained from 20% PEG 400 and 0.1 M Sodium cacodylate pH 6.5 as the precipitant.

Crystals of (*P*-**3**•C10)$_2$ were obtained using 40% PEG 400, 200 mM Li_2SO_4 and 100 mM TRIS pH 8.5 as the precipitant.

Crystals of **4**•C10-CCDi were obtained using 30% PEG 550 MME, 30 mM $MnCl_2$ and 30 mM Na-cacodylate pH 5 as the precipitant.

Crystals of the C10 coiled-coil dimer were cryoprotected with 25% ethylene glycol in the mother solution prior to flash-freezing in liquid nitrogen. Crystals of *P*-**2b**•C10-CCDi, (*P*-**3**•C10)$_2$ and **4**•C10-CCDi were frozen directly from the crystallization drop.

1.6.2 X-ray data collection, structure determination and refinement

X-ray diffraction data were collected at the P13 and P14 beamlines of the European Molecular Biology Laboratory EMBL (Hamburg, Germany) equipped with a EIGER 16M detector[106].

Data were processed in CrysalisPro[107] or XDS[108]. The structures of the apo C10 coiled-coil dimer and (*P*-3•C10)$_2$ were solved by molecular replacement in PHASER[109] using the wildtype Sac7D structure (PDB: 1AZP)[110] as the search model. **2b**•C10-CCDi and **4**•C10-CCDi were solved in the same program, using the refined models of the other structures. Structure refinement was carried out iteratively in PHENIX refine[111], accompanied by assessments and real-space model building in COOT[112]. Unambiguous electron density was clearly visible for the foldamers after the initial few rounds of refinement. The foldamers were subsequently modelled into this density and the structures were further refined with geometry restrains CIF files generated in eLBOW[113] or Grade2[114]. Data collection and refinement statistics are provided in Supplementary Table 1.
Crystal structure data were submitted to the PDB and are accessible under the accession numbers: 9QDO, 9QT7, 9QOG & 9QNU.

2. Supplementary Figures

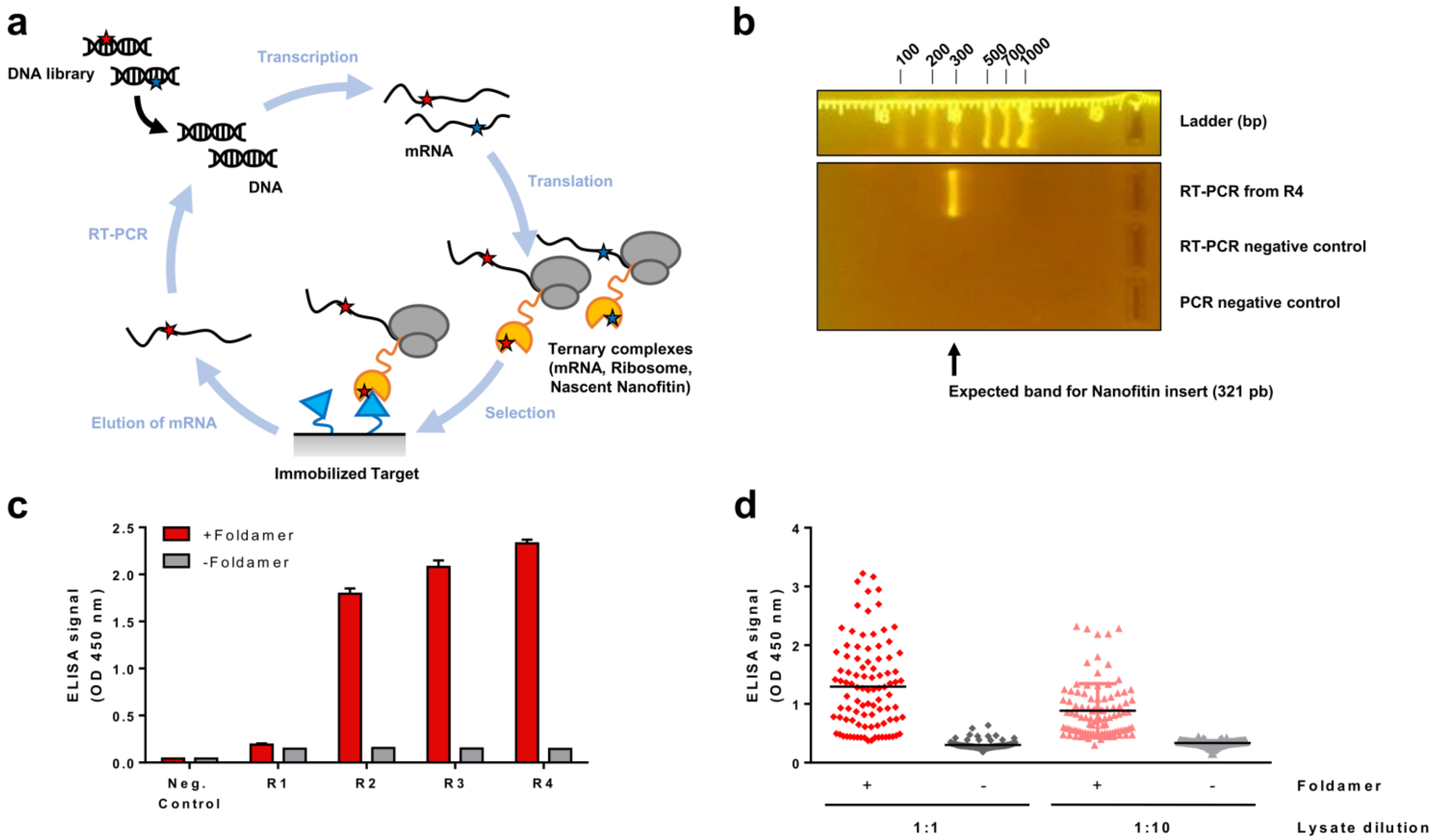

Supplementary Figure 1 | Ribosome display selection against 1a.

a Overview of the Nanofitin selection performed in vitro against AOF **1a**. From a DNA library derived from the Sac7d gene incorporating randomized positions within the protein paratope (stars), multiple selection rounds were performed against the immobilized foldamer in order to enrich Nanofitin binders with affinity and specificity for the target. **b** Amplification confirmation after 4 rounds of selection, assessed by electrophoresis on agarose gel. A single band at the expected size was observed after reverse transcription (RT) and subsequent PCR on the mRNA eluted from round 4 (R4). **c** Enrichment monitoring over selection rounds assessed by ELISA. The biotinylated foldamer was immobilized on coated streptavidin, then exposed to in vitro translation mix of RGS-Histagged Nanofitins obtained after rounds R1 to R4. The presence of Nanofitins bound to the immobilized target was revealed *via* an anti-RGSHis-HRP conjugate. **d** Isolated Nanofitins obtained after the fourth selection round were expressed in E. coli in 96-wells plate, then crude extracts were assessed by ELISA, undiluted or diluted to 1:10, in presence or absence of immobilized foldamer.

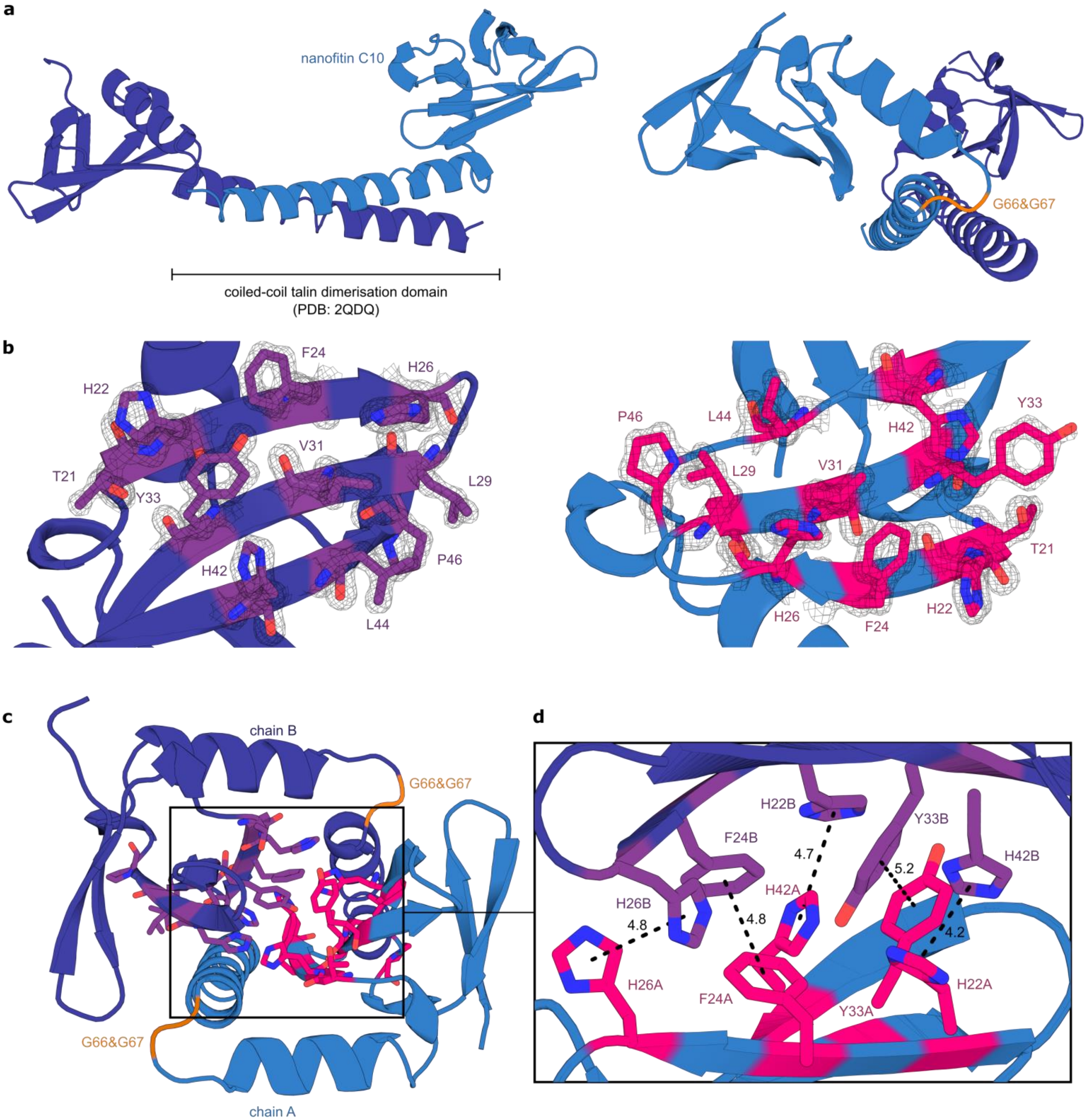

Supplementary Figure 2 part1

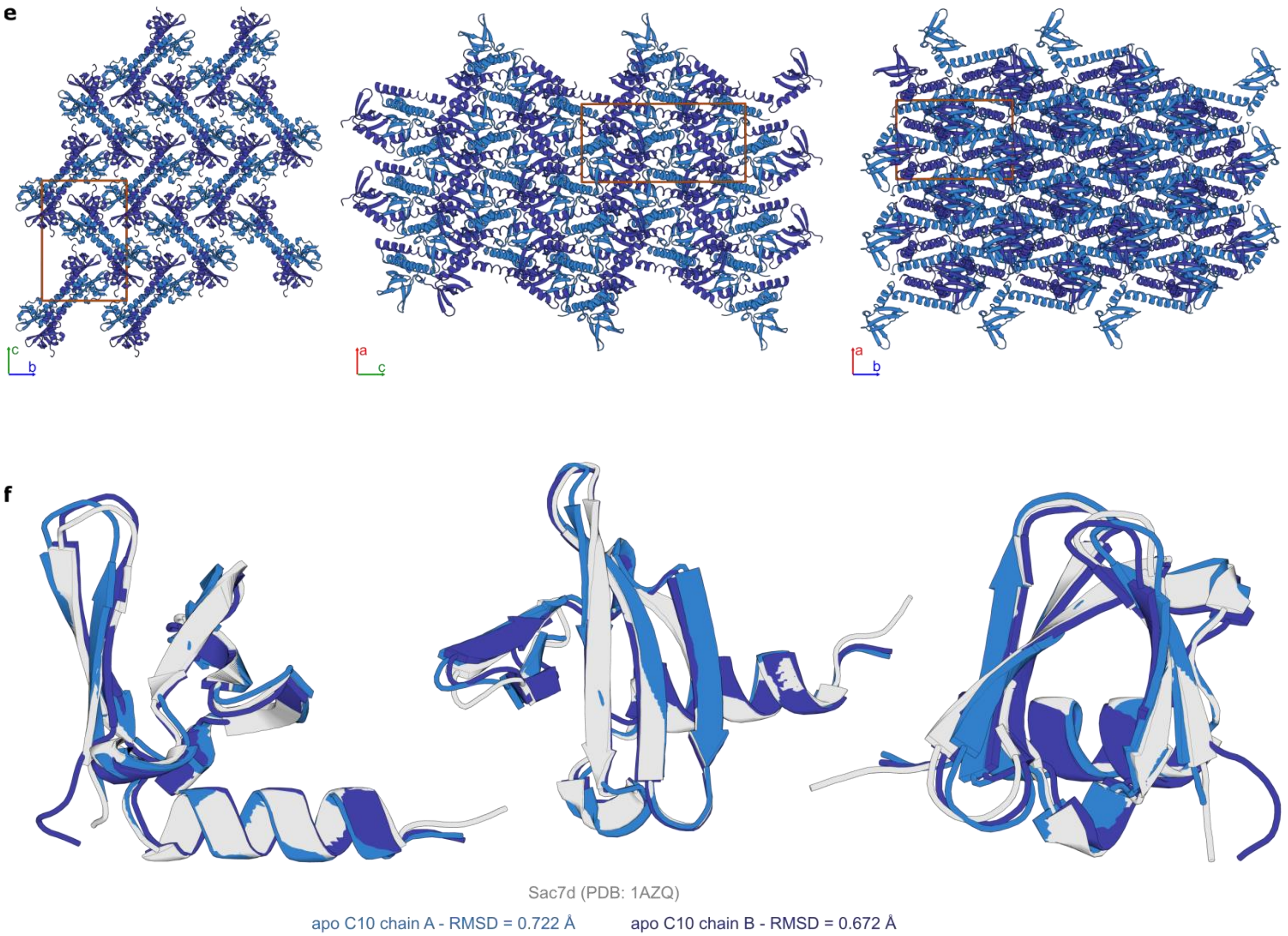

Supplementary Figure 2 part 2 | Crystal structure of the selected Nanofitin C10 fused to a coiled coil domain at 1.4 Å.

a ASU of the crystal structure, containing one coiled-coil mediated dimer of C10, shown from two perspectives. A similar approach to promote crystallization via multimerization was reported for the wild-type parent Sac7d protein using GCN4 derived domains (PDB: 1WVL)[115]. The disruption of the α-helical fusion of the Nanofitin to the coiled-coil domain induced by consecutive glycine residues (Gly66&67) is highlighted in gold. **b** Close-up of the three-stranded β-sheet region of C10, with residues differing from the wild-type protein Sac7D highlighted in purple and pink. Electron density map (2Fo-Fc) around relevant residues is shown at 2 σ. **c-d** Contacts between C10 chains A and B in the crystal structure, mediated by aromatic/hydrophobic residues give rise to an apparent face-to-face dimer in the solid state. Distances are given in Angstrom. **e** Crystal packing of the C10 coiled-coil apo structure, viewed orthoscopically along (100) -left, (010) -center and (001) -right. The unit cell is outlined in dark orange. **f** Alignment of the two C10 core structures to the parent wild type Sac7D (PDB: 1AZQ), from different perspectives, showing the fold conservation of the selected protein. Alignment and RMSD calculations were performed through the alignment plug-in in PyMol.

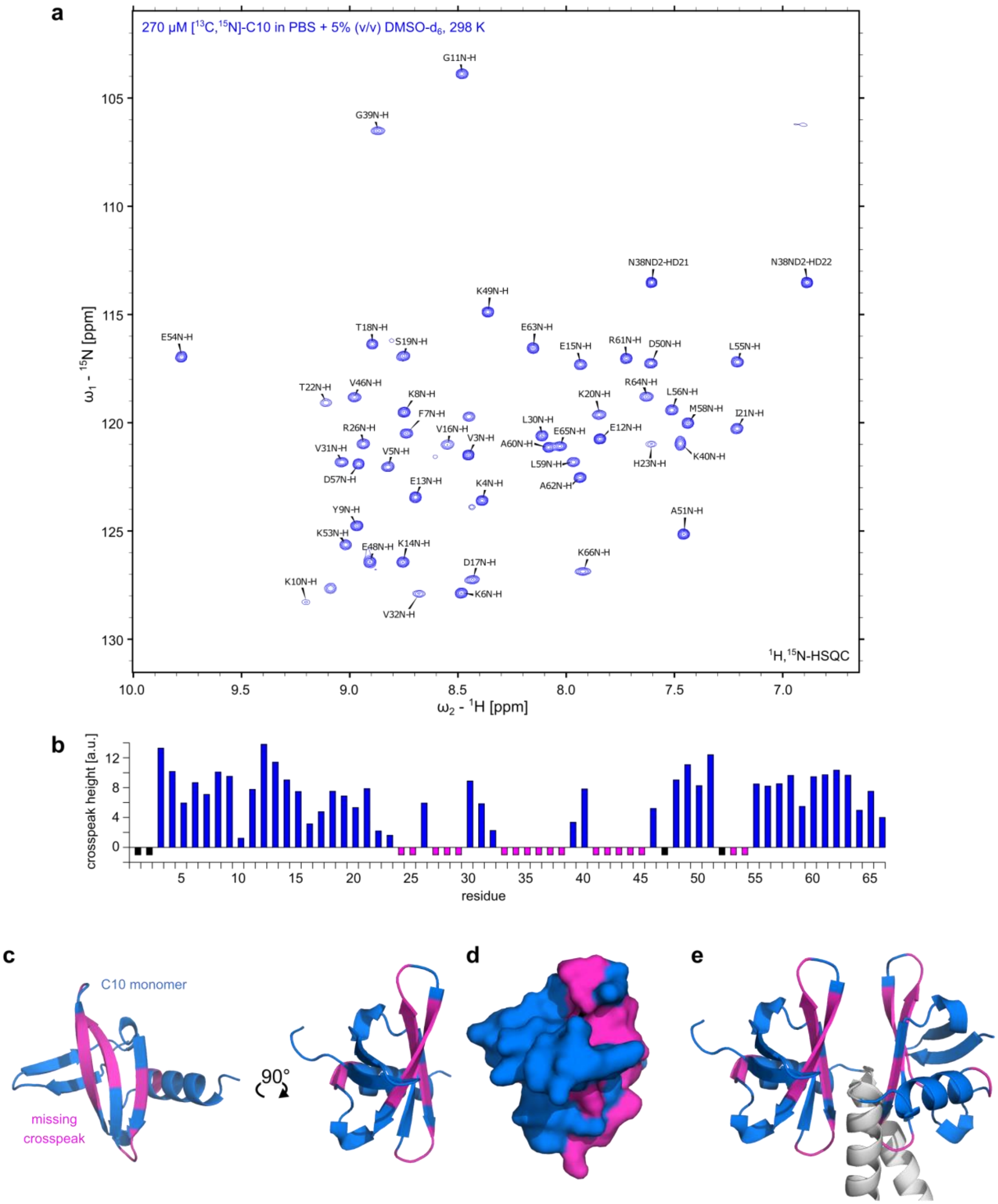

Supplementary Figure 3 | NMR spectroscopy of unbound Nanofitin C10.

a ^{1}H,^{15}N-HSQC spectrum of 270 µM [^{13}C,^{15}N]-C10 in PBS at 25 °C. Backbone amide crosspeaks are annotated by residue code and number, and the sidechain Asn amide crosspeaks are also annotated. Note that several backbone amide crosspeaks are broad and not visible. **b** Backbone amide crosspeak heights from the spectrum in A in arbitrary units. Residues lacking a backbone amide (N-terminus, proline) are shown as negative values in black, and residues with strong line-broadening are shown with negative values in magenta. Note that residue numbers are shifted by one unit in the NMR structure with respect to the crystal structures (see section 4.5). **c** Ribbon representation of the X-ray structure of C10 from this work highlighting in magenta the position of residues with strong backbone amide line-broadening. **d** The structure orientation at right in panel C is shown as a surface representation. **e** The residues with strong line-broadening correspond to the same surface region involved in protein-protein contacts in the crystal, suggesting that weak dimerization present for unbound C10 in the NMR sample involves the same interaction.

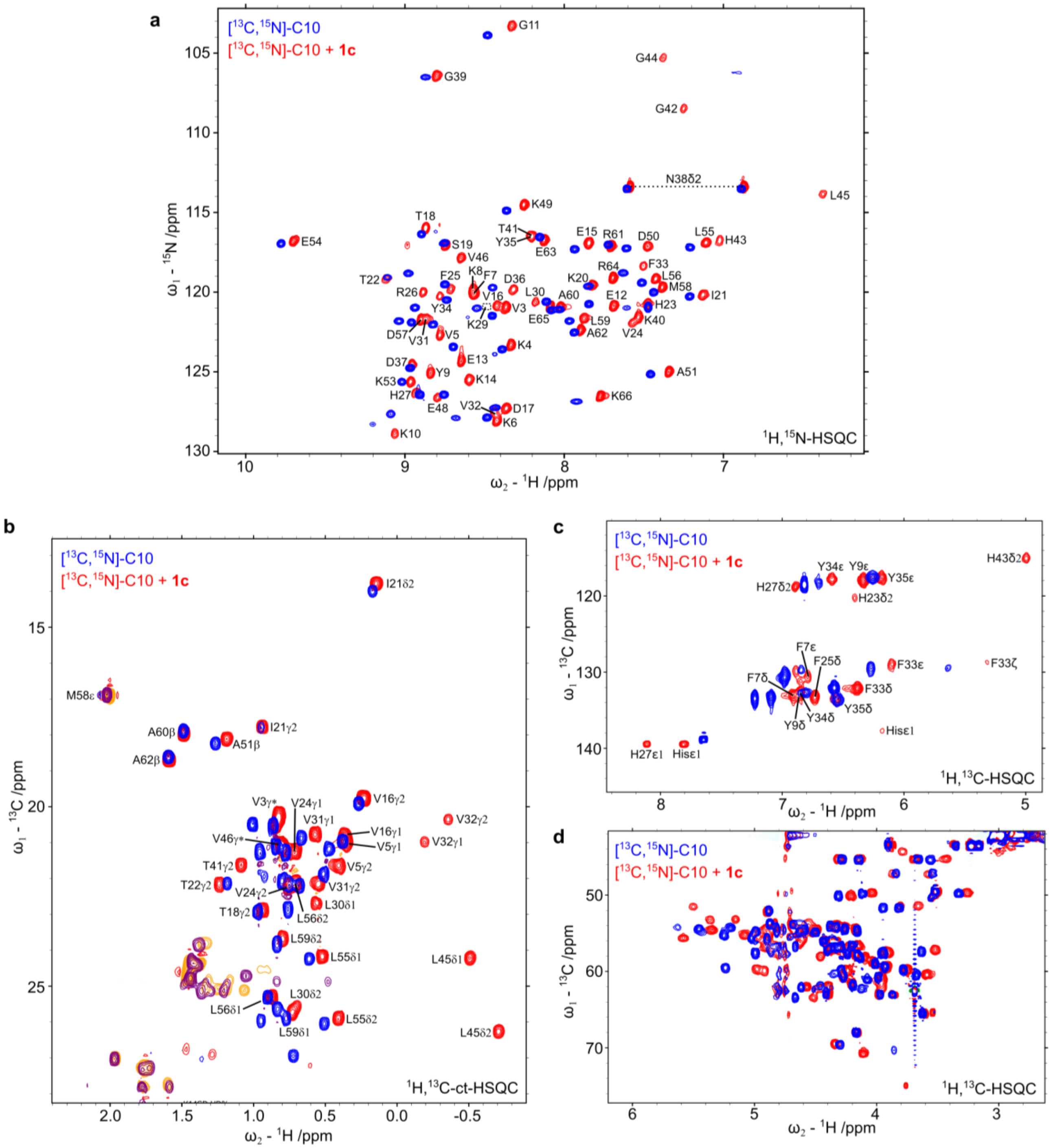

Supplementary Figure 4 | NMR spectroscopy of Nanofitin C10 in complex with dodecaamide foldamer 1c.

a-**e** Overlay of 800 MHz spectra measured at 298 K on unbound [^{13}C,^{15}N]-C10 (blue) on **1c**-bound [^{13}C,^{15}N]-C10 (red). The foldamer-bound protein sample contains 550 µM [^{13}C,^{15}N]-C10 bound to natural abundance **1c** in PBS with 10% (vol/vol) D_2O. **a** ^{1}H,^{15}N-HSQC spectrum of backbone amide crosspeaks with the **1c**-bound [^{13}C,^{15}N]-C10 crosspeaks annotated by residue code and number. The sidechain Asn amide crosspeaks are also annotated. An annotated spectrum for unbound [^{13}C,^{15}N]-C10 appears in Supplementary Fig. 3. **b** Methyl region from constant-time ^{1}H,^{13}C-HSQC spectra with only the **1c**-bound crosspeaks annotated. Note that for leucine and valine the annotation of the two methyl groups are not stereospecific, but are randomly assigned. **c** Aromatic region from ^{1}H,^{13}C-HSQC spectra with only the **1c**-bound crosspeaks annotated. **d** Alpha-carbon ^{1}Hα-^{13}Cα region from the same spectra shown in **c**.

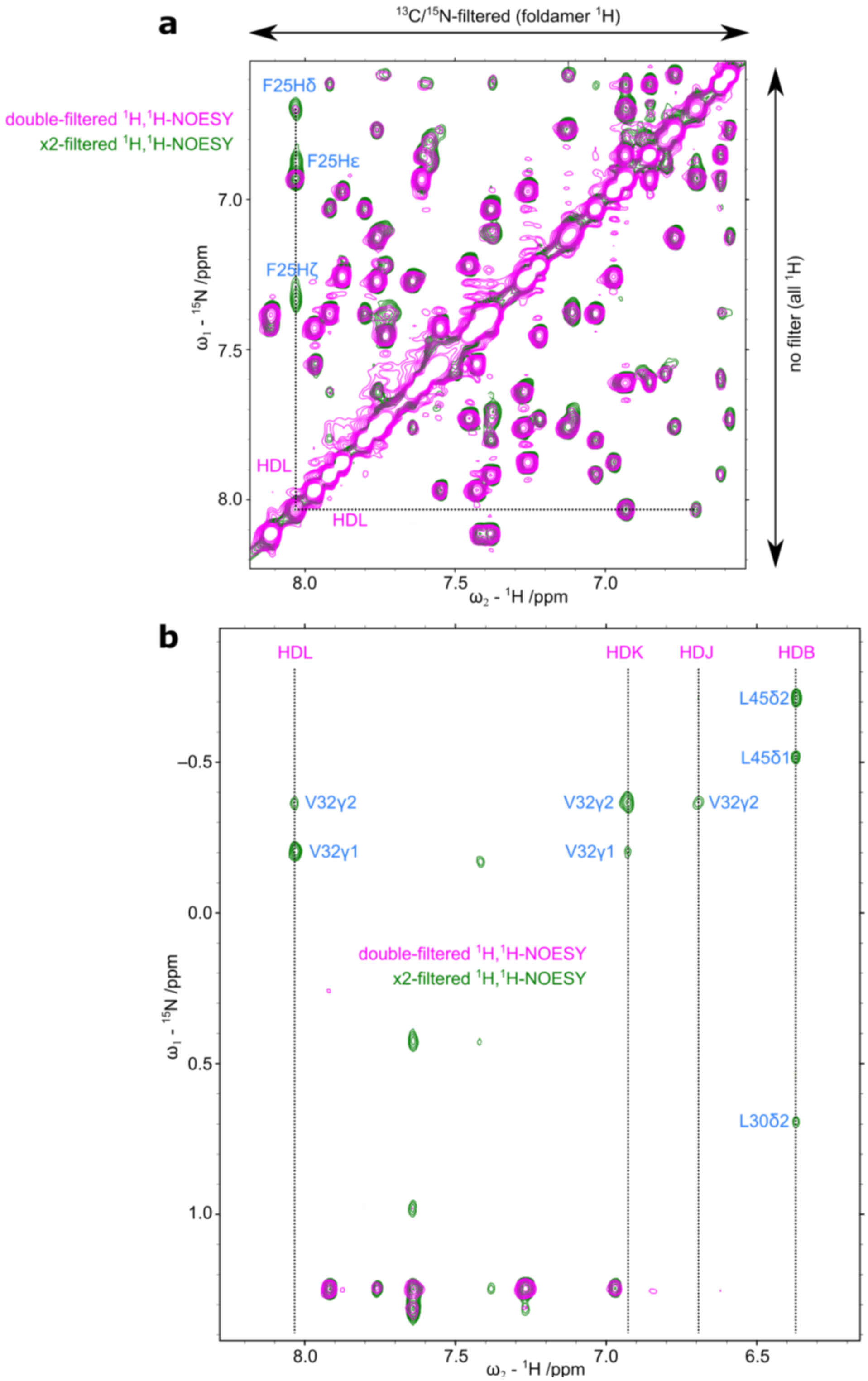

Supplementary Figure 5 | 2D filtered NOESY spectra on [^{13}C,^{15}N]-C10 bound to natural abundance 1c.

a Selected region from an overlay of double-filtered (xf) and single-filtered (x2) NOESY spectra, on a sample of 550 µM [^{13}C,^{15}N]-C10 bound to natural abundance **1c** in PBS with 10% (vol/vol) D_2O. NOE mixing time was 120 ms and spectra were measured at 298 K at a field strength of 800 MHz. In the double-filtered NOESY, both axes have been filtered to remove ^{1}H signals connected to ^{13}C or ^{15}N nuclei. In the single-filtered NOESY, the vertical (ω1) axis has no filter and corresponds to all ^{1}H nuclei. **b** A different region from the same spectra as **a**, illustrating several protein methyl group ^{1}H NOE crosspeaks to **1c** foldamer ^{1}H nuclei.

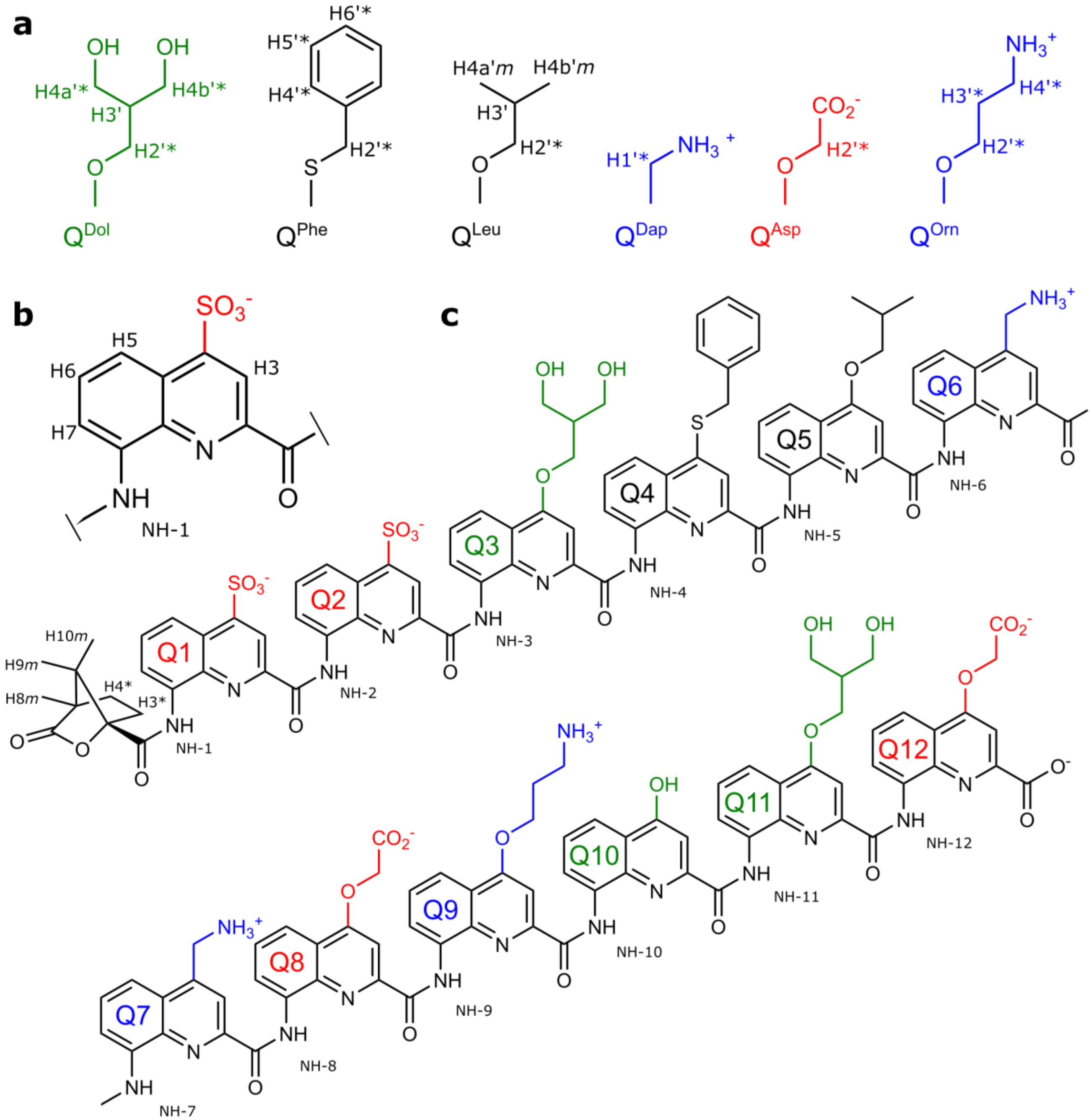

Supplementary Figure 6 | Nomenclature used for 1c NMR assignment and structure determination.

a Nomenclature used for the ‘sidechain’ hydrogens in foldamer **1c**. **b** Hydrogen numbering around the quinoline rings. **c** Overall identity of each quinoline monomer in **1c**, along with the unambiguous naming of the exchangeable amide hydrogens. Also shown is the hydrogen numbering used for the N-terminal camphanyl group.

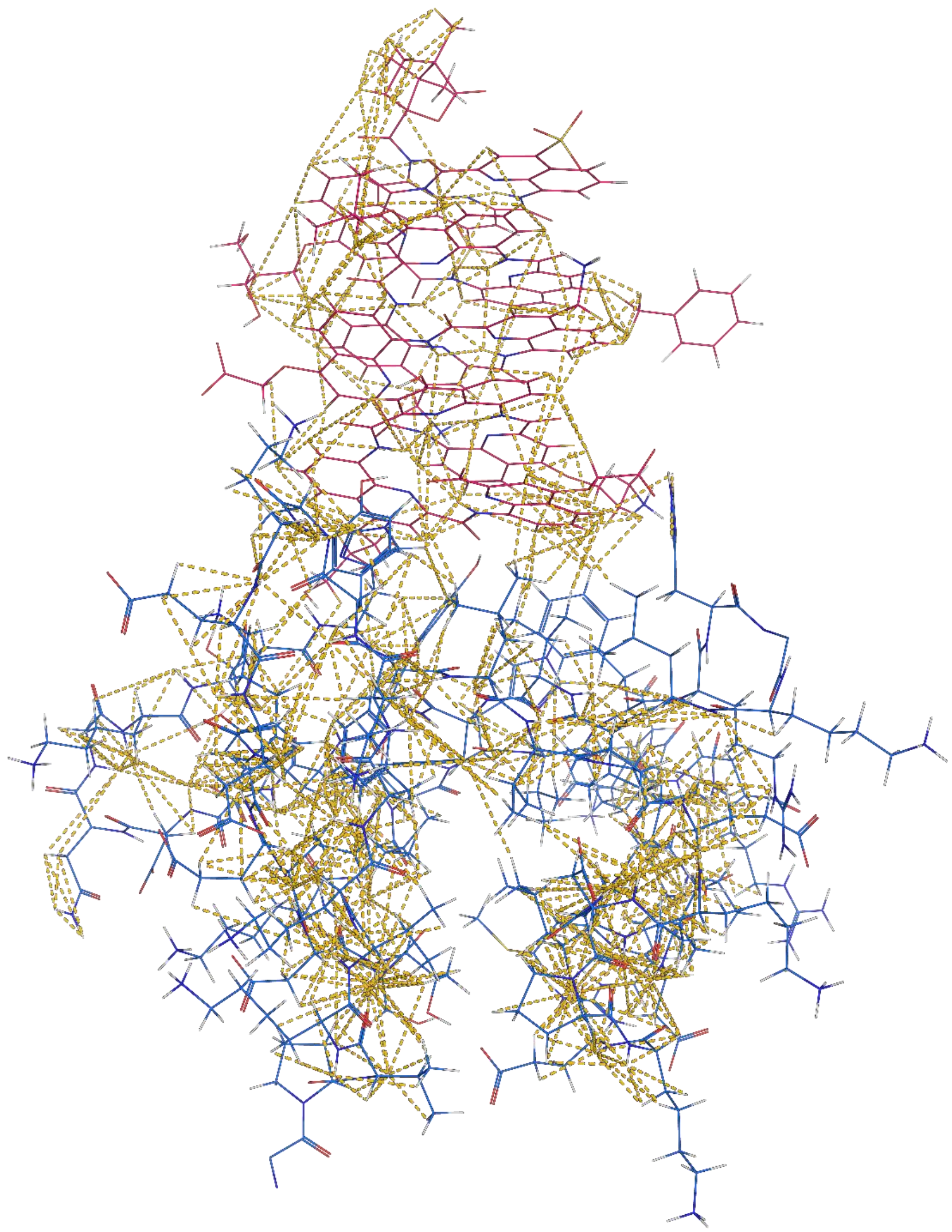

Supplementary Figure 7 | NMR distance restraints mapped onto representative model of 1c·C10.

The protein and foldamer are shown in line representation, with all 737 distance restraints indicated with yellow dotted lines. Note that for equivalent atoms (such as the three methyl hydrogens) a single distance is shown to the closest hydrogen.

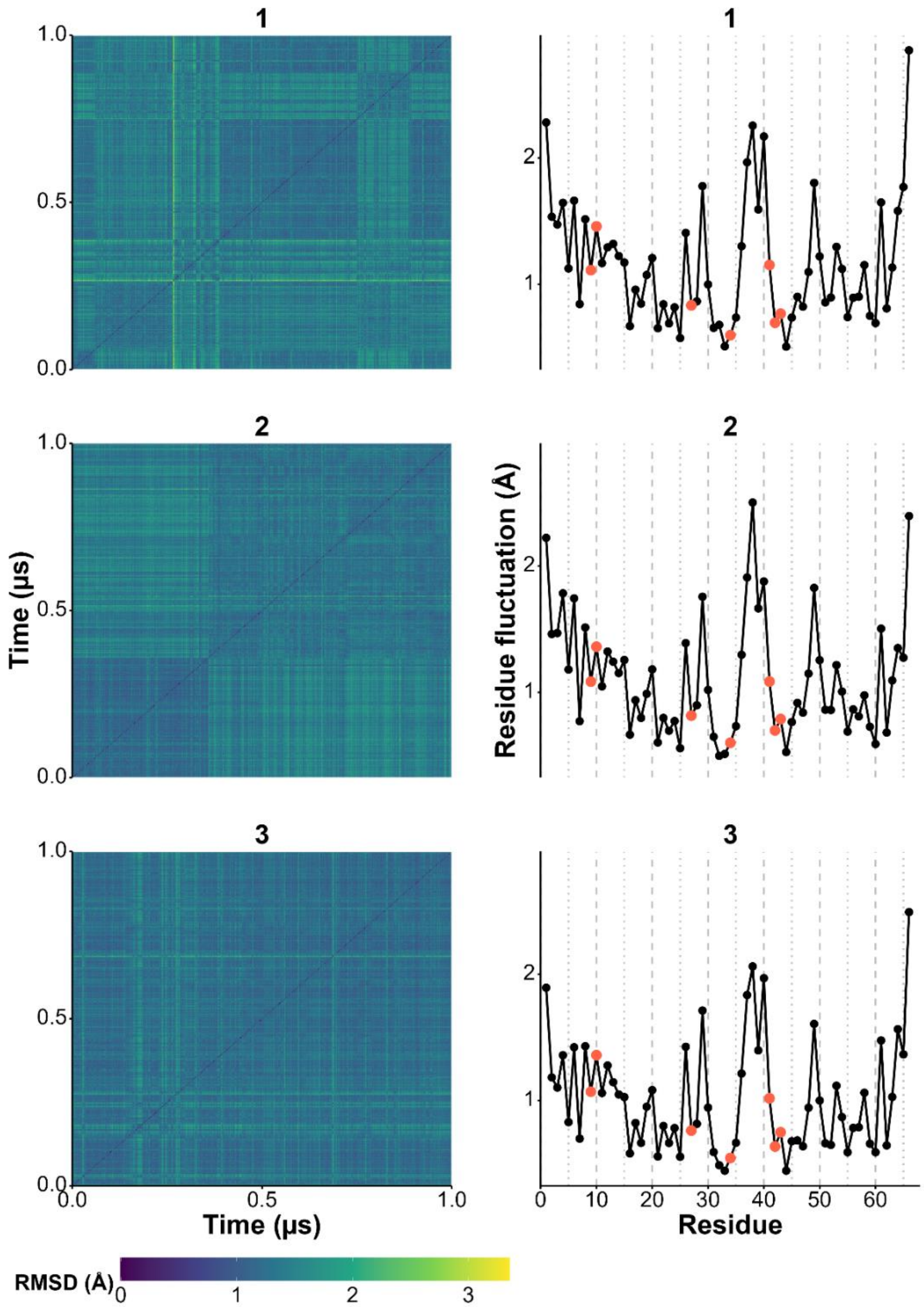

Supplementary Figure 8 | Atomic fluctuations during the microsecond production MD of the 1c•C10 complex.

The MD was run in triplicate (numbered 1 to 3). Left: Pairwise RMSD of C10 backbone and **1c** heavy atoms. Right: Corresponding atomic fluctuations summarized by C10 residues as their mass-weighted average. Orange dots correspond to C10 residues establishing contacts with the dodecaamide **1c**.

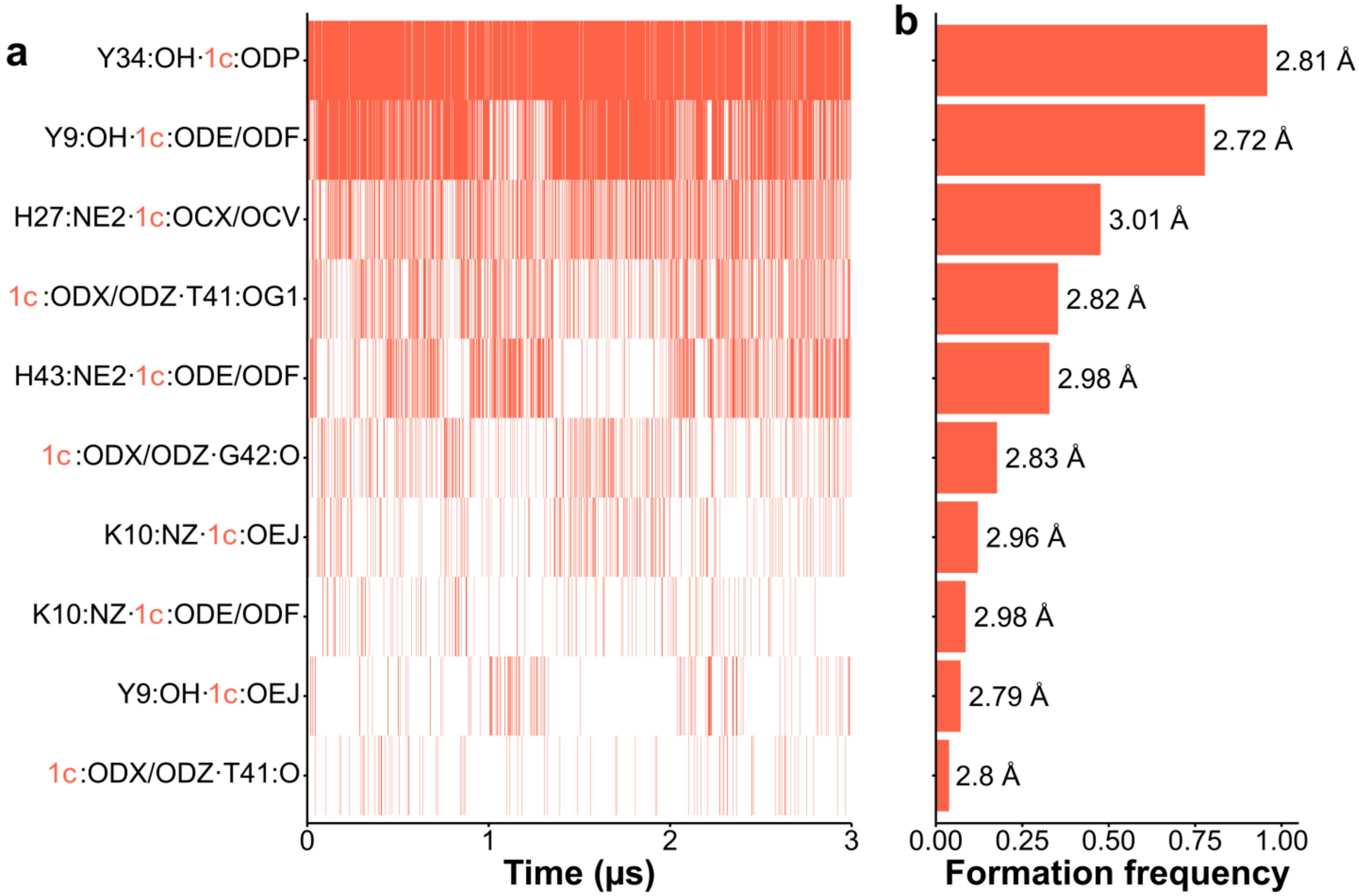

Supplementary Figure 9 |Analysis of intermolecular hydrogen bonding over the course of the MD simulation of the 1c•C10 complex.

a H-bonding between C10 and **1c** across 3 microseconds of production MD. H-bonding event (colored frames; 3.2 Å, 135° cut-offs). Note that the atom numbering is consistent with the deposited NMR structure ensemble. **b** Corresponding H-bonding frequencies, labelled with the mean H-bond length (when formed). Note that equivalent atoms were grouped.

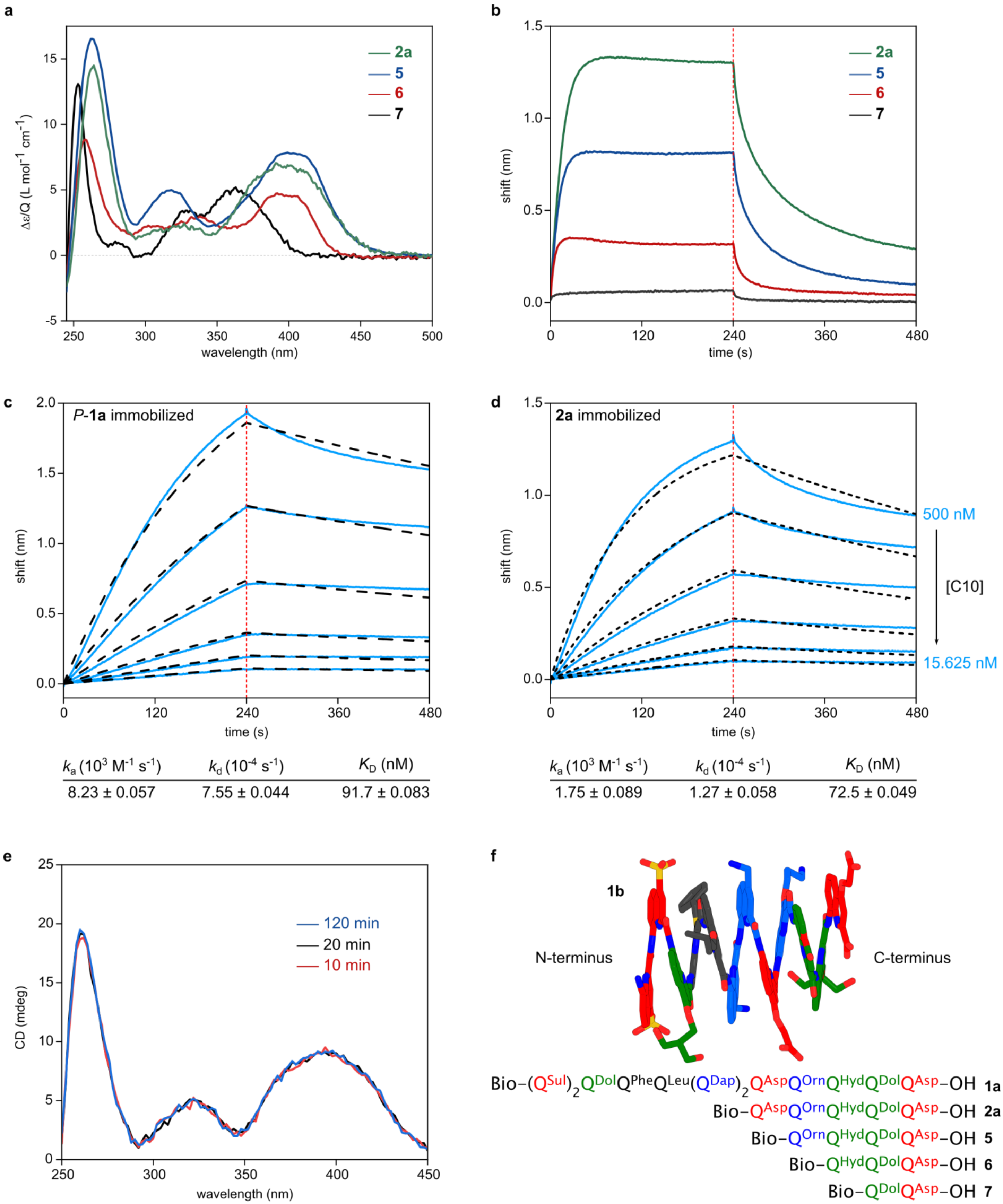

k_a (10^3 M^{-1} s^{-1})	k_d (10^{-4} s^{-1})	K_D (nM)
8.23 ± 0.057	7.55 ± 0.044	91.7 ± 0.083

k_a (10^3 M^{-1} s^{-1})	k_d (10^{-4} s^{-1})	K_D (nM)
1.75 ± 0.089	1.27 ± 0.058	72.5 ± 0.049

Supplementary Figure 10 | Solution phase binding assessment of AOFs towards C10.

a CD spectra of truncated versions of **1a** at 50 µM in presence of C10 (1 equiv.). Sequences **2a**, **5**, **6** and **7** carry the last five, four, three, and two C-terminal Q^{Xxx} units of the selection target dodecaamide **1a**. Samples were

incubated for three days at room temperature at 50 µM to ensure full handedness induction of longer sequence **2a**. Before mixing with the protein, none of the foldamers showed CD signals, confirming no influence of the chiral biotin moiety on helix handedness. Spectra were normalized to ε per quinoline. It is interesting to note that at 50 µM, i.e. a concentration much above the K_D of **1a**, even the triamide **6** and the diamide **7** showed some binding reflected in the induced CD bands. **b** BLI sensorgrams of single concentration binding assays of **2a**, **5**, **6** and **7** against 250 nM of C10. The red dashed line indicates the start of the dissociation step. Binding strength may not be inferred directly from the intensity of the signal as this also depends on the amount of foldamer loaded on the streptavidin-coated sensor. In addition, the handedness of the foldamer is expected to be dynamic in the time course of the course of the 120 s long association phase for shorter oligomers **5**, **6** and **7**, resulting in an enrichment of *P* helices upon C10 binding, whereas the handedness of pentaamide **2a** will remain essentially unchanged in this short time at 25°C. A K_D of ca. 120 nM was calculated for **5**. Data were not accurate enough for **6** and **7** and their K_D values were estimated to be > 1 µM. **c-d** Kinetic BLI measurements of **1a** (**c**) and **2a** (**d**) against a serial dilution of C10 from 500 nM to 15.625 nM. Curve fits are displayed as black dashed lines. **e** CD spectra of **2a** in complex with C10 at 50 µM, recorded at 60°C over the course of 120 min. **f** Sequences of the selection target **1a** and its derived N-terminally truncated derivatives synthesized after the elucidation of the **1c**•C10 NMR structure. Atop, the crystal structure of *P*-**1b** is shown. Monomers are colored according to Fig. 1.

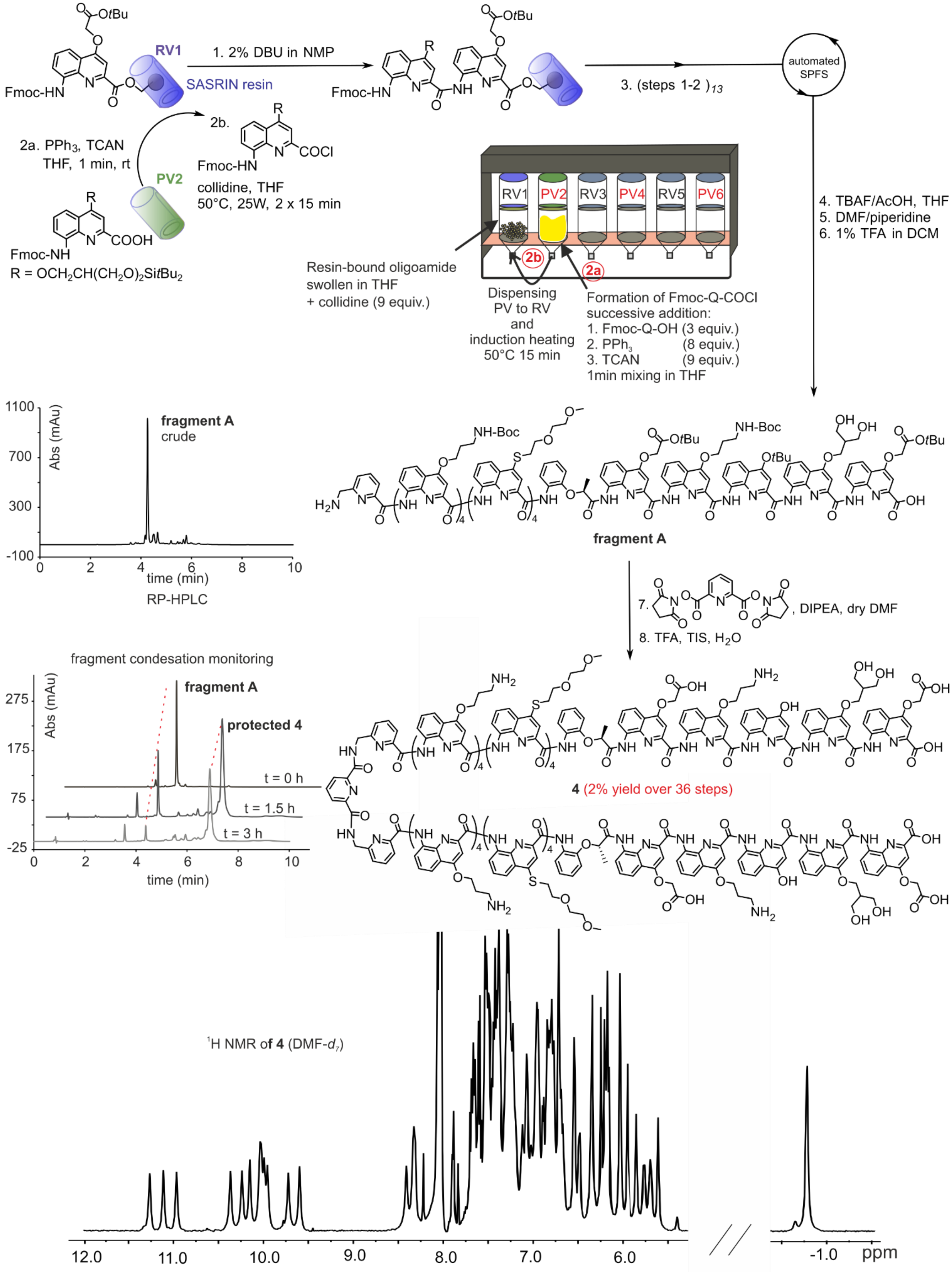

Supplementary Figure 11 | Synthetic pathway towards the *C*2 symmetrical 31mer 4 bearing two C10 binding epitopes including selected analytics for progress monitoring.

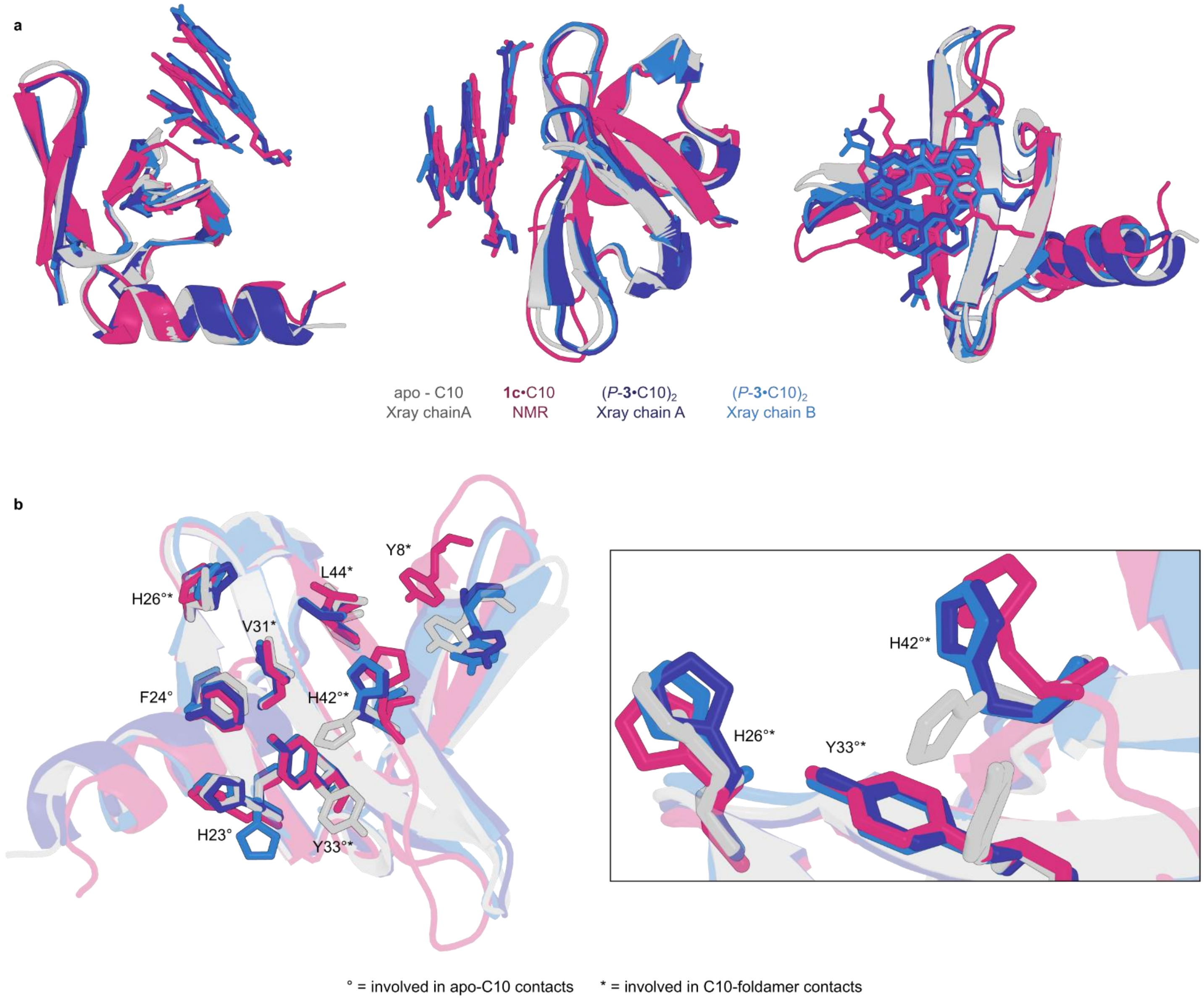

Supplementary Figure 12 | Comparison of apo and foldamer-bound C10 structures obtained by NMR and Xray crystallography.

a Three different views of the **1c**•C10 NMR structure (pink) and chains A (skyblue) and B (darkblue) of the (***P*-3**•C10)$_2$ crystal structure aligned to the C10 apo crystal structure (grey). The foldamers were truncated to the cognate, C-terminal pentameric binding epitope. The NMR structure file used here is #17 of the 20 exported states. **b** Highlighted residues involved in protein-protein contacts observed in the apo-C10 structure marked with a circle (°), those found across the elucidated protein-foldamer interfaces marked with a star (*). Residues found to be involved in both types of interfaces are shown in a boxed closeup.

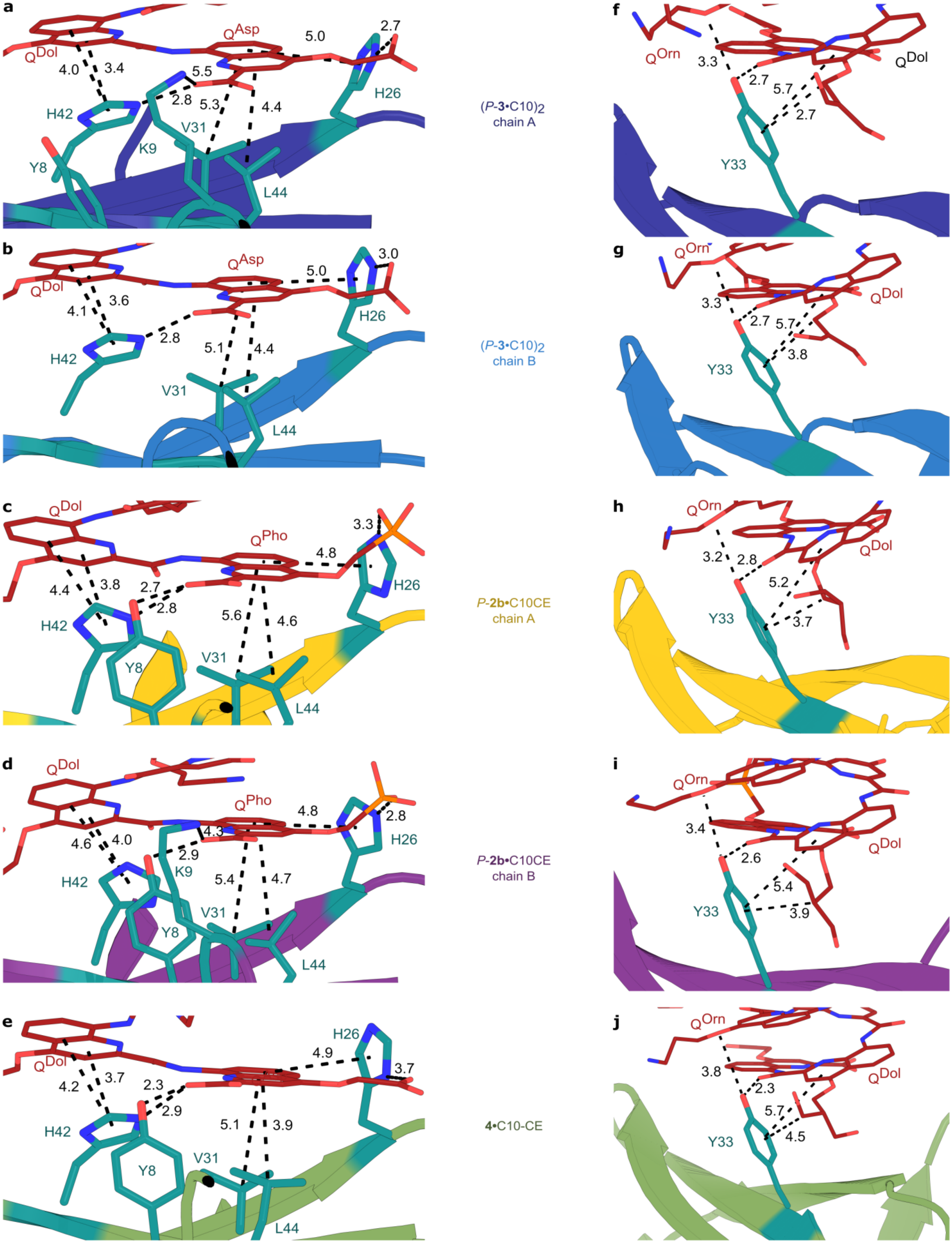

Supplementary Figure 13 part 1

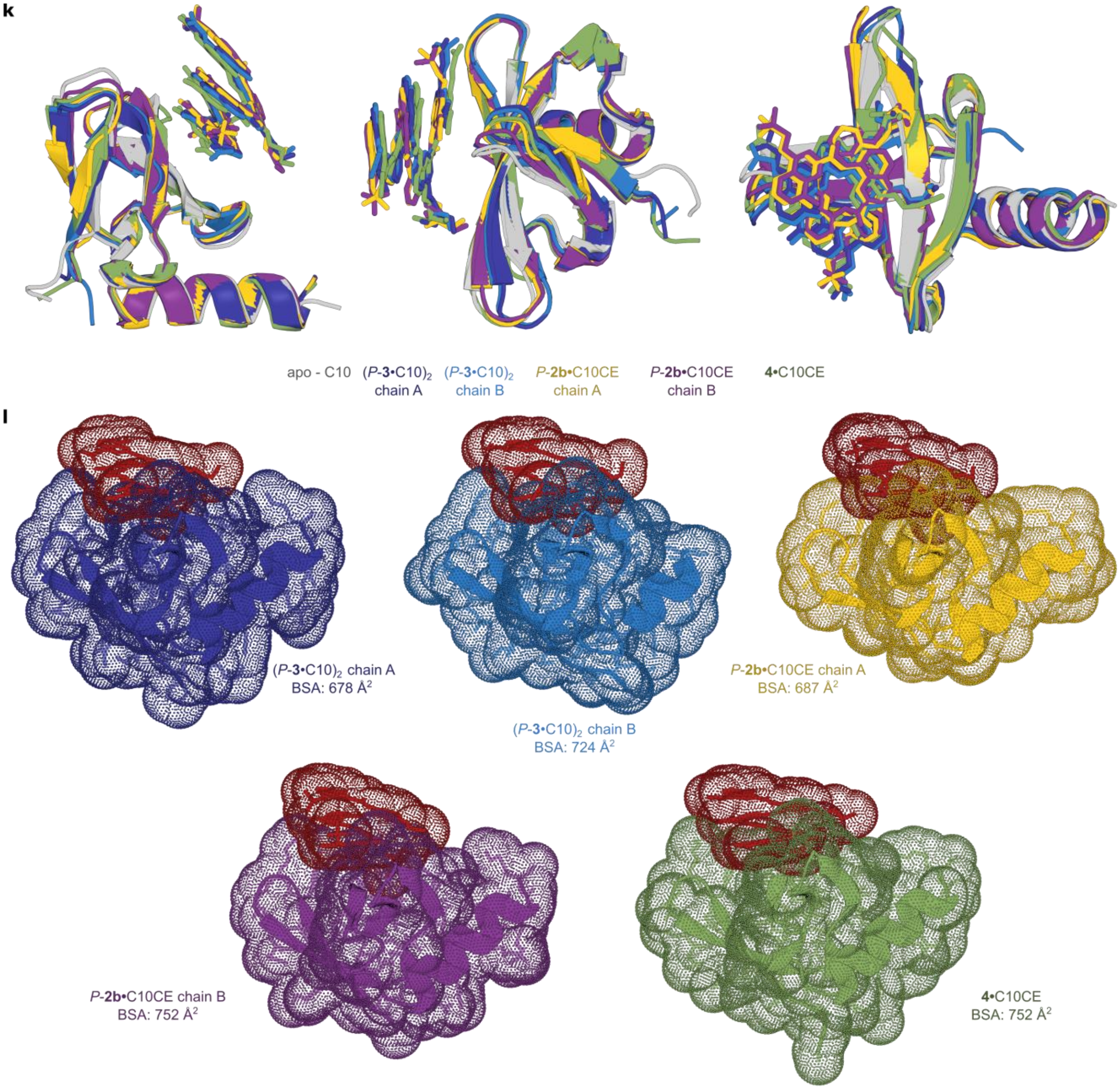

Supplementary Figure 13 part 2 | Conservation of the C10-foldamer binding interface across five observed instances in three different crystal structures.

a-**e** Key interactions and contacts between C10 residues (teal) and the foldamer identified binding epitope (red) observed in five different interfaces extracted from three complex crystal structures highlighted. The views are focused on the C-terminal unit of each foldamer. Distances are given in Å. **f**-**j** View on the interactions and contacts between the foldamers and C10, focused on the environment of Y33 and the second unit (Q^{Dol}) and fourth unit (Q^{Orn}) of the foldamers counting from the foldamers C-termini. In **a**-**j** perspectives were adjusted to accommodate labels. Contacts are listed in Supplementary Table 5. **k** Alignment of all five C10-foldamer complexes to the apo-C10 structure from different perspectives. Foldamer modifications beyond the C-terminal pentaamide and protein coiled-coil extensions were omitted to visually match the common binding epitope and provide clarity. **l** Illustration of the buried surface area (BSA) in the five cognate C10-foldamer complexes. The solvent accessible surface area (SASA) is shown for each compound individually in dot representation (foldamer in red, C10 following the color scheme introduced in (A)). BSA for each complex was approximated according to BSA = [SASA(C10) + SASA(foldamer)] – SASA(complex. All values are listed in Table S5.

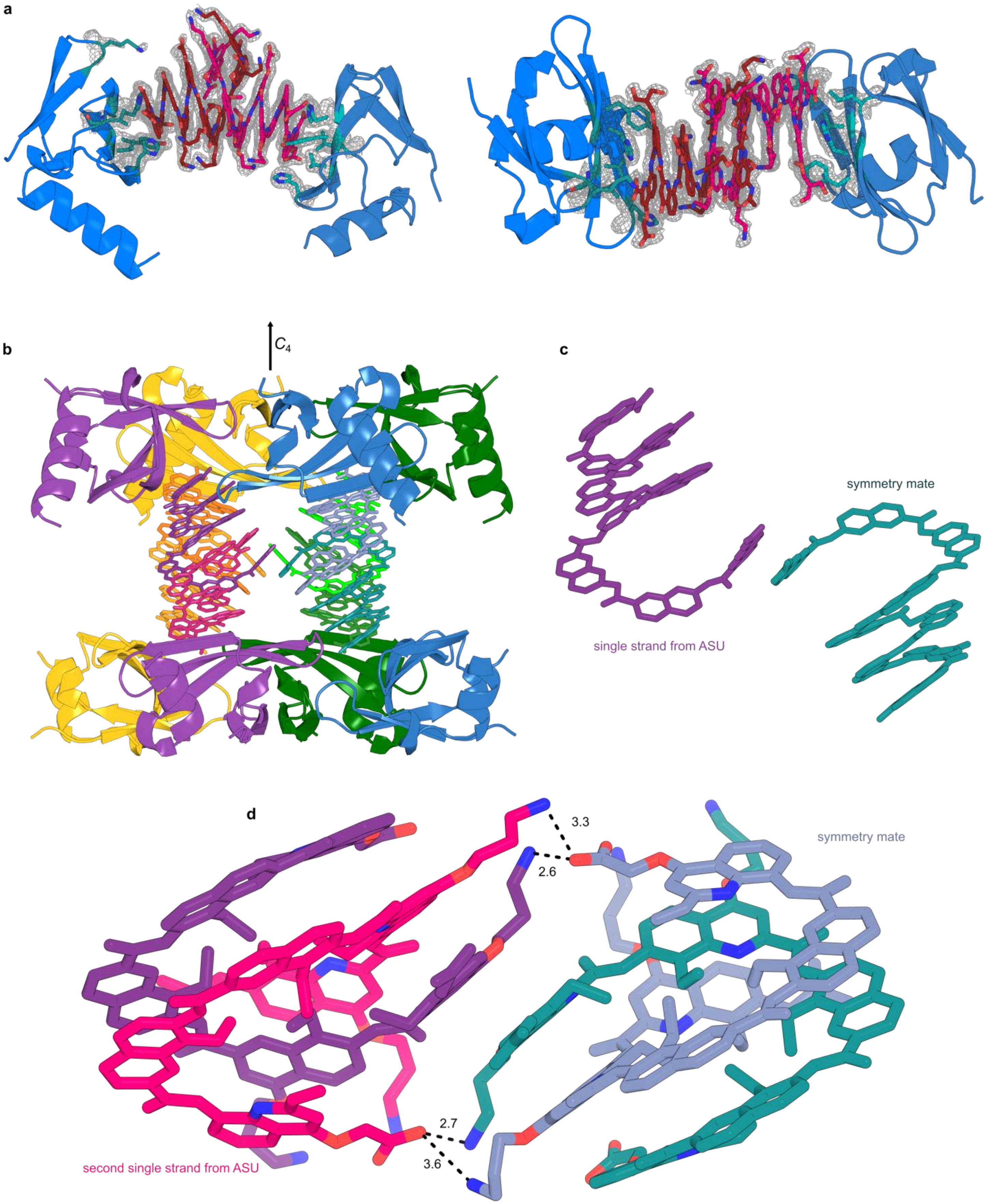

Supplementary Figure 14 part 1

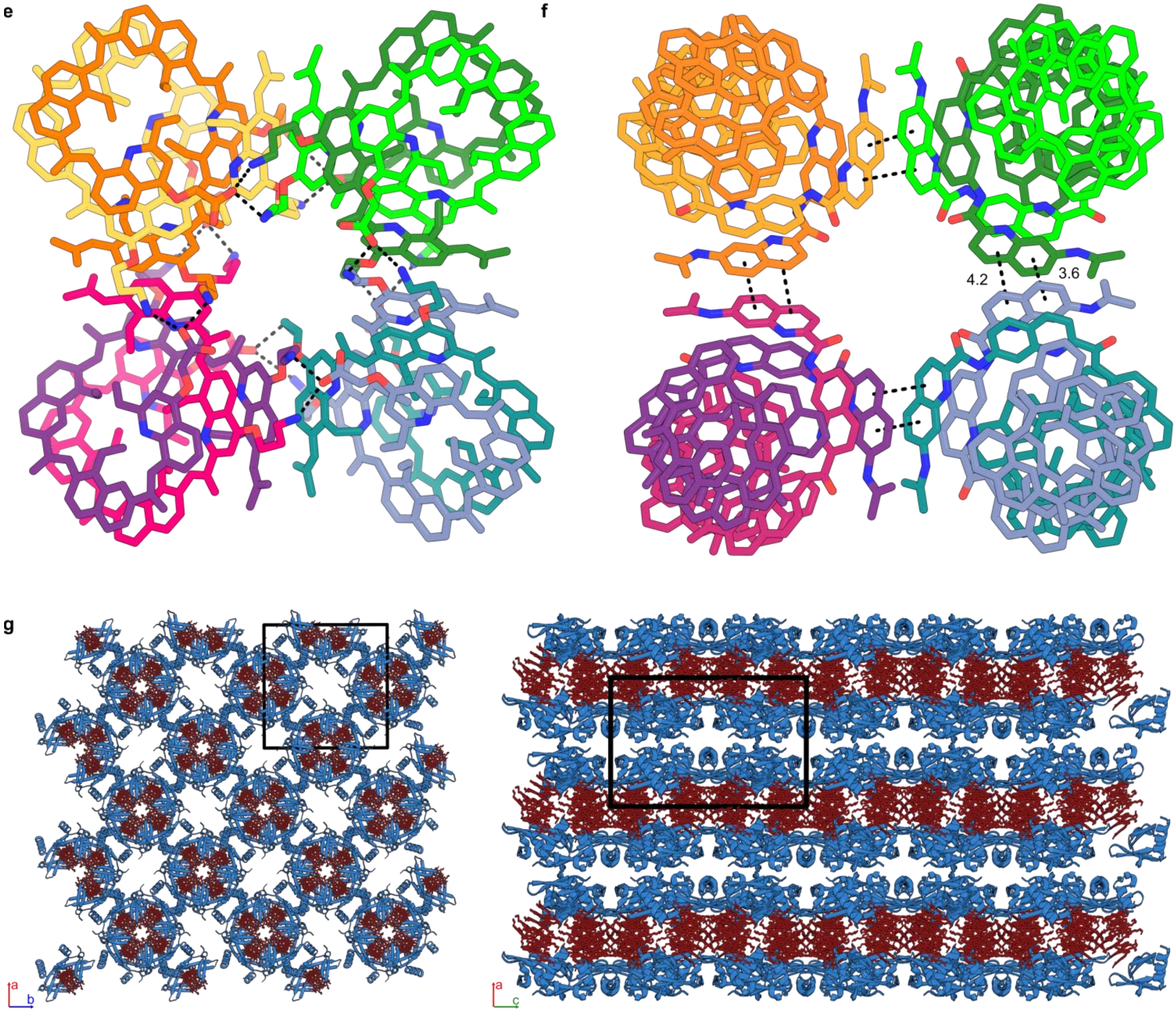

Supplementary Figure 14 part 2 | Extended view of the (*P*-3•C10)$_2$ complex crystal structure

a ASU of the crystal structure shown from two perspectives with electron density map (2Fo-Fc) around the foldamer contoured at 1 σ. **b** Cyclic 8+8 aggregate comprised of four (*P*-**3**•C10)$_2$ complexes found in the crystal packing. **c** Arrangement of two symmetry-equivalent foldamer single strands from two protein double-helix complexes. The terminal Q_{OMe} units of each strand tilt out of the expected helical array to establish face-to-face π-π contacts with the neighboring oligomer. **d**-**f** Detailed views of the interconnection between symmetry equivalent (*P*-**3**•C10)$_2$ complexes, establishing extensive interduplex π-π stacking across the eight individual single strands as well as interduplex charge-reinforced hydrogen bonds between the side chains of Q^{Asp} and Q_{OMe}. For every duplex-duplex contact, each duplex acts as a hydrogen bond donor towards the other duplex through two of its Q_{OMe} sidechains and, at the same time, as a hydrogen bond acceptor towards both strands of the other duplex through one of its Q^{Asp} sidechain. The intertwined network of local contacts gives rise to the symmetrical 8+8 aggregate. Relevant contacts are marked with dashed lines distances are annotated in Å. (**G**) Two views of the crystal packing, along (001) and (010). The 8+8 aggregates build a sheet-like lattice, stacking on top of each other and connect laterally through protein-protein contacts. The unit cell is outlined in black.

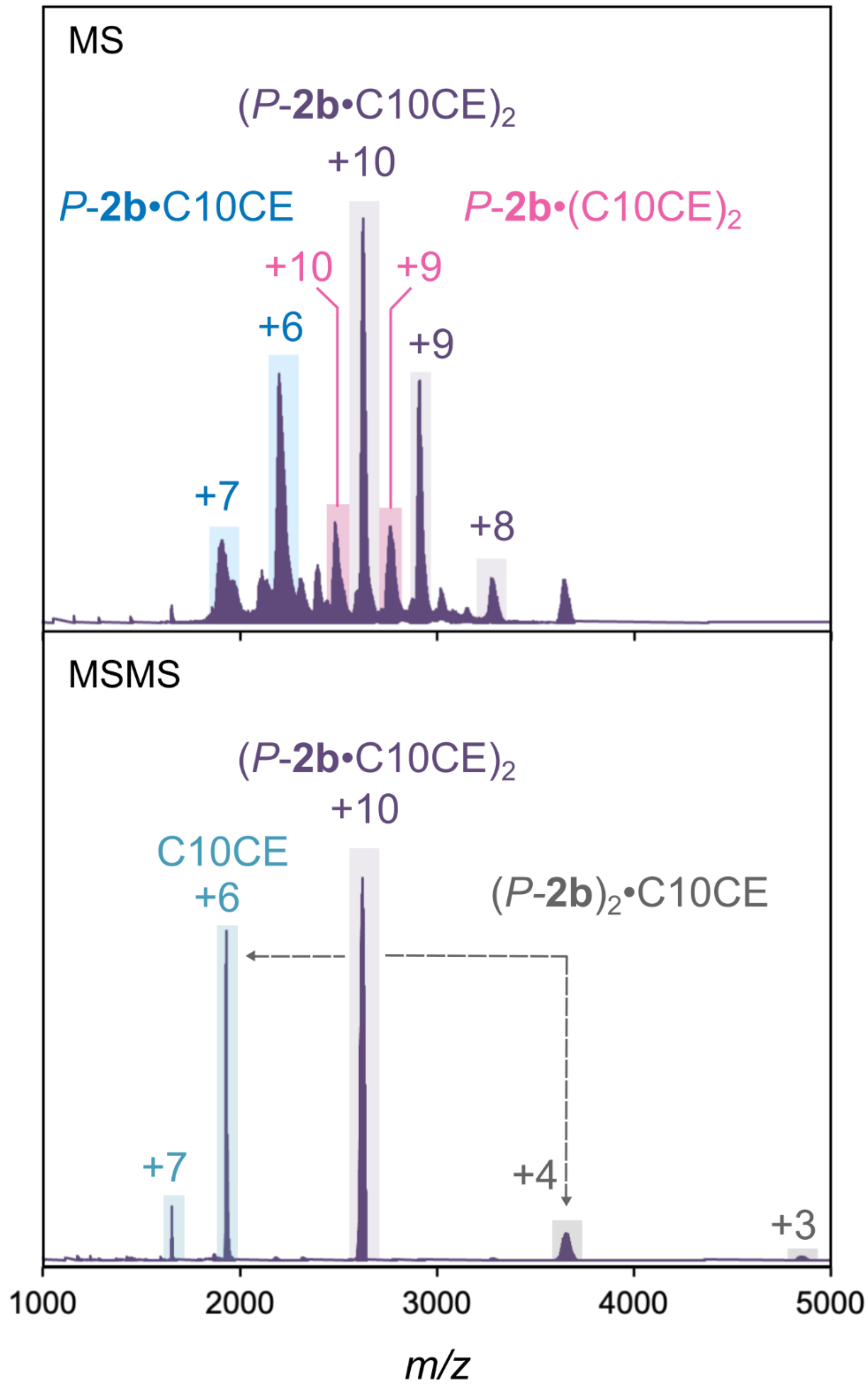

Supplementary Figure 15 | Native nESI-MS and MSMS of the C10CE protein in complex with 2b. Conditions: 100 µM of both C10CE and 2b in 200 mM ammonium acetate solution pH 6.8.

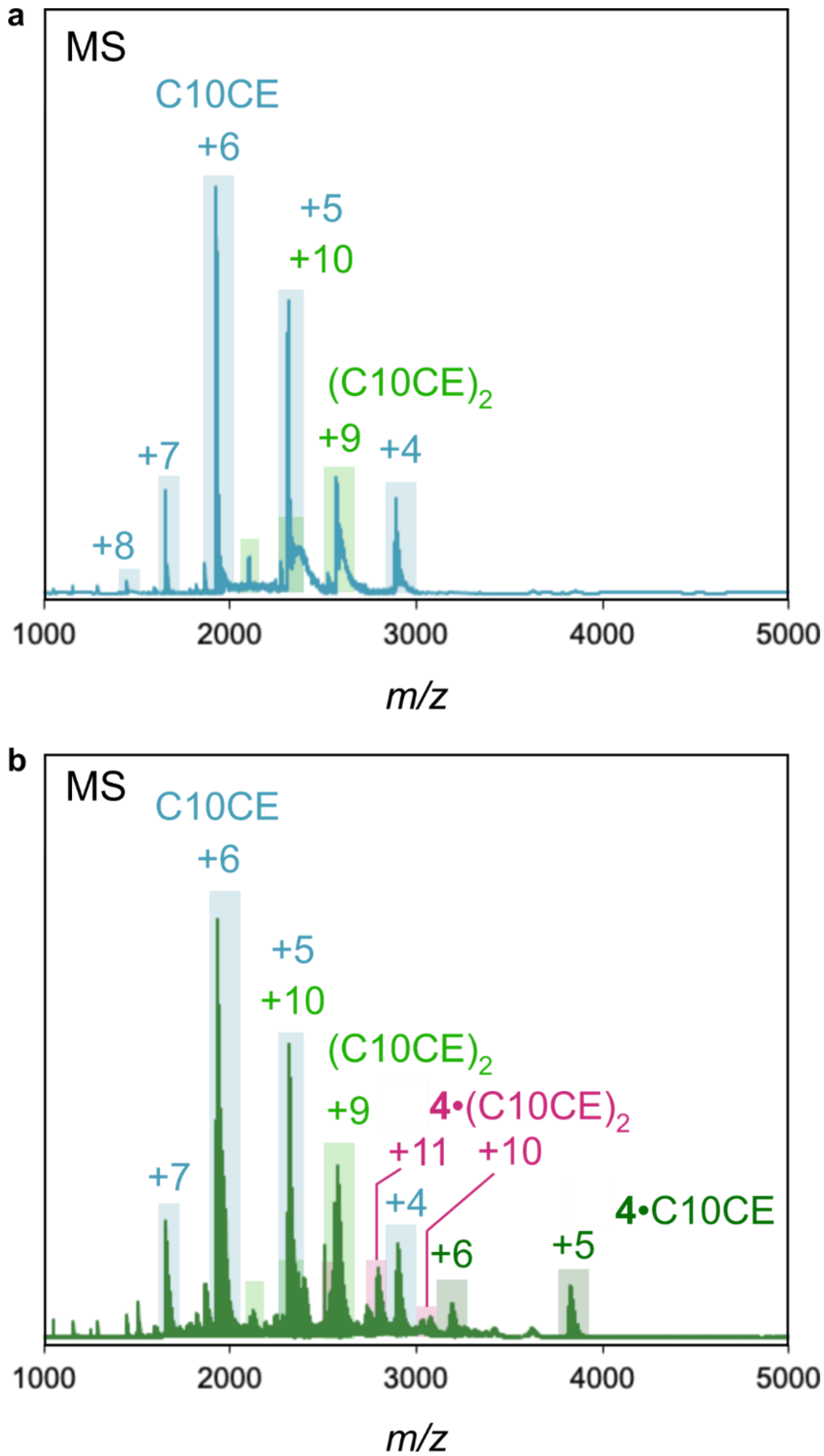

Supplementary Figure 16 | Native nESI-MS of the C10CE protein alone (a) and in complex with the 31 unit-long AOF 4 (b).

Conditions: 100 µM of both C10CE and 4 in 200 mM ammonium acetate solution pH 6.8.

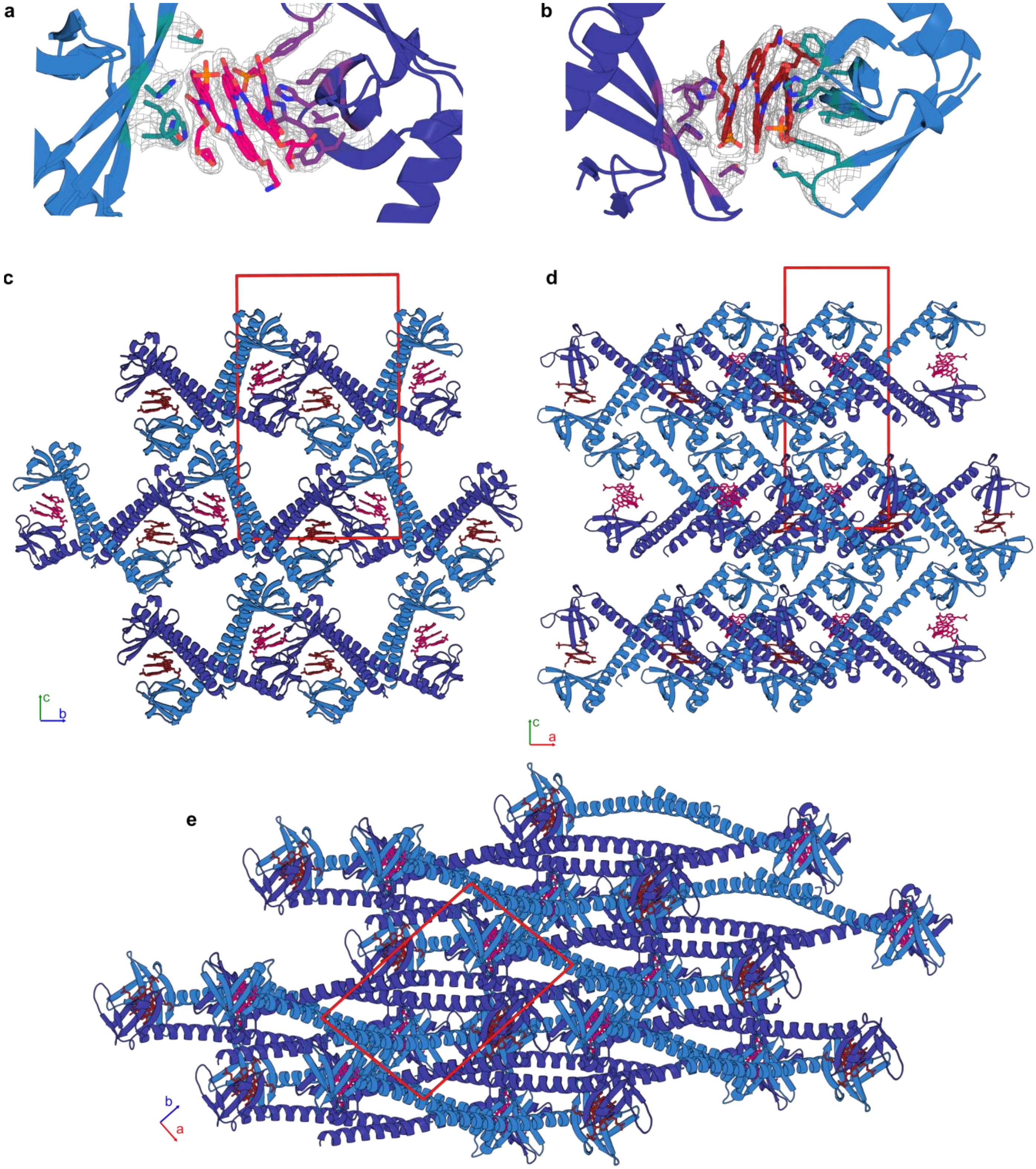

Supplementary Figure 17 | Extended views of the crystal structure of the coiled-coil extended C10 (C10CE) in complex with pentaamide *P*-2b.

a-**b** Highlighted cognate and non-cognate binding between C10 and the foldamer*P*-**2b** in the two occurrences in the ASU. The cognate interface between the C-terminal cross-section of **2b** and chain A (dark-blue) is shown in **a**, and its equivalent instance involving chain C (sky-blue) is shown in **b** respectively. The electron density maps (2Fo-Fc) around the foldamer and protein sidechains of interest are contoured at 1 σ. **c-e** Views on the crystal packing along (100), (010) and (001), with the unit-cell outlined in red.

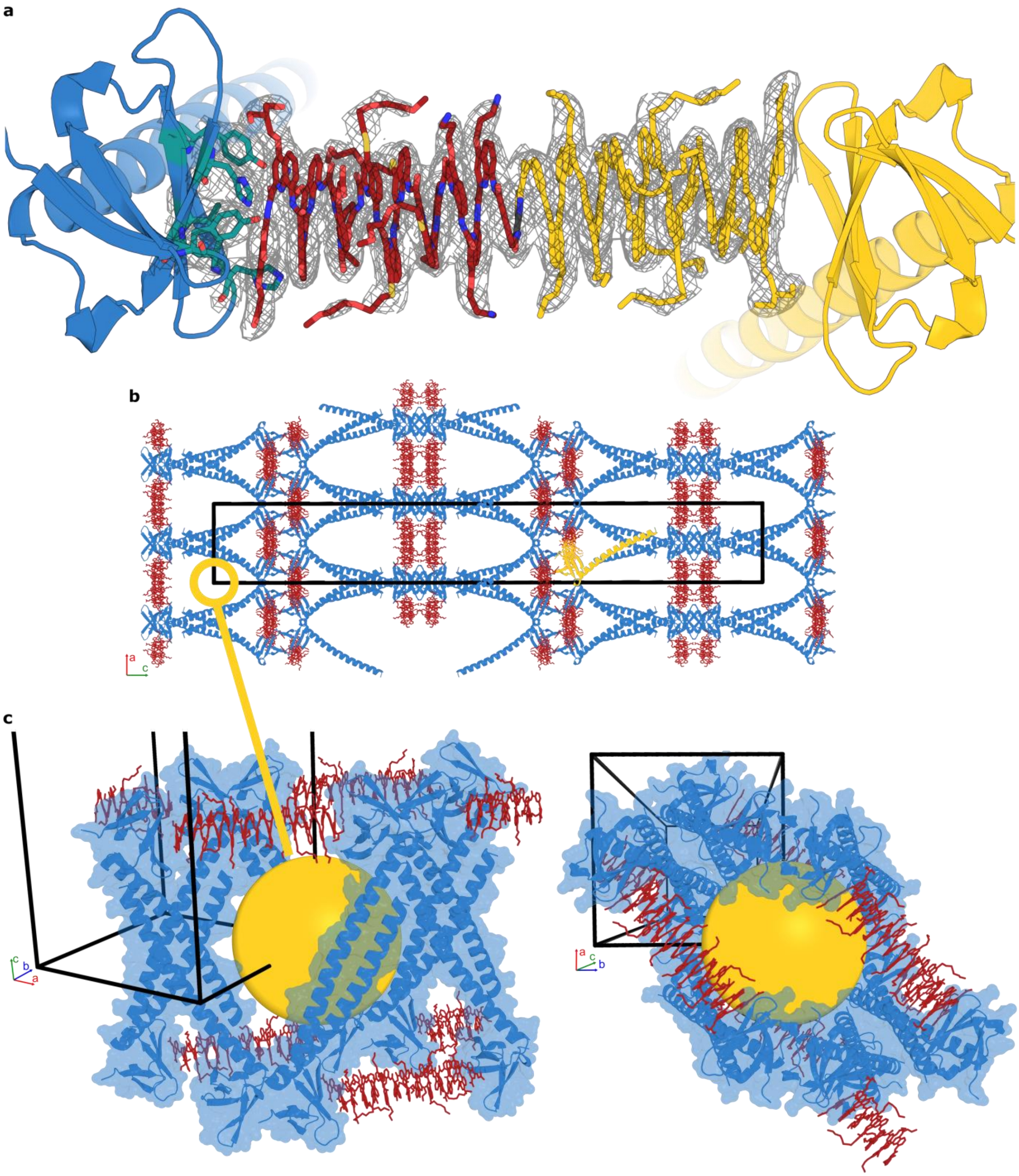

Supplementary Figure 18 | Additional views on the crystal structure of 4•C10-CE.

a Close-up of the foldamer mediated protein dimerization. One ASU is highlighted in gold. The crystallographic C_2 axes coincides with the local symmetry axis of the linker unit D. The electron density map (2Fo-Fc) is shown around the foldamer and interacting protein sidechains at 1 σ **b** View of the crystal lattice along (010) showing the largest solvent channels. **c** A sphere of 5 nm diameter rendered in the center of the largest cavity at the origin.

3. Supplementary Tables

Supplementary Table 1 | X-ray crystallography data collection and refinement statistics. Values in brackets refer to high resolution shells.

**Molecule**	**C10 coiled-coil**	***P*-2b•C10CE**	**(*P*-3•C10)$_2$**	**4•C10CE**
X-ray source / wavelength (Å)	EMBL-P13 0.8000	EMBL-P14 0.9763	EMBL-P13 0.8731	EMBL-P13 0.8266
Resolution (Å)	15.5 - 1.4 (1.45 - 1.4)	64.66 - 2.8 (3.02 - 2.8)	17.27 - 1.85 (1.916 - 1.85)	16.38 - 2.53 (2.62 - 2.53)
Space group	P 21 21 21	P 1 21 1	P 4 21 2	I 41 2 2
Unit cell				
a, *b*, *c* (Å)	42.59, 62.96, 89.29	42.46, 105.54, 65.52	85.92, 85.92 56.57	56.91 56.91 394.87
α, *β*, *γ* (°)	90, 90, 90	90 99.3 90	90, 90, 90	90, 90, 90
Total reflections	307818 (30530)	82596 (12605)	484652 (50104)	286070 (29603)
Unique reflections	47019 (4575)	13610 (2420)	18595 (1816)	11489 (1105)
Multiplicity	6.5 (6.7)	6.1 (5.2)	26.1 (27.6)	24.9 (26.8)
Completeness (%)	97.80 (97.40)	96.46 (86.14)	99.71 (99.94)	98.59 (99.64)
Mean I/σ(I)	23.09 (2.74)	15.87 (3.34)	11.55 (2.18)	16.24 (2.25)
Wilson B-factor	16.26	58.92	28.28	71.02
Rmerge (%)	0.04501 (0.4376)	0.06526 (0.3697)	0.1933 (1.614)	0.1269 (1.684)
CC1/2 (%)	0.998 (0.919)	0.999 (0.954)	0.998 (0.746)	0.998 (0.846)
CC* (%)	1 (0.979)	1 (0.988)	0.999 (0.925)	1 (0.957)
ASU content	1 C10 coiled-coil dimer	2 C10CE dimers + 2 *P*-**2b**	1 **(***P***-3•**C10**)$_2$**	1 C10CE monomer + ½ **4**

**Refinement**				
Reflections used in refinement	47019 (4575)	13583 (2411)	18592 (1816)	11402 (1103)
Reflections used for R_{free}	2000 (195)	680 (121)	1860 (182)	1140 (111)
R_{work}	0.1774(0.2156)	0.2603 (0.3759)	0.1870 (0.2475)	0.2441 (0.3992)
R_{free}	0.1976(0.2578)	0.3064 (0.4459)	0.2289 (0.2896)	0.2699 (0.4887)
RMS bonds (Å)	0.006	0.013	0.353	0.003
RMS angles (°)	0.87	1.63	2.87	0.66
Protein residues	198	382	128	98
No. non H atoms	2169	2949	1610	1063
macromolecules	1678	2745	1069	1063
ligands	0	204	383	790
solvent	491	0	177	273
Average B factor	21.54	66.41	35.16	84.16
PDB code	9QDO	9QT7	9QOG	9QNU

Supplementary Table 2 | Atom equivalence and 1H chemical shift assignment of C10-bound foldamer 1c.

Data collected at 298 K. Nomenclature is detailed in Supplementary Fig. 6. Methylene protons are indicated with an asterisk (*) and are not stereospecifcally assigned. Methyl protons are annoted with an *m*.

Monomer unit	name	AMBER name	chemical shift /ppm
camphanyl	H3 *	HXA, HXB	1.77, 1.32
	H4 *	HXC, HXD	0.98, 1.58
	H8 *m*	HXE, HXF, HXG	-0.17
	H9 *m*	HXH, HXI, HXJ	0.43
	H10 *m*	HXK, HXL, HXM	0.39
Q1 / Q^{Sul}	HN-1	HXN	9.19
	H3	HYA	-
	H5	HXT	8.11
	H6	HXS	7.38
	H7	HXR	7.41
Q2 / Q^{Sul}	HN-2	HYE	10.74
	H3	HYR	7.8
	H5	HYK	-
	H6	HYJ	-
	H7	HYI	-
Q3 / Q^{Dol}	HN-3	HYV	10.8
	H3	HZM	5.9
	H5	HZB	7.76
	H6	HZA	7.27
	H7	HYZ	7.64
	H2'*	HZF, HZG	4.20, 4.09
	H3'	HZH	2.45
	H4a'*	HZK, HZL	3.98, 3.98
	H4b'*	HZI, HZJ	3.98, 3.98
Q4 / Q^{Phe}	HN-4	HAE	10.49
	H3	HAV	6.32
	H5	HAK	7.74
	H6	HAJ	7.39
	H7	HAI	7.11
	H2' *	HAO, HAP	4.21, 4.27
	H4' *	HAQ, HAU	-
	H5' *	HAR, HAT	-
	H6'	HAS	-
Q5 / Q^{Leu}	HN-5	HAZ	10.41
	H3	HBS	5.89
	H5	HBF	7.92
	H6	HBE	7.38
	H7	HBD	7.04
	H2' *	HBJ, HBK	4.12, 3.95
	H3'	HBL	2.31
	H4a' *m*	HBM, HBN, HBO	1.245
	H4b' *m*	HBP, HBQ, HBR	1.245
Q6 / Q^{Dap}	HN-6	HGO	10.3
	H3	HCC	6.59
	H5	HBX	7.73
	H6	HBW	7.45
	H7	HBV	7.22
	H1' *	HCA, HCB	4.40, 4.45

Monomer unit	name	AMBER name	chemical shift /ppm
Q7 /Q^{Dap}	HN-7	HCG	10.21
	H3	HCR	6.62
	H5	HCK	7.58
	H6	HCJ	6.84
	H7	HCI	6.82
	H1' *	HCT, HQT	4.45, 4.54
Q8 / Q^{Asp}	HN-8	HFV	10.37
	H3	HFR	5.93
	H5	HFY	7.87
	H6	HFX	7.26
	H7	HFW	6.97
	H2' *	HGC, HGD	4.59, 4.59
Q9 / Q^{Orn}	HN-9	HFN	10.54
	H3	HEZ	5.99
	H5	HFJ	7.76
	H6	HFK	7.12
	H7	HFL	6.77
	H2' *	HFC, HFD	4.20, 4.20
	H3' *	HFE, HFF	2.31, 2.45
	H4' *	HFG, HFH	3.14, 3.14
Q10 / Q^{Hyd}	HN-10	HEV	10.38
	H3	HEL	6.05
	H5	HER	7.61
	H6	HES	6.93
	H7	HET	6.85
Q11 / Q^{Dol}	HN-11	HEH	11.02
	H3	HDR	5.78
	H5	HED	7.55
	H6	HEE	7.42
	H7	HEF	7.96
	H2'*	HDU, HDV	2.21, 2.21
	H3'	HDW	1.82
	H4a' */<br>H4b' *	HDX, HDY,<br>HDZ, HD0	3.48, 3.37,<br>3.21, 3.35
Q12 / Q^{Asp}	HN-12	HDN	10.87
	H3	HDB	6.37
	H5	HDJ	6.68
	H6	HDK	6.93
	H7	HDL	8.03
	H2' *	HCY, HCZ	4.33, 4.14

Supplementary Table 3 | NMR and refinement statistics for foldamer-bound Nanofitin C10.
ARIA/CNS followed by AMBER. Final statistics calculated over all 20 models.

**ARIA/CNS**	
Distance restraints	
Total	737
Intra-residue	190
Sequential (|*i* - *j*|) = 1	186
Short (|*i* - *j*|) > 1	65
Medium (|*i* - *j*|) > 1	24
Long (|*i* - *j*|) > 1	104
Foldamer	148
Intermolecular	20
Ambiguous [a]	113
Dihedral angle restraints	
phi	57
psi	57
**AMBER** [b]	
**Structure statistics**	
Violations (mean and s.d.)	
Number of distance violations (> 0.3 Å)	6 ± 1.0
Number of distance violations (> 0.5 Å)	0
Max distance violation (Å)	0.43 ± 0.02
Number of dihedral angle violations	0
Average pairwise r.m.s. deviation (mean and s.d.)	
Backbone protein heavy atoms (Å)	1.1 ± 0.2
All protein heavy atoms (Å)	1.7 ± 0.2
All foldamer heavy atoms (Å)	1.0 ± 0.2
Backbone protein and foldamer heavy atoms (Å)	1.3 ± 0.2
All heavy atoms (Å)	1.7 ± 0.2

[a] Class of distance restraints in Aria2.3 for crosspeak volumes contributed by two or more overlapping NOE
[b] Only the 737 non-ambiguous distance restraints obtained by using ARIA/CNS were used for the AMBER minimization protocol, along with the 114 dihedral restraints

Supplementary Table 4 | Intermolecular NMR distance restraints for 1c•C10 from ARIA/CNS.

C10		Q12	Lower Bound (Å)	Upper Bound (Å)
10	HE2	HEL	1.877	4.145
10	HE1	HEL		
25	HE1	HFJ	1.928	4.558
25	HE2	HFJ		
25	HE1	HFE	1.997	5.709
25	HE2	HFE		
30	HD22	HDB	1.970	5.040
30	HD21	HDB		
30	HD23	HDB		
30	HD12	HDB	1.999	5.771
30	HD11	HDB		
30	HD13	HDB		
32	HG12	HDK	1.967	4.993
32	HG11	HDK		
32	HG13	HDK		
32	HG12	HDL	1.893	4.251
32	HG11	HDL		
32	HG13	HDL		
32	HG22	HDJ	1.962	4.928
32	HG21	HDJ		
32	HG23	HDJ		
32	HG22	HDK	1.907	4.369
32	HG21	HDK		
32	HG23	HDK		
32	HG22	HDL	1.959	4.887
32	HG21	HDL		
32	HG23	HDL		
41	HG21	HDX	1.905	4.351
41	HG21	HDY		
41	HG21	HDZ		
41	HG21	HD0		
41	HG22	HDX		
41	HG22	HDY		
41	HG22	HDZ		
41	HG22	HD0		
41	HG23	HDX		
41	HG23	HDY		
41	HG23	HDZ		
41	HG23	HD0		
43	HD2	HFN	1.981	5.229
43	HD2	HDN	1.980	5.214
45	HD12	HDB	1.946	4.746
45	HD11	HDB		
45	HD13	HDB		
45	HD22	HDK	1.998	5.732
45	HD21	HDK		
45	HD23	HDK		
45	HD22	HDJ	1.990	5.442
45	HD21	HDJ		
45	HD23	HDJ		
25	HZ	HDL	1.786	3.598
25	HE1	HDL	1.803	3.687
25	HE2	HDL		
27	HE1	HDL	1.903	4.335
45	HD22	HDB	1.913	4.413
45	HD21	HDB		
45	HD23	HDB		

Supplementary Table 5 | Comparison of the cognate C10-foldamer binding interface across all observed crystal structures.

The table complements Supplementary Fig. 13 listing the C10-foldamer contacts and interactions illustrated. Distances are given in Å. A dash indicates a missing contact. *C-term.* refers to the C-terminal carboxylate function of the foldamers. *Pyridine* and *benzene* indicate the two condensed rings in a quinoline. To approximate the binding interface area, hence the buried surface area (BSA), the solvent accessible surface area (SASA) of the complex was subtracted from the sum of the individually calculated SASAs of the protein and foldamer (BSA = [SASA(C10) + SASA(foldamer)] – SASA(complex)). Values were calculated via the *get_sasa* function in Pymol.

**C10 residue**	**Foldamer site**	**(*P*-3•C10)$_2$ Chain A**	**(*P*-3•C10)$_2$ Chain B**	***P*-2b•C10-CE Chain A**	***P*-2b•C10-CE Chain B**	**4•C10-CE**
Y8	$Q^{Asp/Pho}$ C-term.	-	-	2.7	2.9	2.3
H42	$Q^{Asp/Pho}$ C-term.	2.8	2.8	2.8	-*	2.9
L44	$Q^{Asp/Pho}$ pyridine	4.4	4.4	4.6	4.7	3.9
V31	$Q^{Asp/Pho}$ benzene	5.3	5.1	5.6	5.4	5.1
H26	$Q^{Asp/Pho}$ benzene	5.0	5.0	4.8	4.8	4.9
H26	$Q^{Asp/Pho}$ sidechain	2.7	3.0	3.3	2.8	3.7
Y33	$Q^{Asp/Pho}$–Q^{Dol} amide	2.7	2.7	2.8	2.6	2.3
H42	Q^{Dol} pyridine	3.4	3.6	4.0	4.0	3.7
Y33	Q^{Dol} pyridine	5.7	5.7	5.2	5.4	5.7
H42	Q^{Dol} benzene	4.0	4.1	4.6	4.6	4.2
Y33	Q^{Dol} sidechain	3.7	3.8	3.7	3.9	4.5
Y33	Q^{Orn} sidechain O	3.3	3.3	3.2	3.4	3.8
**BSA interface (Å^2)**		678	724	687	752	752
**SASA(C10) (Å^2)**		4749	4244	4176	4460	4898
**SASA (foldamer) (Å^2)**		1150	1157	1182	1176	1169
**SASA (complex) (Å^2)**		5221	4677	4671	4884	5315

*real space refinement of this residue was ambiguous, the side chain may be flipped to establish a 3.0 A contact to the foldamer's C-terminal carboxylate.

4. **Supplementary Videos**

Supplementary Video 1 (separate file). **MD simulation of the 1c•C10 complex.**

Movie of the restrained, production MD of the **1c**•C10 complex (3-µs, step: 10 ps), where **1c** is shown in pink and C10 in blue, with binding site residues Y8, K9, H26, Y33, T40, G41 and H42 shown as sticks. The solvent and salts were stripped for the sake of clarity.

Supplementary Video 2. (separate file). Animated view of the (*P*-3•C10)$_2$ complex.

The (*P*-**3**)$_2$ double helix and the whole (*P*-**3**•C10)$_2$ complex are shown successively.

Supplementary Video 3 (separate file) Animated view of the 8+8 assembly of four (*P*-3•C10)$_2$ complexes.

The four (*P*-**3**•C10)$_2$ complexes forming an 8+8 assembly appear one after the other. The movie ends with a close view of the stacking of Q_{OMe} units belonging to different (*P*-**3**)$_2$ duplexes.

Supplementary Video 4 (separate file). Animated view of the cyclic X-shape assembly of two foldamers *P*-2b and two C10 coiled-coil dimers.

Residues shown in dark blue belong to the cognate binding interface between C10 and the C-terminal cross section of *P*-**2b**. Residues shown in light blue belong to the non-cognate binding interface between C10 and the N-terminal cross section of *P*-**2b**.

5. Methods-only References

51 Hu, X. *et al.* Optimizing side chains for crystal growth from water: a case study of aromatic amide foldamers. *Chem. Sci.* **8**, 3741-3749 (2017). https://doi.org/10.1039/c7sc00430c

52 Baptiste, B. T., Douat-Casassus, C. L., Laxmi-Reddy, K., Godde, F. D. R. & Huc, I. Solid Phase Synthesis of Aromatic Oligoamides: Application to Helical Water-Soluble Foldamers. *J. Org, Chem.* **75**, 7175-7185 (2010). https://doi.org/10.1021/jo101360h

53 Vallade, M., Sai Reddy, P., Fischer, L. & Huc, I. Enhancing Aromatic Foldamer Helix Dynamics to Probe Interactions with Protein Surfaces. *Eur. J. Org. Chem.* **2018**, 5489-5498 (2018). https://doi.org/10.1002/ejoc.201800855

54 Teng, B. *et al.* Combining local conformational preferences and solvophobic effects in helical aromatic oligoamide foldamers. *Org. Biomol. Chem.* **21**, 3525-3530 (2023). https://doi.org/10.1039/D3OB00473B

55 Mazzier, D., De, S., Wicher, B., Maurizot, V. & Huc, I. Parallel Homochiral and Anti-Parallel Heterochiral Hydrogen-Bonding Interfaces in Multi-Helical Abiotic Foldamers. *Angew. Chem. Int. Ed.* **59**, 1606-1610 (2020). https://doi.org/10.1002/anie.201912805

56 Tsiamantas, C., Dawson, S. J. & Huc, I. Solid phase synthesis of oligoethylene glycol-functionalized quinolinecarboxamide foldamers with enhanced solubility properties. *C. R. Chim.* **19**, 132-142 (2016). https://doi.org/10.1016/j.crci.2015.06.007

57 Postma, T. M. *et al.* Dynamic Combinatorial Chemistry with Novel Dithiol Building Blocks: Towards New Structurally Diverse and Adaptive Screening Collections. *Synlett* **24**, 765-769 (2013). https://doi.org/10.1055/s-0032-1318407

58 Dengler, S., Mandal, P. K., Allmendinger, L., Douat, C. & Huc, I. Conformational interplay in hybrid peptide–helical aromatic foldamer macrocycles. *Chem. Sci.* **12**, 11004-11012 (2021). https://doi.org/10.1039/d1sc03640h

59 Chaudhuri, S. *et al.* Synthetic β-Cyclodextrin Dimers for Squaraine Binding: Effect of Host Architecture on Photophysical Properties, Aggregate Formation and Chemical Reactivity. *Eur. J. Org. Chem.* **2018**, 1964-1974 (2018). https://doi.org/10.1002/ejoc.201800283

60 Osterlund, N., Jordan, J. S., Renzi, E., Szekeres, G. P. & Pagel, K. Mass spectrometry detects folding intermediates populated during urea-induced protein denaturation. *Chem Sci* **16**, 22407-22416 (2025). https://doi.org/10.1039/d5sc05773f

61 Jordan, J. S., Xia, Z. & Williams, E. R. Tips on Making Tiny Tips: Secrets to Submicron Nanoelectrospray Emitters. *J Am Soc Mass Spectrom* **33**, 607-611 (2022). https://doi.org/10.1021/jasms.1c00372

62 Fort, K. L. *et al.* Expanding the structural analysis capabilities on an Orbitrap-based mass spectrometer for large macromolecular complexes. *Analyst* **143**, 100-105 (2017). https://doi.org/10.1039/c7an01629h

63 Thalassinos, K. *et al.* Characterization of phosphorylated peptides using traveling wave-based and drift cell ion mobility mass spectrometry. *Anal. Chem.* **81**, 248-254 (2009). https://doi.org/10.1021/ac801916h

64 Bush, M. F. *et al.* Collision cross sections of proteins and their complexes: a calibration framework and database for gas-phase structural biology. *Anal. Chem.* **82**, 9557-9565 (2010). https://doi.org/10.1021/ac1022953

65 Delaglio, F. *et al.* NMRPipe: A multidimensional spectral processing system based on UNIX pipes. *J. Biomol. NMR* **6** (1995). https://doi.org/10.1007/bf00197809

66 Lee, W., Tonelli, M. & Markley, J. L. NMRFAM-SPARKY: enhanced software for biomolecular NMR spectroscopy. *Bioinformatics* **31**, 1325-1327 (2015). https://doi.org/10.1093/bioinformatics/btu830
67 Allain, F., Mareuil, F., Ménager, H., Nilges, M. & Bardiaux, B. ARIAweb: a server for automated NMR structure calculation. *Nucleic Acids Res.* **48**, W41-W47 (2020). https://doi.org/10.1093/nar/gkaa362
68 Case, D. A. *et al.* The Amber biomolecular simulation programs. *J. Comput. Chem.* **26**, 1668-1688 (2005). https://doi.org/10.1002/jcc.20290
69 Amber 2025 (2025).
70 Schüttelkopf, A. W. & Van Aalten, D. M. F. PRODRG: a tool for high-throughput crystallography of protein–ligand complexes. *Acta Crystallogr. Sect. D. Biol. Crystallogr.* **60**, 1355-1363 (2004). https://doi.org/10.1107/s0907444904011679
71 Shen, Y. & Bax, A. Protein backbone and sidechain torsion angles predicted from NMR chemical shifts using artificial neural networks. *J. Biomol. NMR* **56**, 227-241 (2013). https://doi.org/10.1007/s10858-013-9741-y
72 Wang, J., Wolf, R. M., Caldwell, J. W., Kollman, P. A. & Case, D. A. Development and testing of a general amber force field. *J. Comput. Chem.* **25**, 1157-1174 (2004). https://doi.org/10.1002/jcc.20035
73 Wang, J., Wang, W., Kollman, P. A. & Case, D. A. Automatic atom type and bond type perception in molecular mechanical calculations. *J Mol Graph Model* **25**, 247-260 (2006). https://doi.org/10.1016/j.jmgm.2005.12.005
74 Lu, T. & Chen, F. Multiwfn: a multifunctional wavefunction analyzer. *J. Comput. Chem.* **33**, 580-592 (2012). https://doi.org/10.1002/jcc.22885
75 Zhang, J. & Lu, T. Efficient evaluation of electrostatic potential with computerized optimized code. *Phys. Chem. Chem. Phys.* **23**, 20323-20328 (2021). https://doi.org/10.1039/d1cp02805g
76 Neese, F. Software update: The ORCA program system—Version 5.0. *WIREs Computational Molecular Science* **12** (2022). https://doi.org/10.1002/wcms.1606
77 Neese, F. An improvement of the resolution of the identity approximation for the formation of the Coulomb matrix. *J. Comput. Chem.* **24**, 1740-1747 (2003). https://doi.org/10.1002/jcc.10318
78 Neese, F., Wennmohs, F., Hansen, A. & Becker, U. Efficient, approximate and parallel Hartree–Fock and hybrid DFT calculations. A 'chain-of-spheres' algorithm for the Hartree–Fock exchange. *Chem. Phys.* **356**, 98-109 (2009). https://doi.org/10.1016/j.chemphys.2008.10.036
79 Helmich-Paris, B., de Souza, B., Neese, F. & Izsak, R. An improved chain of spheres for exchange algorithm. *J. Chem. Phys.* **155**, 104109 (2021). https://doi.org/10.1063/5.0058766
80 Neese, F. The SHARK integral generation and digestion system. *J. Comput. Chem.* **44**, 381-396 (2023). https://doi.org/10.1002/jcc.26942
81 Izsak, R. & Neese, F. An overlap fitted chain of spheres exchange method. *J. Chem. Phys.* **135**, 144105 (2011). https://doi.org/10.1063/1.3646921
82 Neese, F. The ORCA program system. *WIREs Computational Molecular Science* **2**, 73-78 (2011). https://doi.org/10.1002/wcms.81

83 Izsák, R., Hansen, A. & Neese, F. The resolution of identity and chain of spheres approximations for the LPNO-CCSD singles Fock term. *Mol. Phys.* **110**, 2413-2417 (2012). https://doi.org/10.1080/00268976.2012.687466
84 Izsak, R., Neese, F. & Klopper, W. Robust fitting techniques in the chain of spheres approximation to the Fock exchange: The role of the complementary space. *J. Chem. Phys.* **139**, 094111 (2013). https://doi.org/10.1063/1.4819264
85 Neese, F. Software update: the ORCA program system, version 4.0. *WIREs Computational Molecular Science* **8** (2017). https://doi.org/10.1002/wcms.1327
86 Neese, F., Wennmohs, F., Becker, U. & Riplinger, C. The ORCA quantum chemistry program package. *J. Chem. Phys.* **152**, 224108 (2020). https://doi.org/10.1063/5.0004608
87 Neese, F. Approximate second-order SCF convergence for spin unrestricted wavefunctions. *Chem. Phys. Lett.* **325**, 93-98 (2000). https://doi.org/10.1016/s0009-2614(00)00662-x
88 Case, D. A. *et al.* AmberTools. *J Chem Inf Model* **63**, 6183-6191 (2023). https://doi.org/10.1021/acs.jcim.3c01153
89 Tian, C. *et al.* ff19SB: Amino-Acid-Specific Protein Backbone Parameters Trained against Quantum Mechanics Energy Surfaces in Solution. *J Chem Theory Comput* **16**, 528-552 (2020). https://doi.org/10.1021/acs.jctc.9b00591
90 Izadi, S., Anandakrishnan, R. & Onufriev, A. V. Building Water Models: A Different Approach. *J Phys Chem Lett* **5**, 3863-3871 (2014). https://doi.org/10.1021/jz501780a
91 Li, Z., Song, L. F., Li, P. & Merz, K. M., Jr. Systematic Parametrization of Divalent Metal Ions for the OPC3, OPC, TIP3P-FB, and TIP4P-FB Water Models. *J Chem Theory Comput* **16**, 4429-4442 (2020). https://doi.org/10.1021/acs.jctc.0c00194
92 Schmit, J. D., Kariyawasam, N. L., Needham, V. & Smith, P. E. SLTCAP: A Simple Method for Calculating the Number of Ions Needed for MD Simulation. *J Chem Theory Comput* **14**, 1823-1827 (2018). https://doi.org/10.1021/acs.jctc.7b01254
93 Machado, M. R. & Pantano, S. Split the Charge Difference in Two! A Rule of Thumb for Adding Proper Amounts of Ions in MD Simulations. *J Chem Theory Comput* **16**, 1367-1372 (2020). https://doi.org/10.1021/acs.jctc.9b00953
94 Roe, D. R. & Brooks, B. R. A protocol for preparing explicitly solvated systems for stable molecular dynamics simulations. *J. Chem. Phys.* **153**, 054123 (2020). https://doi.org/10.1063/5.0013849
95 Gotz, A. W. *et al.* Routine Microsecond Molecular Dynamics Simulations with AMBER on GPUs. 1. Generalized Born. *J Chem Theory Comput* **8**, 1542-1555 (2012). https://doi.org/10.1021/ct200909j
96 Salomon-Ferrer, R., Gotz, A. W., Poole, D., Le Grand, S. & Walker, R. C. Routine Microsecond Molecular Dynamics Simulations with AMBER on GPUs. 2. Explicit Solvent Particle Mesh Ewald. *J Chem Theory Comput* **9**, 3878-3888 (2013). https://doi.org/10.1021/ct400314y
97 Le Grand, S., Götz, A. W. & Walker, R. C. SPFP: Speed without compromise—A mixed precision model for GPU accelerated molecular dynamics simulations. *Comput. Phys. Commun.* **184**, 374-380 (2013). https://doi.org/10.1016/j.cpc.2012.09.022
98 Sindhikara, D. J., Kim, S., Voter, A. F. & Roitberg, A. E. Bad Seeds Sprout Perilous Dynamics: Stochastic Thermostat Induced Trajectory Synchronization in Biomolecules. *J Chem Theory Comput* **5**, 1624-1631 (2009). https://doi.org/10.1021/ct800573m
99 The PyMOL Molecular Graphics System, Version 3.1.6.1 (2025).

100 Roe, D. R. & Cheatham, T. E., 3rd. PTRAJ and CPPTRAJ: Software for Processing and Analysis of Molecular Dynamics Trajectory Data. *J Chem Theory Comput* **9**, 3084-3095 (2013). https://doi.org/10.1021/ct400341p
101 Adasme, M. F. *et al.* PLIP 2021: expanding the scope of the protein-ligand interaction profiler to DNA and RNA. *Nucleic Acids Res.* **49**, W530-W534 (2021). https://doi.org/10.1093/nar/gkab294
102 R: A Language and Environment for Statistical Computing (2025).
103 Mohamadi, F. *et al.* Macromodel—an integrated software system for modeling organic and bioorganic molecules using molecular mechanics. *J. Comput. Chem.* **11**, 440-467 (1990). https://doi.org/10.1002/jcc.540110405
104 PyMOL, The PyMOL Molecular Graphics System, Version 3.0 Schrödinger, LLC. (2025).
105 Schöning-Stierand, K. *et al.* Proteins Plus: a comprehensive collection of web-based molecular modeling tools. *Nucleic Acids Res.* **50**, W611-W615 (2022). https://doi.org/10.1093/nar/gkac305
106 Cianci, M. *et al.* P13, the EMBL macromolecular crystallography beamline at the low-emittance PETRA III ring for high- and low-energy phasing with variable beam focusing. *J. Synchrotron Radiat.* **24**, 323-332 (2017). https://doi.org/10.1107/s1600577516016465
107 CrysAlis PRO (2014).
108 Kabsch, W. XDS. *Acta Crystallogr. Sect. D. Biol. Crystallogr.* **66**, 125-132 (2010). https://doi.org/10.1107/s0907444909047337
109 Liebschner, D. *et al.* Macromolecular structure determination using X-rays, neutrons and electrons: recent developments in Phenix. *Acta Crystallogr. Sect. D. Biol. Crystallogr.* **75**, 861-877 (2019). https://doi.org/10.1107/s2059798319011471
110 Robinson, H. *et al.* The hyperthermophile chromosomal protein Sac7d sharply kinks DNA. *Nature* **392**, 202-205 (1998). https://doi.org/10.1038/32455
111 Afonine, P. V. *et al.* Towards automated crystallographic structure refinement with phenix.refine. *Acta Crystallogr. Sect. D. Biol. Crystallogr.* **68**, 352-367 (2012). https://doi.org/10.1107/s0907444912001308
112 Emsley, P. & Cowtan, K. Coot: model-building tools for molecular graphics. *Acta Crystallogr. Sect. D. Biol. Crystallogr.* **60**, 2126-2132 (2004). https://doi.org/10.1107/s0907444904019158
113 Moriarty, N. W., Grosse-Kunstleve, R. W. & Adams, P. D. electronic Ligand Builder and Optimization Workbench (eLBOW): a tool for ligand coordinate and restraint generation. *Acta Crystallogr. Sect. D. Biol. Crystallogr.* **65**, 1074-1080 (2009). https://doi.org/10.1107/s0907444909029436
114 Grade, Cambridge, United Kingdom, Global Phasing Ltd. (2011).
115 Wu, S. W., Ko, T. P., Chou, C. C. & Wang, A. H. Design and characterization of a multimeric DNA binding protein using Sac7d and GCN4 as templates. *Proteins: Struct. Funct. Bioinform.* **60**, 617-628 (2005). https://doi.org/10.1002/prot.20524
