# Supplementary material for: A protein–foldamer supramolecular synthon for self-assembled hybrid architectures
Source: Nat Chem. 2026 Aug 17;18(10):1819–27. doi: 10.1038/s41557-026-02222-6 (PMC13623658; doi:10.1038/s41557-026-02222-6)
Supplement: Supplementary file 2 [file 41557_2026_2222_MOESM2_ESM.pdf]

nature portfolio

Corresponding author(s): Ivan Huc

Last updated by author(s): Jun 5, 2026

Reporting Summary

Nature Portfolio wishes to improve the reproducibility of the work that we publish. This form provides structure for consistency and transparency in reporting. For further information on Nature Portfolio policies, see our Editorial Policies and the Editorial Policy Checklist.

Statistics

For all statistical analyses, confirm that the following items are present in the figure legend, table legend, main text, or Methods section.

n/a	Confirmed	
☒	☐	The exact sample size (*n*) for each experimental group/condition, given as a discrete number and unit of measurement
☒	☐	A statement on whether measurements were taken from distinct samples or whether the same sample was measured repeatedly
☒	☐	The statistical test(s) used AND whether they are one- or two-sided<br>*Only common tests should be described solely by name; describe more complex techniques in the Methods section.*
☒	☐	A description of all covariates tested
☒	☐	A description of any assumptions or corrections, such as tests of normality and adjustment for multiple comparisons
☒	☐	A full description of the statistical parameters including central tendency (e.g. means) or other basic estimates (e.g. regression coefficient) AND variation (e.g. standard deviation) or associated estimates of uncertainty (e.g. confidence intervals)
☒	☐	For null hypothesis testing, the test statistic (e.g. *F*, *t*, *r*) with confidence intervals, effect sizes, degrees of freedom and *P* value noted<br>*Give P values as exact values whenever suitable.*
☒	☐	For Bayesian analysis, information on the choice of priors and Markov chain Monte Carlo settings
☒	☐	For hierarchical and complex designs, identification of the appropriate level for tests and full reporting of outcomes
☒	☐	Estimates of effect sizes (e.g. Cohen's *d*, Pearson's *r*), indicating how they were calculated

Our web collection on statistics for biologists contains articles on many of the points above.

Software and code

Policy information about availability of computer code

Data collection

NMR data were collected using Topspin version 4.

Data analysis

Data Analysis/Collection, Processing & Modelling
* NMRPipe: Processing of multidimensional NMR spectra.
* Sparky (Version 3.190) / NMRFAM-SPARKY (Version 1.470): Analysis and assignment of NMR spectra.
* OriginPro (Version V.2019b): Processing and plotting of chromatography, CD spectroscopy, and BLI data.
* Octet Analysis Studio (Version V.13): Processing of BLI data and calculation of kinetic binding values.
* CrysAlisPro: Processing of X-ray diffraction data.
* XDS: Processing of X-ray diffraction data.
* PHASER: Solution of crystal structures via molecular replacement.
* PHENIX refine: Execution of iterative crystallographic structure refinement.
* COOT: Facilitation of real-space model building and evaluation of electron density.
* eLBOW: Generation of ligand coordinate and geometry restraint files.
* Grade2: Generation of ligand geometry restraints.
* PRODRG: Creation of parameter and topology files for foldamers.
* ARIA (Version 2.3): Iterative assignment of NOESY spectra and calibration of distance restraints in conjunction with CNS.
* CNS (Version 1.2): Calculation of initial NMR structures from linear conformations.
* TALOS-N: Prediction of protein backbone dihedral angles from NMR chemical shifts.
* Antechamber: Generation of partial charges and AMBER-compatible atom types.
* prepgen: Generation of topology and parameter prepin files.
* parmchk2: Retrieval of force field parameters for molecular dynamics.

* Multiwfn (Version 3.8): Execution of RESP charge calculations.
* Orca (Version 6.0): Execution of single-point energy calculations applying density functional theory (DFT).
* Amber (Version 24): Execution of restrained molecular dynamics simulations.
* Leap: Preparation of complexes for MD simulations within Amber.
* pmemd: Execution of energy minimization steps on CPU.
* pmemd.cuda: Execution of MD production runs on GPU.
* cpptraj (Version 6.18.1): Analysis of molecular dynamics trajectories.
* PLIP (Version 2.3.0): Profiling of protein-ligand interactions.
* PyMOL (Version 3.1.6): Visualization of molecules, alignment of structures, and calculation of solvent-accessible surface areas.
* ChemDraw (Version 25.0): Depiction of chemical structures.
* R (Version 4.5.1): Analysis of trajectory and ensemble data.
* Maestro MacroModel (Version 11.5): Execution of molecular modeling and energy minimizations.
* LifeSoaks: Calculation of pore bottlenecks via the proteins.plus interface.

For manuscripts utilizing custom algorithms or software that are central to the research but not yet described in published literature, software must be made available to editors and reviewers. We strongly encourage code deposition in a community repository (e.g. GitHub). See the Nature Portfolio guidelines for submitting code & software for further information.

Data

Policy information about availability of data

All manuscripts must include a data availability statement. This statement should provide the following information, where applicable:

- Accession codes, unique identifiers, or web links for publicly available datasets
- A description of any restrictions on data availability
- For clinical datasets or third party data, please ensure that the statement adheres to our policy

Data and materials availability: Crystallographic structures have been deposited at the Protein Data Bank under 9QDO, 9QT7, 9QOG & 9QNU. The ensemble of 20 structures calculated for the 1c-C10 complex have been deposited at the PDB under accession 9T0O. The chemical shift assignments for the unbound C10 and 1c•C10 complex were deposited in the BMRB (https://bmrb.io/) under accession numbers 53300 and 53317, respectively. BLI data have been deposited at Open Data LMU under https://data.ub.uni-muenchen.de/701/1/C10_BLI-raw_Data.zip. All other data are available in the main text or the supplementary materials.
Data and materials availability: Crystallographic structures have been deposited at the Protein Data Bank under 9QDO, 9QT7, 9QOG & 9QNU. The ensemble of 20 structures calculated for the 1c-C10 complex have been deposited at the PDB under accession 9T0O. The chemical shift assignments for the unbound C10 and 1c•C10 complex were deposited in the BMRB (https://bmrb.io/) under accession numbers 53300 and 53317, respectively. BLI data have been deposited at Open Data LMU under https://data.ub.uni-muenchen.de/701/1/C10_BLI-raw_Data.zip. All other data are available in the main text or the supplementary materials.

Research involving human participants, their data, or biological material

Policy information about studies with human participants or human data. See also policy information about sex, gender (identity/presentation), and sexual orientation and race, ethnicity and racism.

Reporting on sex and gender	*Use the terms sex (biological attribute) and gender (shaped by social and cultural circumstances) carefully in order to avoid confusing both terms. Indicate if findings apply to only one sex or gender; describe whether sex and gender were considered in study design; whether sex and/or gender was determined based on self-reporting or assigned and methods used. Provide in the source data disaggregated sex and gender data, where this information has been collected, and if consent has been obtained for sharing of individual-level data; provide overall numbers in this Reporting Summary. Please state if this information has not been collected. Report sex- and gender-based analyses where performed, justify reasons for lack of sex- and gender-based analysis.*
Reporting on race, ethnicity, or other socially relevant groupings	*Please specify the socially constructed or socially relevant categorization variable(s) used in your manuscript and explain why they were used. Please note that such variables should not be used as proxies for other socially constructed/relevant variables (for example, race or ethnicity should not be used as a proxy for socioeconomic status). Provide clear definitions of the relevant terms used, how they were provided (by the participants/respondents, the researchers, or third parties), and the method(s) used to classify people into the different categories (e.g. self-report, census or administrative data, social media data, etc.) Please provide details about how you controlled for confounding variables in your analyses.*
Population characteristics	*Describe the covariate-relevant population characteristics of the human research participants (e.g. age, genotypic information, past and current diagnosis and treatment categories). If you filled out the behavioural & social sciences study design questions and have nothing to add here, write "See above."*
Recruitment	*Describe how participants were recruited. Outline any potential self-selection bias or other biases that may be present and how these are likely to impact results.*
Ethics oversight	*Identify the organization(s) that approved the study protocol.*

Note that full information on the approval of the study protocol must also be provided in the manuscript.

Field-specific reporting

Please select the one below that is the best fit for your research. If you are not sure, read the appropriate sections before making your selection.

☒ Life sciences ☐ Behavioural & social sciences ☐ Ecological, evolutionary & environmental sciences

For a reference copy of the document with all sections, see nature.com/documents/nr-reporting-summary-flat.pdf

Life sciences study design

All studies must disclose on these points even when the disclosure is negative.

Sample size	*Describe how sample size was determined, detailing any statistical methods used to predetermine sample size OR if no sample-size calculation was performed, describe how sample sizes were chosen and provide a rationale for why these sample sizes are sufficient.*
Data exclusions	*Describe any data exclusions. If no data were excluded from the analyses, state so OR if data were excluded, describe the exclusions and the rationale behind them, indicating whether exclusion criteria were pre-established.*
Replication	*Describe the measures taken to verify the reproducibility of the experimental findings. If all attempts at replication were successful, confirm this OR if there are any findings that were not replicated or cannot be reproduced, note this and describe why.*
Randomization	*Describe how samples/organisms/participants were allocated into experimental groups. If allocation was not random, describe how covariates were controlled OR if this is not relevant to your study, explain why.*
Blinding	*Describe whether the investigators were blinded to group allocation during data collection and/or analysis. If blinding was not possible, describe why OR explain why blinding was not relevant to your study.*

Reporting for specific materials, systems and methods

We require information from authors about some types of materials, experimental systems and methods used in many studies. Here, indicate whether each material, system or method listed is relevant to your study. If you are not sure if a list item applies to your research, read the appropriate section before selecting a response.

Materials & experimental systems

n/a	Involved in the study	
☒	☐	Antibodies
☒	☐	Eukaryotic cell lines
☒	☐	Palaeontology and archaeology
☒	☐	Animals and other organisms
☒	☐	Clinical data
☒	☐	Dual use research of concern
☒	☐	Plants

Methods

n/a	Involved in the study	
☒	☐	ChIP-seq
☒	☐	Flow cytometry
☒	☐	MRI-based neuroimaging

Plants

Seed stocks	*Report on the source of all seed stocks or other plant material used. If applicable, state the seed stock centre and catalogue number. If plant specimens were collected from the field, describe the collection location, date and sampling procedures.*
Novel plant genotypes	*Describe the methods by which all novel plant genotypes were produced. This includes those generated by transgenic approaches, gene editing, chemical/radiation-based mutagenesis and hybridization. For transgenic lines, describe the transformation method, the number of independent lines analyzed and the generation upon which experiments were performed. For gene-edited lines, describe the editor used, the endogenous sequence targeted for editing, the targeting guide RNA sequence (if applicable) and how the editor was applied.*
Authentication	*Describe any authentication procedures for each seed stock used or novel genotype generated. Describe any experiments used to assess the effect of a mutation and, where applicable, how potential secondary effects (e.g. second site T-DNA insertions, mosiacism, off-target gene editing) were examined.*
